# From Hospital to Home: Applying a Co‐Design Approach to Determine the Key Components of an Intervention to Support Transition‐To‐Home After Stroke

**DOI:** 10.1111/hex.70040

**Published:** 2024-09-24

**Authors:** Geraldine O'Callaghan, Martin Fahy, Patricia Hall, Deirdre McCartan, Peter Langhorne, Rose Galvin, Frances Horgan

**Affiliations:** ^1^ iPASTAR Collaborative Doctoral Award Programme, School of Population Health RCSI University of Medicine and Health Sciences Dublin Ireland; ^2^ iPASTAR Collaborative Doctoral Award Programme, School of Physiotherapy RCSI University of Medicine and Health Sciences Dublin Ireland; ^3^ School of Public Health, Physiotherapy and Sports Science, Health Science Centre University College Dublin Dublin Ireland; ^4^ School of Cardiovascular and Metabolic Health (SCMH) University of Glasgow Glasgow UK; ^5^ School of Allied Health, Faculty of Education and Health Sciences, Ageing Research Centre, Health Research Institute University of Limerick Limerick Ireland

**Keywords:** co‐design process, hospital‐to‐home, patient and public involvement and engagement, stroke, transition

## Abstract

**Background:**

People with stroke and their families face numerous challenges as they leave hospital to return home, often experiencing multifaceted unmet needs and feelings of abandonment. The essential elements of an intervention intended to support transition‐to‐home after stroke are unclear.

**Objective:**

The aim of the project was to engage in a co‐design process to identify the key components of a pragmatic intervention to inform a transition‐to‐home support pathway following stroke.

**Materials and Methods:**

The study was conducted using a co‐design process engaging multiple stakeholders, including 12 people with stroke, 6 caregivers, 26 healthcare professionals and 6 individuals from stroke organisations in a series of three workshops, facilitated by the primary researcher, a wider team of researchers and an individual with lived experience of stroke. World Café methodology and Liberating Structures facilitation techniques were adapted to meet the aim of the workshops. Data collection involved observations during workshops, followed by summarising of findings and reaching group consensus agreement on outputs. Facilitated consensus on a prioritisation task resulted in the final output.

**Results:**

The co‐design group identified 10 key intervention components of a transition‐to‐home support pathway following stroke. These components focussed on enhancing collaboration, streamlining transition processes and facilitating post‐discharge support. While a stroke coordinator was considered a top priority, increased cross‐setting information sharing and community in‐reach, where community‐based healthcare staff extended their services into hospital settings to provide continuity care, were considered most feasible to implement.

**Conclusion:**

The co‐design approach, involving a multi‐stakeholder group and strengthened by patient and public involvement, ensured that the identified transition‐to‐home intervention components are meaningful and relevant for people with stroke and their families. Further co‐design workshops are required to refine, and feasibility test the components for generalisability within the wider Irish healthcare setting.

**Patient or Public Contribution:**

Individuals who have experienced a stroke actively contributed to shaping the methodological design of this study and the ethics process. They engaged in the analysis of co‐design outputs and provided input for the discussion and recommendations regarding future research. An individual who had experienced a stroke formed part of the research team, co‐facilitating the co‐design workshops and co‐authoring this article.

## 1 Introduction

The transition from hospital‐to‐home after a stroke represents a critical phase in the stroke rehabilitation journey. During this period, people with stroke (PWS) and their families must navigate numerous challenges, such as managing ongoing medical care, continuing physical rehabilitation, coping with emotional and psychological impacts, adapting to the home environment, coordinating with various healthcare providers and reintegrating into social networks. Many describe unmet needs and feelings of abandonment during this time [1, 2].

Recently, attention has focused on the processes for PWS leaving hospital care and returning to independent home living and adjusting to life after stroke [3, 4], and specifically on interventions that effectively support this complex transition [5, 6, 7]. One such intervention is early supported discharge (ESD), a transitional support programme providing PWS with a period of ongoing multidisciplinary rehabilitation at home. It reduces hospital stays and improves user satisfaction [6]; however there are gaps in information, caregiver support and transfer into community services [8], and limitations in who can access it as it is only suitable for those with mild‐moderate stroke [6]. There is uncertainty around the key components of alternative interventions designed to support transition‐to‐home after stroke [9]. A dedicated healthcare team member, such as a stroke coordinator or transition specialist, supports transition by facilitating communication, assisting with care plans and directing resources; however, there is insufficient evidence to support this model [10]. The COMPASS (Comprehensive Post‐Acute Stroke Services) trial, notable for adopting a pragmatic approach to implementation, offered post‐stroke follow‐up care to PWS, but failed to show a significant improvement in physical function and other outcomes after 90 days, despite providing individualised care plans, telephone and clinic follow‐up and 30‐ and 90‐day reviews [5]. By contrast, individualised information provided about stroke, ongoing recovery and expected supports at transition‐to‐home, was associated with positive transition experiences for both PWS and CGs [11]. However, comparing interventions that support home transition is challenging due to several factors, including the low methodological quality of the studies, their heterogeneity, inconsistency in the outcome measures used and the lack of applied intervention development frameworks [9]. Meaningful engagement of PWS, their families and other stakeholders in intervention development is also lacking [9].

To optimise recovery and ensure a successful transition‐to‐home for PWS and their families, a collaborative approach is required. Co‐design, which involves researchers collaborating with end users and other relevant stakeholders to create solutions to meet their needs, is increasingly recognised as best practice in healthcare and healthcare research [12, 13, 14]. Defined as ‘meaningful end‐user engagement in research design’ [14], a willingness by participants to understand each other's experiences and viewpoints is essential, with values of equity, inclusivity, partnership and empowerment fundamental to the process [15]. By engaging end‐users in healthcare research, co‐design potentially mitigates waste often seen in traditional health research, such as excluding relevant perspectives or including outcomes irrelevant to specific groups [16]. This approach is particularly valuable in developing patient‐centred pragmatic interventions, which are designed to seamlessly integrate into clinical practice and leverage existing resources, ensuring that the evidence generated is directly applicable to real‐world healthcare scenarios, an essential consideration in today's resource‐constrained healthcare environment [17].

The aim of this study was to utilise a co‐design process to identify key components for a patient‐centred transition‐to‐home support pathway, exploring what might be feasible within a real‐world context. The process was guided by the ‘Development’ phase of the Medical Research Council (MRC) framework for developing and evaluating complex interventions [18], with key stages within that phase completed, published and informing this co‐design process [9, 19, 20].

## 2 Materials and Methods

### 2.1 Design

**Figure 1:**
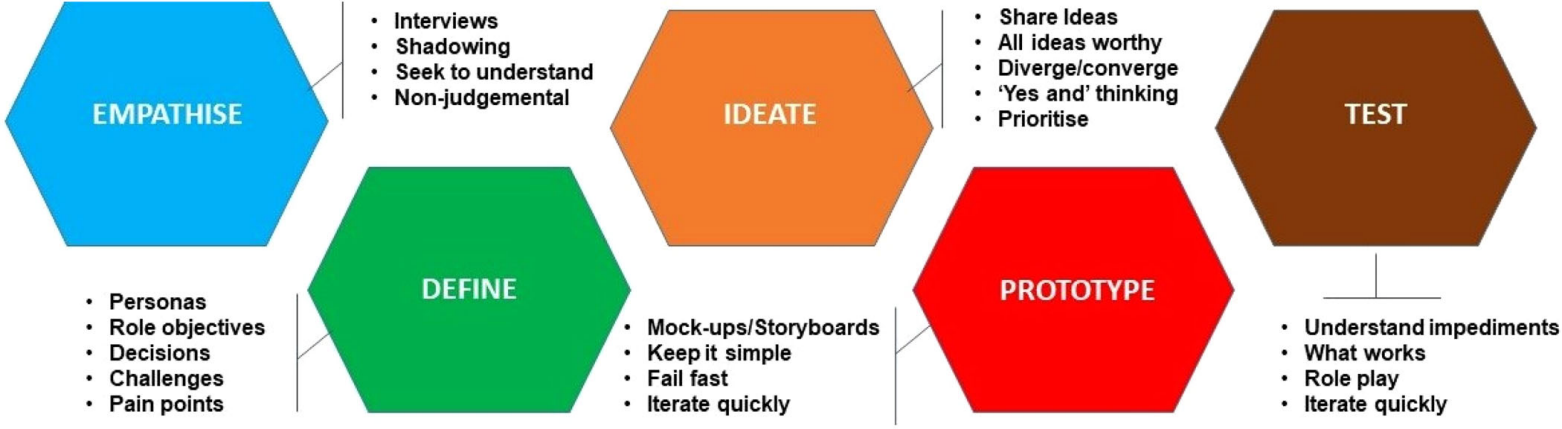
Design Thinking Process model by Stanford d.school setting.

We employed a co‐design process guided by Design Thinking to identify key components of an intervention facilitating the transition from hospital‐to‐home for individuals with stroke. Design Thinking is a human‐centred and user‐driven development approach that emphasises creativity, collaboration and iterative problem‐solving, with a focus on empathy and innovation to co‐create solutions [21, 22]. It is considered a suitable approach to address complexity in a health system [12]. We used the Stanford d.school method of Design Thinking (Figure 1) [23], which has five phases: (1) Empathise: Understand User Needs; (2) Define: Define The Problem; (3) Ideate: Generate Solutions and (4) Prototype: Make Your New Idea Tangible. The last phase is the testing phase: (5) Test: Get Feedback From User, which is presently in the planning stage and is not described in this article.

The co‐design process took place in a semi‐rural Irish setting where ESD is not currently available. Following acute and sub‐acute stroke care the majority of those aged > 65 can access in‐patient rehabilitation locally, while those < 65 can face long waits in hospital or at home for access to a national in‐patient rehabilitation service. Community care options include specialised community rehabilitation, which provides outpatient or community‐based services for individuals with neurological conditions, such as stroke, and generic primary care services offering basic healthcare either at home or in a clinic to the general community. Additionally, community intervention teams aim to expedite hospital discharge by providing short‐term, nurse‐led care at home for those with complex health and social care needs.

### 2.2 Participants

#### 2.2.1 PWS and Their Caregivers (CGs)

PWS and their CGs were recruited in conjunction with a national stroke advocacy organisation: The Irish Heart Foundation, and from one of three sites engaged in an observational cohort study that profiled outcomes and needs of individuals with stroke transitioning from hospital‐to‐home [19]. The criteria for inclusion were people who (a) had experienced a stroke and a subsequent care transition from hospital‐to‐home; and (b) were able to communicate or be supported to communicate in group discussions. CGs were those providing informal care or support to a PWS meeting the inclusion criteria. Further details about this group are available in Table 1 and Supporting Information S1: Material I.

**Table 1:** Participants who consented and engaged in the co‐design process.

Participant type	Consented	Participant information	Attended workshop 1	Attended workshop 2	Attended workshop 3
People with stroke	*N* = 12	Under 65 (*n* = 6) Over 65 (*n* = 6) Male (*n =* 10) Female (*n =* 2)	11		10
Caregivers (CGs)	*N* = 6	Informal caregiver (*n* = 5) Formal caregiver (*n* = 1) Male (*n* = 0) Female (*n* = 6)	4		5
Healthcare professionals (HCPs)	*N* = 26	Acute Stroke Team (*n* = 5; stroke consultant, CNS, PT, SW) Rehabilitation Team (*n* = 3; PT, OT, SLT) Generic Primary Care (*n* = 9; PHN, PT, OT, SW, Psychology) Community Intervention Team (*n = *3; PT, OT) Specialist Community Rehabilitation (*n* = 6; PT, OT)		24	18
Individuals from other rehabilitation and support services	*N* = 6	Irish Heart Foundation (IHF) (*n* = 2) Acquired Brain Injury (ABI), Ireland (*n =* 1) Adult disability day service (*n* = 2) National Learning Network (*n* = 1)		5	3

Abbreviations: CNS, clinical nurse specialist; OT, occupational therapist; PT, physiotherapist; SLT, speech and language therapist; SW, social worker.

#### 2.2.2 Multi‐Sectoral Stakeholder Group

Programme managers and support staff from the national stroke advocacy organisation, a brain injury rehabilitation service, a day service for people with disabilities and an education and vocational training organisation, all of whom support PWS in the region as they transition‐to‐home, were purposely recruited. Healthcare professionals (HCPs), representing individuals working in acute stroke care, in‐patient rehabilitation and community services representative of specialised community rehabilitation, community intervention teams and generic primary care, were purposely recruited. Oral and written information about the co‐design process was provided to all participants, and written informed consent was obtained from all participants. PWS and CGs who attended were offered reimbursement in the form of a voucher for their time, and transport was facilitated as required. No reimbursement was provided to the individuals in the multi‐sectoral stakeholder group.Further details about this group can be found in Table 1 and Supporting Information S1: Material I.

Ethical approval was obtained from the Research Ethics Committee Midlands Area and Corporate (Regional Health Area B) (ref: RREC0423FH).

### 2.3 Co‐Design Process

Identifying key components of an intervention to support PWS and their families at transition from hospital‐to‐home.

Three half‐day workshops were held in a hotel conference room in June 2023. We adopted a staggered approach to the workshops: healthcare providers and those from support agencies attended workshop 1, and PWS and CGs attended workshop 2. This approach helped build confidence and engagement, and minimised potential power imbalances, establishing the foundation for effective collaborations when both groups engaged for the final collaborative dialogue in workshop 3. To facilitate discussion and creative collaboration, participants at each workshop were purposively divided into distinct subgroups, ensuring each stakeholder category was represented in every subgroup. A doctoral student known to the primary researcher (G.O'.C.) was assigned to each subgroup to support participants with physical (*n* = 2) and communication (*n* = 1) challenges to participate. All subgroup discussions were integrated into a larger group to arrive at common agreed outputs at the end of each workshop session. The workshops were facilitated by the primary researcher (G.O'.C.), a female physiotherapist and doctoral student, alongside co‐facilitators, also female health professionals and doctoral students (PH, DMc), and a PPIE stroke champion (M.F.), who had experienced a stroke. As part of their postgraduate education, doctoral facilitators had received training in design thinking and the adopted methodologies. All facilitators met before and between workshops, to plan, structure and deliver them. Each workshop comprised unique elements and methodologies. World Café methodology and Liberating Structures facilitation techniques were adapted to meet the aim of the workshops. World Café is a conversational process that facilitates dialogue and collaboration as a diverse group of participants sit around a round table to generate new ideas and insights [24]; while Liberating Structures is a collection of 33 facilitation techniques that are combined, and build upon each other, to help groups collaborate, innovate and make decisions [25]. Regular breaks were interspersed throughout each workshop.

**Figure 2:**
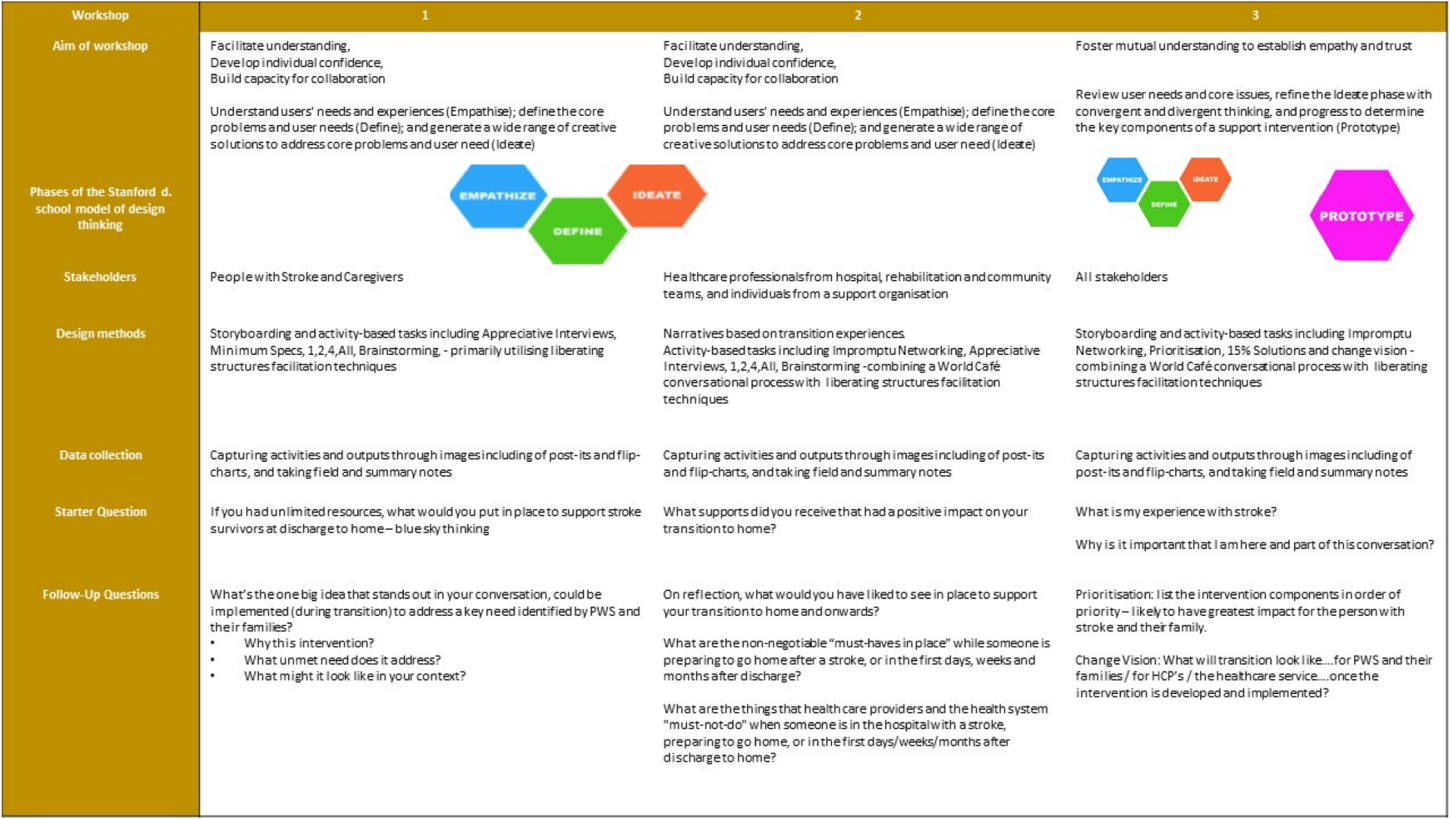
Overview of the Design Thinking Process, including guiding questions for facilitating ideation and consensus building.

Figure 2 provides an overview of the co‐design process and outlines guiding questions for ideation and consensus building. Workshop slides, including instructions for activities and tasks, can be found in Supporting Information S1: Material II.

### 2.4 Workshops 1 and 2: Envisaging an Ideal Transition From Hospital‐To‐Home

Workshops 1 and 2 focussed on the Empathise, Define and Ideate phases of design thinking. The phases were developed through (1) narratives based on individual needs and transition support experiences and preferences [19, 20], and (2) activity‐based tasks, including mind mapping, brainstorming and storyboarding, where a large number and variety of ideas for components that would support transition home after stroke were generated. The activities in workshop 1 and 2 are outlined in Supporting Information.

Following workshops 1 and 2, facilitators convened to debrief and align on key takeaways, and to refine plans for the 3rd workshop.

### 2.5 Workshop 3: Generating and Prioritising Solutions for Real‐World Implementation

**Figure 3:**
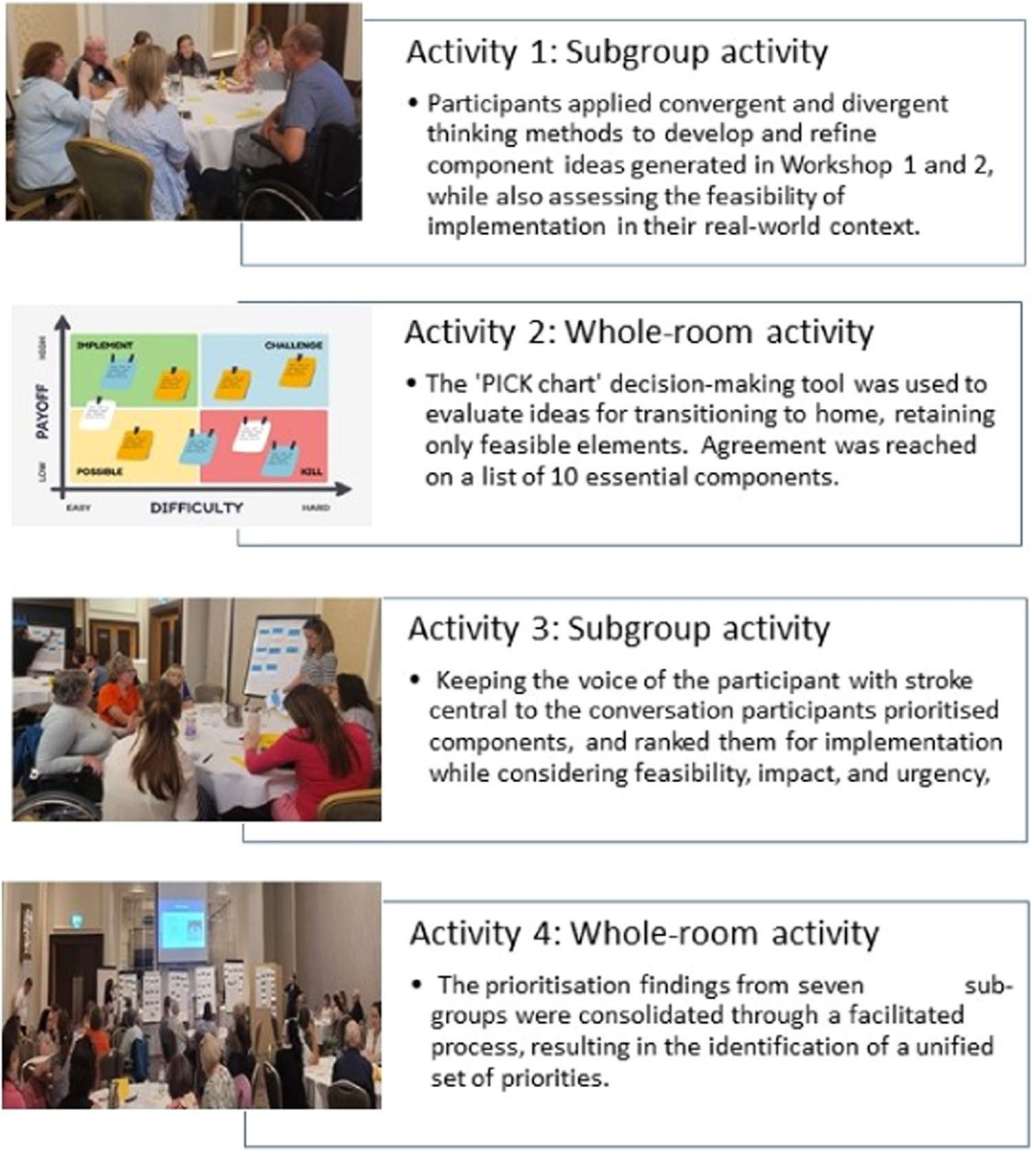
Activities and tasks in workshop 3.

The participants merged in Workshop 3. Time was spent initially building shared empathy and trust. During this workshop, participants engaged in four activities. The goal was to further develop (Ideate) and agree with the components of an intervention, which would support transition‐to‐home and could be implemented in their real‐world context. The activities in workshop 3 are outlined in Figure 3, with further detail in Supporting Information S1: Material II.

In Activity 1, stakeholders were purposively divided into seven multi‐stakeholder subgroups and asked individually, and then collectively, to apply convergent and divergent thinking methods to develop and refine component ideas. They were asked to consider the feasibility, including the expected facilitators and barriers, for implementing each component within their real‐world scenarios.

In Activity 2, the subgroups reconnected into the larger group in the shared physical space to consolidate discussion. Using a decision‐making tool ‘PICK chart’ (P: Possible (or Potential); Impact; I: Implementation Effort; C: Challenge; K: Keep or Kill), ideas were discussed in relation to their potential impact and the resource required for implementation, keeping only those elements considered feasible to implement or deemed too important in supporting the transition to be completely dismissed. A guided consultation process was then utilised to agree on a comprehensive list of components considered important in supporting transition‐to‐home.

In Activity 3, the identified components were recorded on individual cards by the primary researcher, with a complete set of cards allocated to each multi‐stakeholder subgroup. While in their subgroups participants were then asked to use a patient‐centred approach to prioritise the key components, again considering factors, such as feasibility, impact and urgency, and then ranking them in numerical order of importance to implement.

Finally, in Activity 4, in a whole‐room consultation process, the prioritisation findings from the seven subgroups were consolidated through a facilitation process. Agreements and divergence were addressed and discussions continued, both in small groups and across the whole group, until consensus was reached and a unified set of priorities emerged, which marked the end of the co‐design process.

### 2.6 Data Collection

Workshop data were collected using anonymous data collection tools such as post‐its and flip‐charts, and field and summary notes. Any identifiable information was redacted, and individual responses were aggregated. At the close of each workshop, the primary researcher delivered a brief presentation summarising aggregated discussions, insights and key decisions/outputs. Participants provided comments, feedback and final sign‐off on the outputs, either at the end of each workshop or by contacting the primary researcher after a period of reflection, within a 1‐week time‐frame. The original data were managed in line with relevant data protection requirements.

### 2.7 Analysis

The prioritised components were translated into themes through a systematic process of collaborative discussions with co‐facilitators and the PPIE panel, alongside practicing reflexivity with PhD supervisors (F.H. & R.G.), and critically evaluating the process to develop a shared understanding of the outputs. The final draft of the transition‐to‐home stroke pathway was presented to the PPIE panel to gather feedback, insights and perspectives based on their lived experiences.

### 2.8 Patient and Public Involvement and Engagement (PPIE)

Four PWS, ‘stroke champions’, were recruited to a PPIE panel to ensure their perspectives and priorities were integrated into the research process. The Guidance for Reporting Involvement of Patient and Public version 2 short form (GRIPP2‐SF) [26] was used (Supporting Information S1: Material III). PPIE contributed to the methodological development and ethics submission for this study. Meetings took place in person or using a video platform (MSTeams). They helped to refine the structure, plans, timing and delivery of the workshop elements. One ‘stroke champion’ engaged as a co‐facilitator during the workshop, while the wider PPIE panel contributed to the analysis.

## 3 Results

Twelve individuals with stroke; six CGs, including a formal CG; and 26 healthcare professionals (five from the acute stroke team; three from rehabilitation and eighteen from the community) participated. There were two representatives from a stroke organisation, two programme managers from a brain injury rehabilitation programme and an education and training rehabilitation agency; and two programme managers from a day service for people with disability **(**Table 1 **).** Detailed demographics are found in Supporting Information S1: Material I.

**Figure 4:**
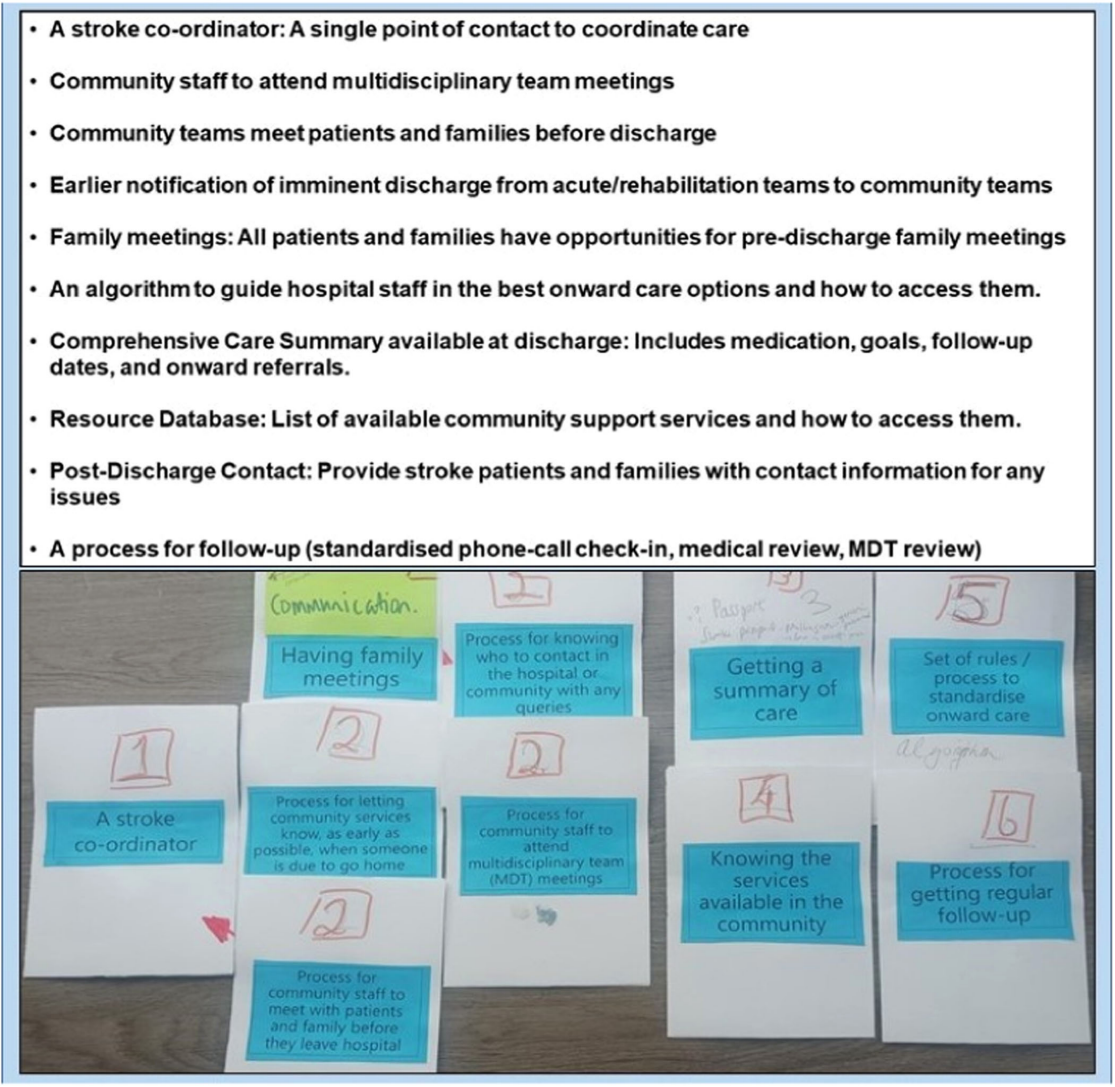
Ten intervention components, followed by ranking from 1 to 6.

In workshop 3, 10 intervention components (Figure 4 **)** were identified as crucial for supporting the transition from hospital‐to‐home after stroke. In the prioritisation phase (Activities 3 & 4), stakeholders found some components equally important, making it difficult to rank them in a strict sequential order. Following the facilitated discussion and consensus process described in Activity 4, the 10 components were ranked overall on an overall scale of 1–6, with some sharing the same rank (Figure 4). The identified components included (1) The presence of a stroke coordinator as overarching support to facilitate coordination and continuity of care during this period; (2) Enhanced communication and information sharing through earlier notification to community teams of imminent discharge, community teams taking part in multidisciplinary team meetings and meeting the PWS before discharge, family meetings, and a process enabling the person with stroke and family to know who to contact with any queries or issues post‐discharge; (3) A comprehensive summary of care, including goals and ongoing plans, available at discharge; (4) A centralised accessible database signposting PWS, families and HCPs to community‐based resources; (5) An algorithm to assist hospital‐based HCPs to determine the most appropriate rehabilitation services to direct PWS to on‐discharge; and (6) A structured process for providing periodic but regular medical and multidisciplinary review and follow‐up.

Once workshops were complete, the research team, including clinical and academic experts and a PPIE ‘stroke champion’, engaged with the primary researcher (G.O'.C.) to group intervention components into categories based on the team's shared understanding of the intended purpose of each component. Facilitating communication and information sharing; optimising workflows to expedite and ensure a smooth transition‐to‐home; and providing tailored support to address the ongoing needs of PWS post‐discharge, were among the agreed intended purposes of the intervention components (Supporting Information S1: Material IV). The PPIE panel endorsed the proposed categorisation: Enhance collaboration, Streamline transition processes, Facilitate post‐discharge support, and Embed a stroke coordinator in the pathway as overarching support.

**Figure 5:**
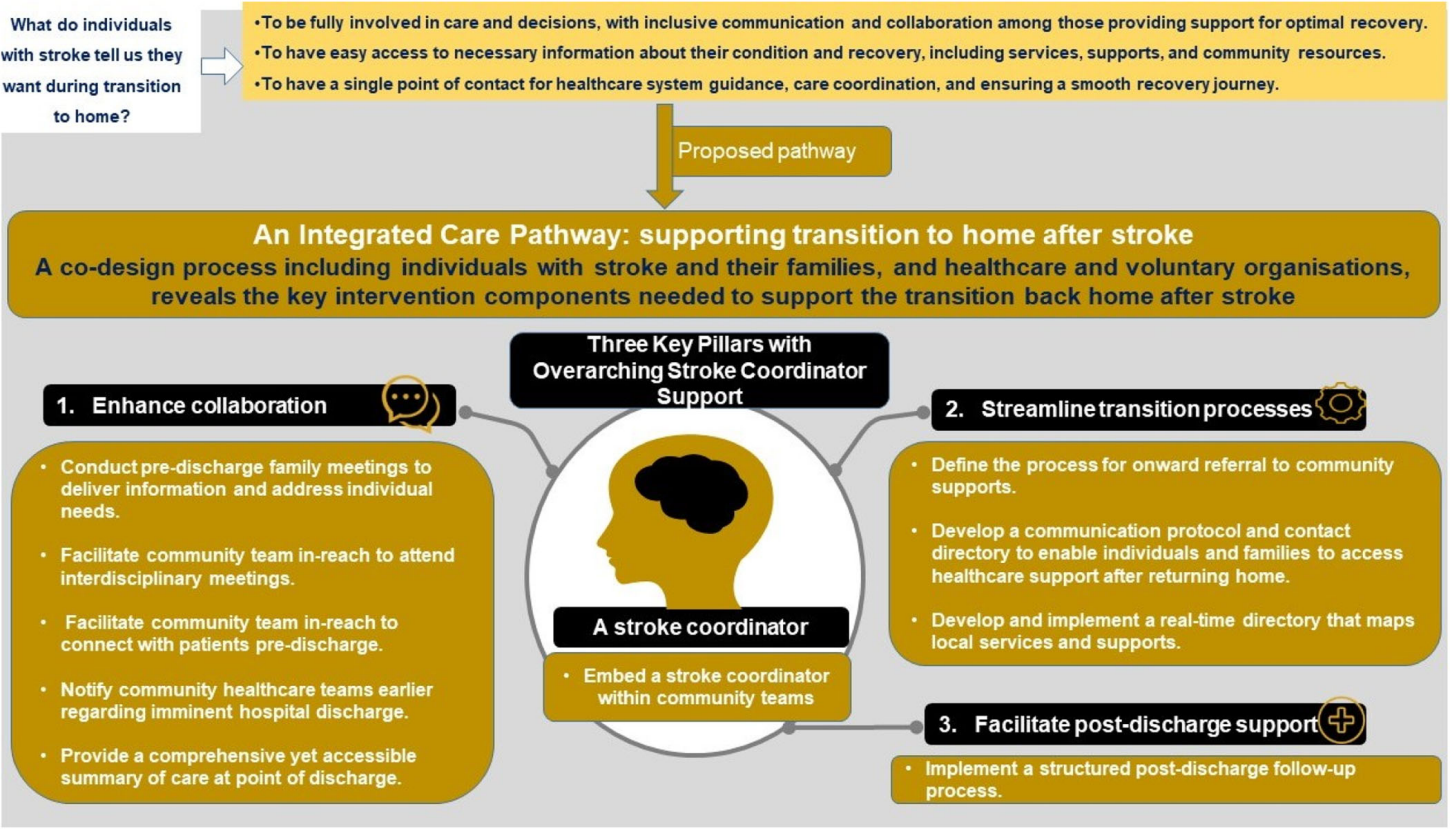
Transition‐to‐home support pathway.

While a stroke coordinator was perceived as the top priority to implement, intervention components within ‘Enhance collaboration’ were perceived as crucial and the most feasible to implement. The intervention components derived from the co‐design process shape a proposed pathway to support transition‐to‐home after stroke (Figure 5).

### 3.1 PPIE

The PPIE ‘stroke champions’ provided final approval on the co‐design outputs, confirming the proposed solutions aligned with the needs of PWS. They also made suggestions for further research, such as assessing whether these same outputs would be recommended in a different healthcare context, which would then inform the planning stage for the testing phase.

## 4 Discussion

This study outlines the process and outputs of a co‐design approach involving PWS, CGs, HCPs and those from support organisations, to identify 10 key components essential for supporting the transition from hospital‐to‐home post‐stroke. These align into four categories: Enhance collaboration, Streamline transition processes, Facilitate post‐discharge support, and Embed a stroke coordinator in the pathway. The study suggests collaborative activities such as increasing information sharing between healthcare providers and PWS as well as across settings, and providing continuity of care by extending community‐based healthcare services into hospital settings, are the most feasible to implement. A stroke coordinator providing coordination and continuity of care is deemed the highest priority.

Co‐design participants emphasised the stroke coordinator as a central figure during transition. Literature where a coordinator was fundamental to the model supporting transition‐to‐home varies widely with regard to duration of intervention and intervention descriptions [27], with mixed results regarding the impact of a stroke coordinator on healthcare outcomes [7, 28, 29]. One study suggested that any observed benefits may not solely be attributed to the presence of a coordinator or transition specialist but could be influenced by other factors or interventions [7]. Despite this, the Movement Interventions Task Force in America recommends integrating a dedicated transition specialist into community‐based healthcare teams, to enhance support for PWS and contribute to a holistic approach to stroke rehabilitation and recovery [30]. Integrating this role into an already overburdened Irish healthcare system may be challenging, as uncertainty surrounds governance and the definition and scope of responsibilities for transition specialists. This uncertainty could lead to inconsistent implementation and outcomes. Further research is needed to clearly define the stroke coordinator role and to determine effectiveness, feasibility and sustainability.

The co‐design process emphasised enhanced collaboration as crucial for supporting transition‐to‐home after stroke, with efficient communication and information‐sharing fostering trust among all stakeholders. The literature indicates that PWS and their families require trusting relationships with HCPs and clear communication to address concerns about returning home and to dispel misconceptions about recovery. However, communication is often inadequate for the complex care and recovery needs of PWS [31]. In Australia, community‐based HCPs note challenges in accessing acute stroke care plans as a barrier to continuity of care, while a shared understanding of the needs of PWS is identified as an enabler [32]. Increased information sharing among stakeholders by establishing a shared vision, building relationships, such as between commissioners, voluntary agencies and health and social care professions, and implementing diverse communication mechanisms such as family conferences and ad‐hoc exchanges, are crucial for enhancing interprofessional and patient‐professional collaborations [27, 33, 34] and supporting PWS at transition‐to‐home [27]. Interestingly, increasing primary care team size, centralised administrative processes and mixed governance can negatively impact collaboration [33]. Increasing community HCPs in‐reach offers additional opportunities, such as promoting skill‐sharing and expertise exchange, ensuring a smooth transition [31]. In agreement with our co‐design participants, a structured discharge summary is considered essential for a smooth transition from hospital to community care, with the information that should be included in this summary documented in some stroke guidelines and elsewhere [35, 36]. However, adherence to content advice varies [35].

Streamlining transition processes was emphasised throughout the co‐design process as being essential to facilitating transition home for a PWS. The intervention components within this theme are not directly tied to facilitating the transition‐to‐home, but rather, are broader organisational changes that could support the implementation of communication‐enhancing interventions, serving as the structural backbone for enhanced collaboration. They optimise workflows, creating a smoother pathway for communication, and are likely context‐specific. Recognising the importance of an integrated care approach for community reintegration following a stroke, some work has been undertaken to map community rehabilitation and support services available for PWS [37, 38]. In the United Kingdom, post‐ESD services mapping was restricted to what the participating ESD teams knew, and did not include information from individuals working within community services; therefore, it is unlikely that the output provided a complete picture of what was available [37]. A Service Mapping tool was developed through a partnership between the Health Service Executive, Disability Federation of Ireland, and Neurological Alliance of Ireland to facilitate the integration of over 60 voluntary organisations supporting neurological conditions in Ireland with community‐based services [38]. While an extremely valuable initiative, the process of mapping itself, and scaling it up on a national level, presents several challenges, including data accessibility, geographic disparities, resource limitations and sustainability. Overcoming these challenges is likely to require a comprehensive, cooperative and technology‐driven approach.

Co‐design participants emphasised the need for a post‐discharge follow‐up process to provide ongoing support and address emerging issues. According to the literature, post‐stroke issues can persist for years if untreated, impacting prognosis and quality of life [1, 39]. The United Kingdom and Ireland Stroke Guidelines suggest structured reassessment at 6 months post‐discharge and annually, considering physical, psychological and social needs [40]; however, current post‐discharge follow‐up in Ireland is unclear since this information is not captured in stroke audits [41]. The Post‐stroke Checklist (PSC) aimed to standardise post‐stroke reviews [42]; however, it has not been widely implemented in routine stroke care. A more recent study evaluated the feasibility of a 3‐month follow‐up using a modified PSC, revealing that the comprehensive approach effectively captures most stroke‐related health issues and is efficient to implement [43].

Many of the intervention components determined by our co‐design process involve prioritising patient‐centred transition practices that require organisational restructuring, without necessarily demanding extra resources. Further developing and implementing these intervention components in a real‐world setting warrants consideration of the practical elements, such as resource constraints, resistance to change, fragmented systems and technological support gaps. While Duncan's COMPASS intervention did not lead to a positive shift in physical function and other outcomes 90 days after discharge home, those who actually participated in the intervention had better outcomes in terms of survival, satisfaction with care, depression, disability and falls, than those who did not engage, and there was an increase in the number self‐monitoring their own blood pressure at home [5]. The strength of the COMPASS model lay in its patient‐centred co‐design approach, which involved relevant stakeholders in the programme's design, implementation and sustainability. Further pivotal work involving end‐users in the UK and Sweden underpinned the development of pragmatic interventions aimed at supporting transition‐to‐home and community integration after stroke [36, 44]. In all trial's implementation was not without challenges. In the COMPASS trial, despite careful planning, oversight and support, numerous difficulties were encountered, including maintaining adequate staffing and incomplete case identification in hospitals; low participant engagement and completion rates for in‐person clinic visits; and significant loss to follow‐up [5]. Given these challenges, it is essential to consider enablers to ensure successful implementation of the transition‐to‐home support pathway identified in this co‐design process. The literature suggests that strong cross‐organisational leadership is crucial for increasing implementation capacities in complex interventions [33], as there is typically at least one limiting factor or bottleneck, such as people, equipment, IT systems or policies that impact the implementation process [45].

### 4.1 Strengths and Limitations

The study's use of a co‐design process, which enabled end‐users to actively participate in a solution‐oriented process aimed at addressing the challenges faced during the transition home, was one of its main strengths. PWS and CGs appreciated the opportunity to improve post‐stroke experiences and outcomes, while HCPs and individuals from support organisations valued the chance to view challenges from the perspective of a PWS and contribute to patient‐centred solutions. The participation of a PPIE panel with lived experience was a strength. They offered invaluable guidance and support, enriched discussions and decision‐making and ensured that the process maintained a patient‐centred focus, with more meaningful outcomes. Additionally, having one PPIE member as a co‐facilitator contributed to a more supportive workshop environment, resulting in deeper discussions and more impactful results. However, one associated challenge was ensuring that the PPIE co‐facilitator could navigate the transition from a person with lived experience to a leader in a solution‐focused process, avoiding potential blurred boundaries in terms of objectivity and focus. To mitigate this, we engaged the PPIE co‐facilitator as part of the workshop planning team, set clear guidelines for their role, and provided specific training on maintaining neutrality through the process.

A key limitation of this study was its narrow focus, which identified the components (what) for the prototype, but did not delve into the individuals involved (who), the locations (where), timing (when), and implementation of each component (how). Further engagement with the co‐design participants should explore these aspects within each identified component. Furthermore, the research's limited scope in a single healthcare setting, where the expectation and experiences of the participants may have influenced their thoughts on service design, potentially introduces bias and limits generalisability of the outputs. Further exploration is required to determine the transferability of the transition‐to‐home support pathway across diverse contexts. Finally, the absence of a recognised analytical framework may be considered a limitation; however, the iterative and collaborative nature of our analysis ensured a thorough and nuanced understanding of the data, fostered critical discussion, and enhances the construct validity and credibility of the findings.

## 5 Conclusion

The aim of the project was to identify the key components of a stroke transition‐to‐home support pathway. Using a co‐design process, we fostered collaborative dialogue to uncover 10 key support intervention components, merged into six and explored within four intervention categories: Enhance collaboration, Streamline transition processes, Facilitate post‐discharge support, and Embed a stroke coordinator in the pathway. Further co‐design work is necessary to go beyond the ‘what’ of these elements, to further refine and examine them for acceptance and feasibility within the study context and across a wider range of Irish healthcare settings, ultimately subjecting them to feasibility testing.

## Author Contributions

**Geraldine O'Callaghan:** Conceptualisation, investigation, methodology, writing–original draft, visualisation, formal analysis, project administration, data curation, validation. **Martin Fahy:** Writing–review & editing, methodology, funding acquisition, conceptualisation, formal analysis, investigation. **Patricia Hall:** Writing–review & editing, investigation, formal analysis. **Deirdre McCartan:** Writing–review & editing, investigation, formal analysis. **Peter Langhorne:** Writing–review & editing, Supervision. **Rose Galvin:** Conceptualisation, Writing–review & editing, methodology, formal analysis, supervision, validation. **Frances Horgan:** Conceptualisation, methodology, funding acquisition, formal analysis, supervision, writing–review & editing, validation.

## Conflicts of Interest

The authors declare no conflicts of interest.

## Supporting information

Supporting information.

## Data Availability

The datasets used and/or analysed during the current study are available from the corresponding author upon reasonable request (gocallaghan@rcsi.com).
