# Supplementary material for: From Hospital to Home: Applying a Co‐Design Approach to Determine the Key Components of an Intervention to Support Transition‐To‐Home After Stroke
Source: Health Expect. 2024 Sep 24;27(5):e70040. doi: 10.1111/hex.70040 (PMC11420660; doi:10.1111/hex.70040)
Supplement: Supplementary file 1 — Supporting information. [file HEX-27-e70040-s001.docx]

**From Hospital to Home: Applying a Co-design Approach to Determine the Key Components of an Intervention to Support Transition-to-Home After Stroke.**

**SUPPLEMENTAL MATERIAL**

**Supplemental material I** Demographic data

PWS and CGs

Multi-sectoral stakeholder group

**Supplemental material II** Workshop slides including activities and tasks

**Supplemental material III** GRIPP2-SF

**Supplemental material IV** Aligning components into categories with narrative overview

**Supplemental material I**. Demographic data

Demographic data People with stroke (PWS) and Caregiver (CG)

| **People with stroke (PWS)** | | | | | |
| --- | --- | --- | --- | --- | --- |
| **Participant ID** | **Sex** | **Age <65/>65** | **Number of workshops** | **Workshop 2** | **Workshop 3** |
| **PWS 1** | M | >65 | 2/2 | Yes | Yes |
| **PWS 2** | M | <65 | 2/2 | Yes | Yes |
| **PWS 3** | M | >65 | 1/2 | Yes | No |
| **PWS 4** | F | <65 | 2/2 | Yes | Yes |
| **PWS 5** | M | >65 | 2/2 | Yes | Yes |
| **PWS 6** | M | <65 | 2/2 | Yes | Yes |
| **PWS 7** | M | >65 | 2/2 | Yes | Yes |
| **PWS 8** | F | <65 | 2/2 | Yes | Yes |
| **PWS 9** | M | >65 | 2/2 | Yes | Yes |
| **PWS 10** | M | >65 | 2/2 | Yes | Yes |
| **PWS 11** | M | <65 | 1/2 | No | Yes |
| **PWS 12** | M | >65 | 1/2 | Yes | No |

| **Caregiver (CG)** | | | | | | |
| --- | --- | --- | --- | --- | --- | --- |
| **Participant ID** | **Relationship to PWS** | **Sex** | **Age <65/>65** | **Number of workshops** | **Workshop 2** | **Workshop 3** |
| **CG1** | Significant other | F | <65 | 1/2 | No | Yes |
| **CG2** | Formal caregiver | F | <65 | 2/2 | Yes | Yes |
| **CG3** | Significant other | F | >65 | 2/2 | Yes | Yes |
| **CG4** | Significant other | F | >65 | 2/2 | Yes | Yes |
| **CG5** | Significant other | F | >65 | 1/2 | Yes | No |
| **CG6** | Sister | F | <65 | 1/2 | No | Yes |

Demographic data Healthcare Professionals and participants from advocacy/ support organisations

| **Healthcare Professionals** | | | | | |
| --- | --- | --- | --- | --- | --- |
| **Participant ID** | **Profession** | **Work Setting** | **Number of workshops** | **Workshop 1** | **Workshop**  **3** |
| **H1** | PT | In-patient rehabilitation | 2/2 | Yes | Yes |
| **H2** | OT | Community – specialist rehabilitation | 2/2 | Yes | Yes |
| **H3** | SLT | Community – generic primary care | 2/2 | Yes | Yes |
| **H4** | OT | In-patient  rehabilitation | 2/2 | Yes | Yes |
| **H5** | PT | Community – specialist rehabilitation | 2/2 | Yes | Yes |
| **H6** | SW | Acute stroke | 1/2 | Yes | No |
| **H7** | PHN | Community | 2/2 | Yes | Yes |
| **H8** | CNS | Acute stroke | 1/2 | No | Yes |
| **H9** | OT | Community – specialist rehabilitation | 2/2 | Yes | Yes |
| **H10** | OT | Community – generic primary care | 2/2 | Yes | Yes |
| **H11** | PT | Community – specialist rehabilitation | 2/2 | Yes | Yes |
| **H12** | PT | Community – generic primary care | 1/2 | Yes | No |
| **H13** | PT | Community – generic primary care | 1/2 | Yes | No |
| **H14** | PT | Acute stroke | 2/2 | Yes | Yes |
| **H15** | PT | Community intervention team | 2/2 | Yes | Yes |
| **H16** | PT | Community – specialist rehabilitation | 1/2 | Yes | No |
| **H17** | SLT | In-patient rehabilitation | 2/2 | Yes | Yes |
| **H18** | PT | Community – specialist rehabilitation | 2/2 | Yes | Yes |
| **H19** | OT | Community – generic primary care | 2/2 | Yes | Yes |
| **H20** | SLT | Community intervention team | 2/2 | Yes | Yes |
| **H21** | PT | Community intervention team | 2/2 | Yes | Yes |
| **H22** | CNS | Acute stroke | 1/2 | Yes | No |
| **H23** | Psychologist | Community – generic primary care | 1/2 | Yes | No |
| **H24** | Stroke Consultant | Acute stroke | 1/2 | No | Yes |
| **H25** | SW | Community – generic primary care | 1/2 | Yes | No |
| **H26** | PT | Community – generic primary care | 1/2 | Yes | No |

| **Participant ID** | **Work Setting** | **Number of workshops** | | **Workshop 1** | **Workshop 3** |
| --- | --- | --- | --- | --- | --- |
| **S1** | National Learning Network | | 2/2 | Yes | Yes |
| **S2** | Acquired Brain Injury Ireland | | 2/2 | Yes | Yes |
| **S3** | Day Service | | 1/2 | No | Yes |
| **S4** | Irish Heart Foundation | | 1/2 | Yes | No |
| **S5** | Irish Heart Foundation | | 1/2 | Yes | No |
| **S6** | Day Service | | 1/2 | Yes | No |

**Supplemental material II:** Workshop slides including activities and tasks

**
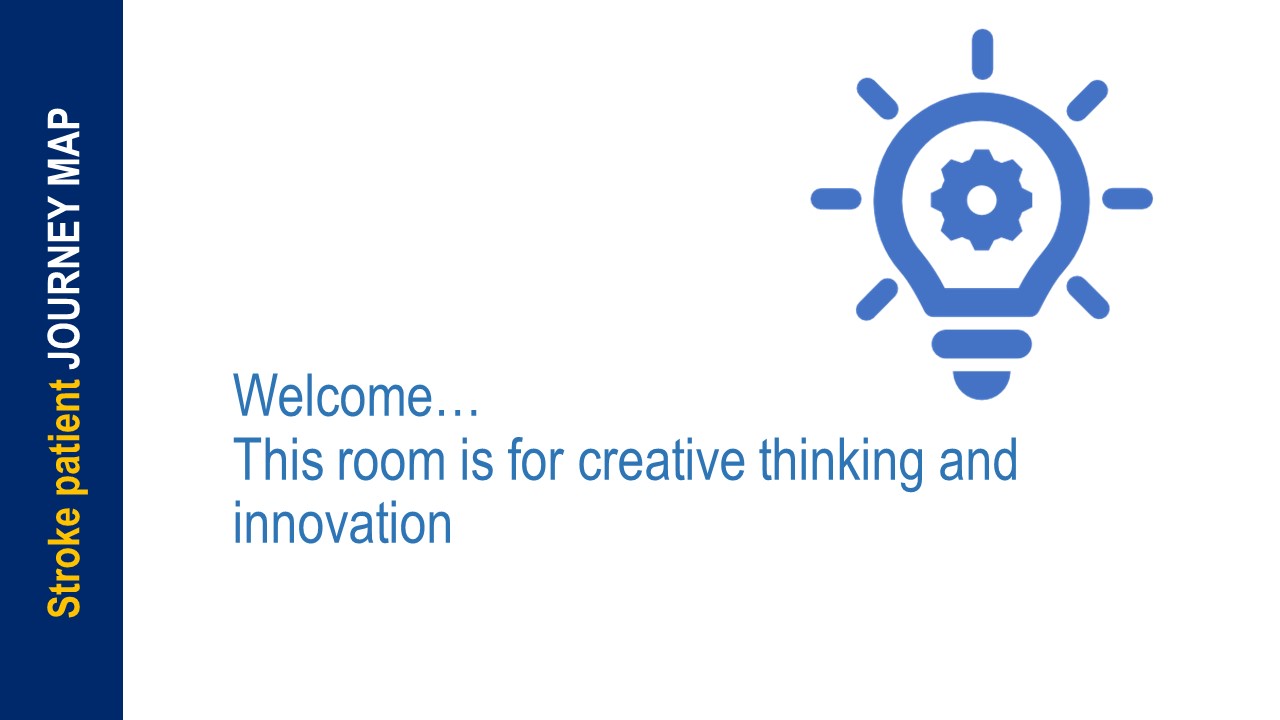
**

**
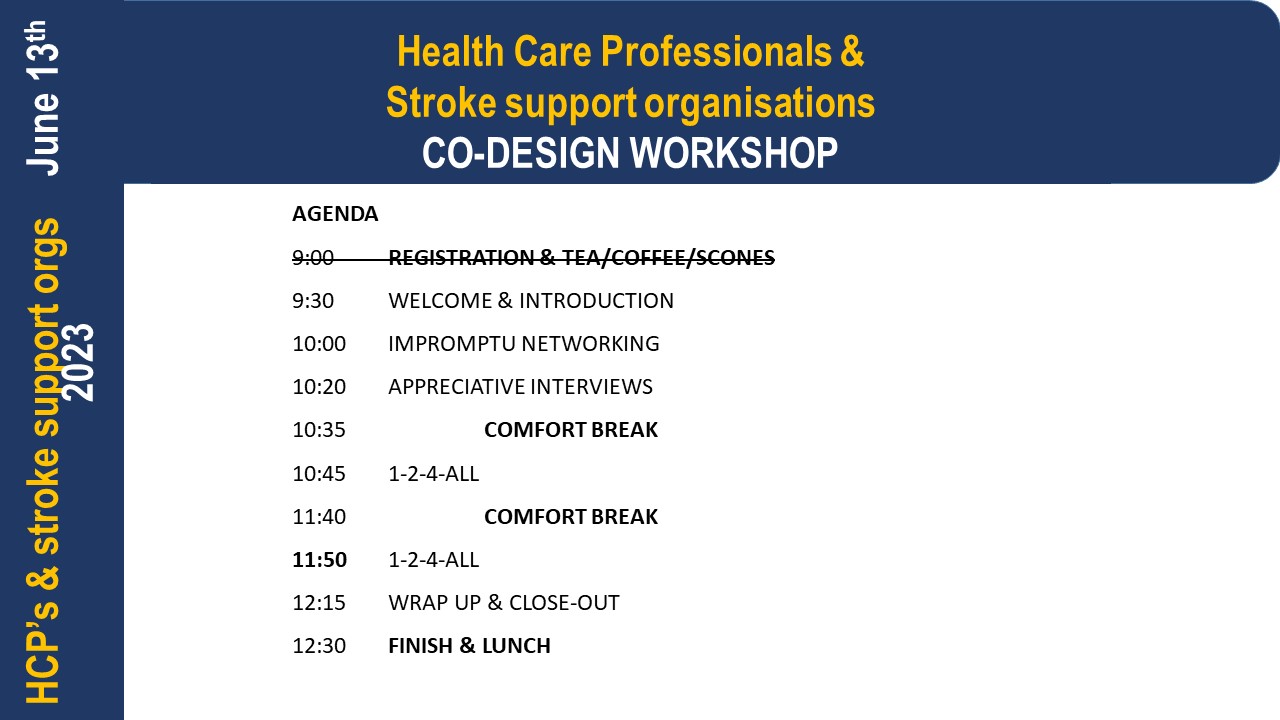

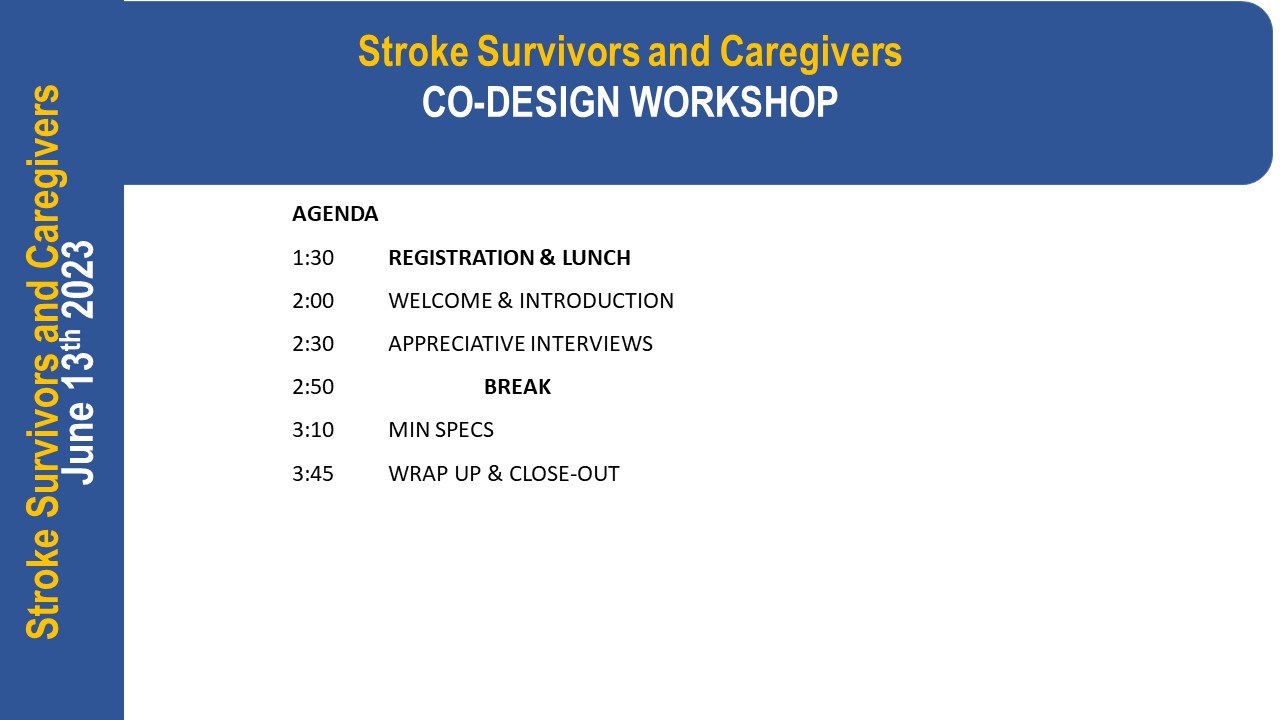

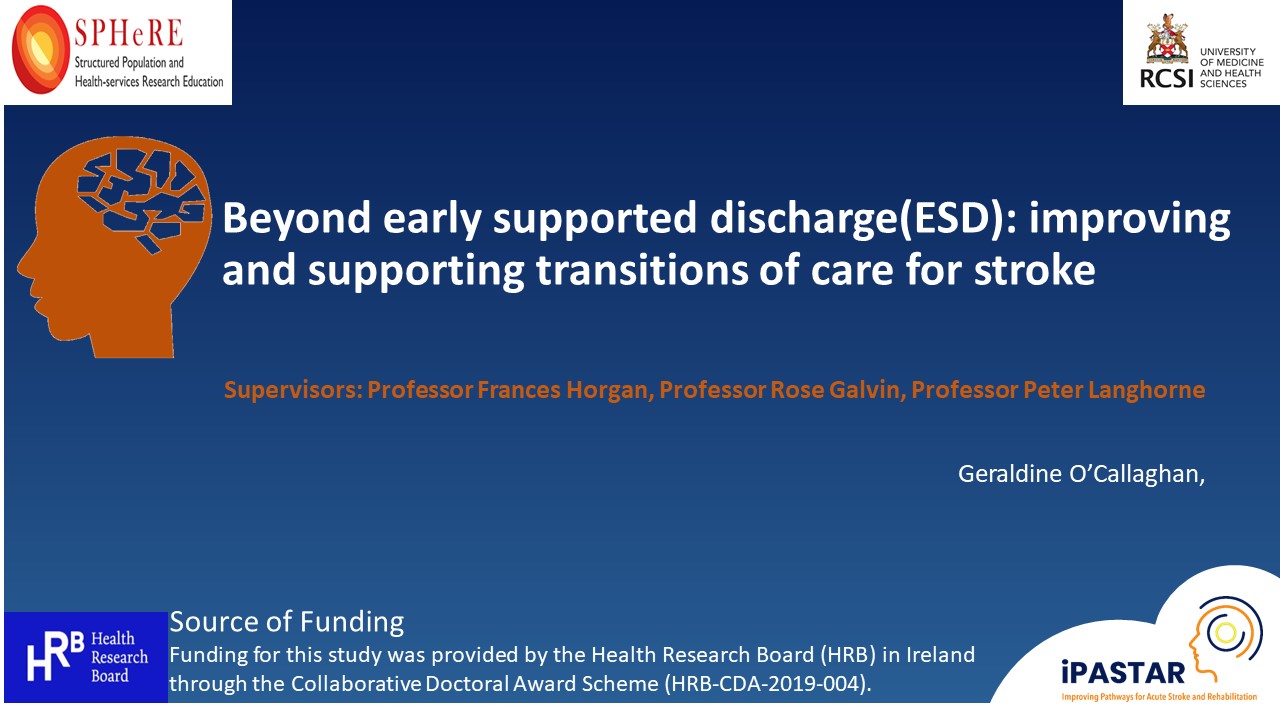

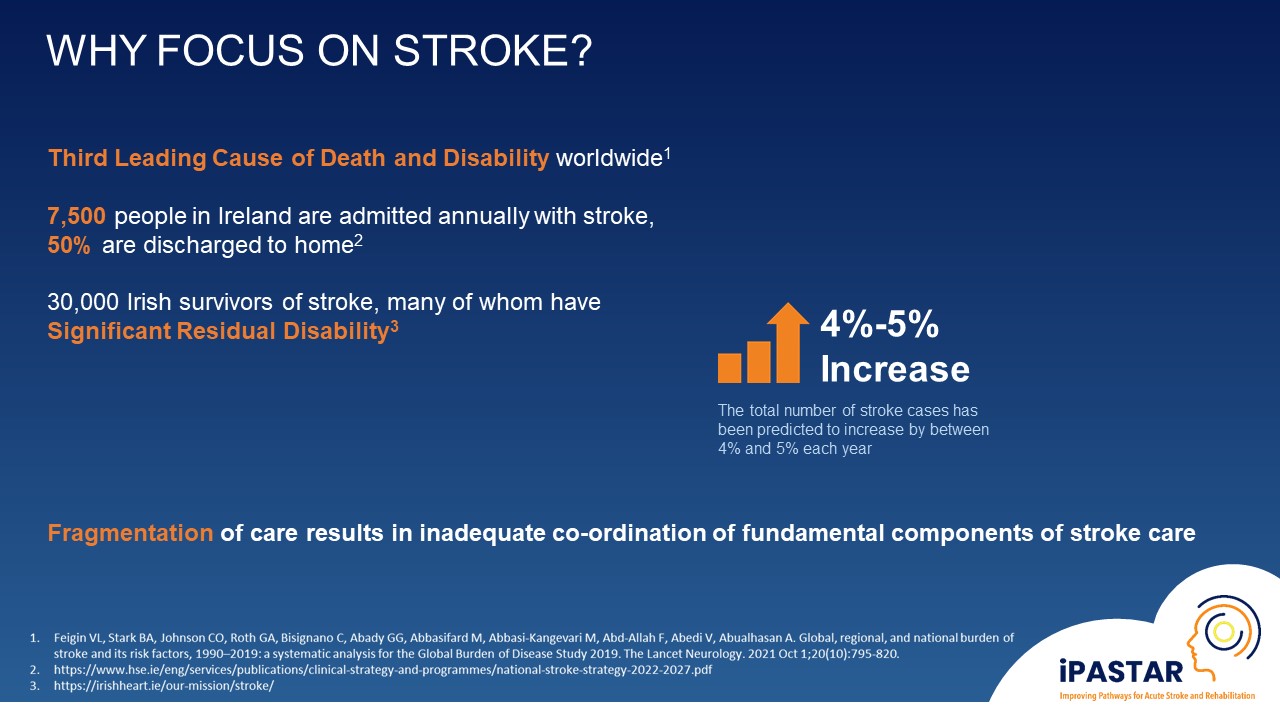

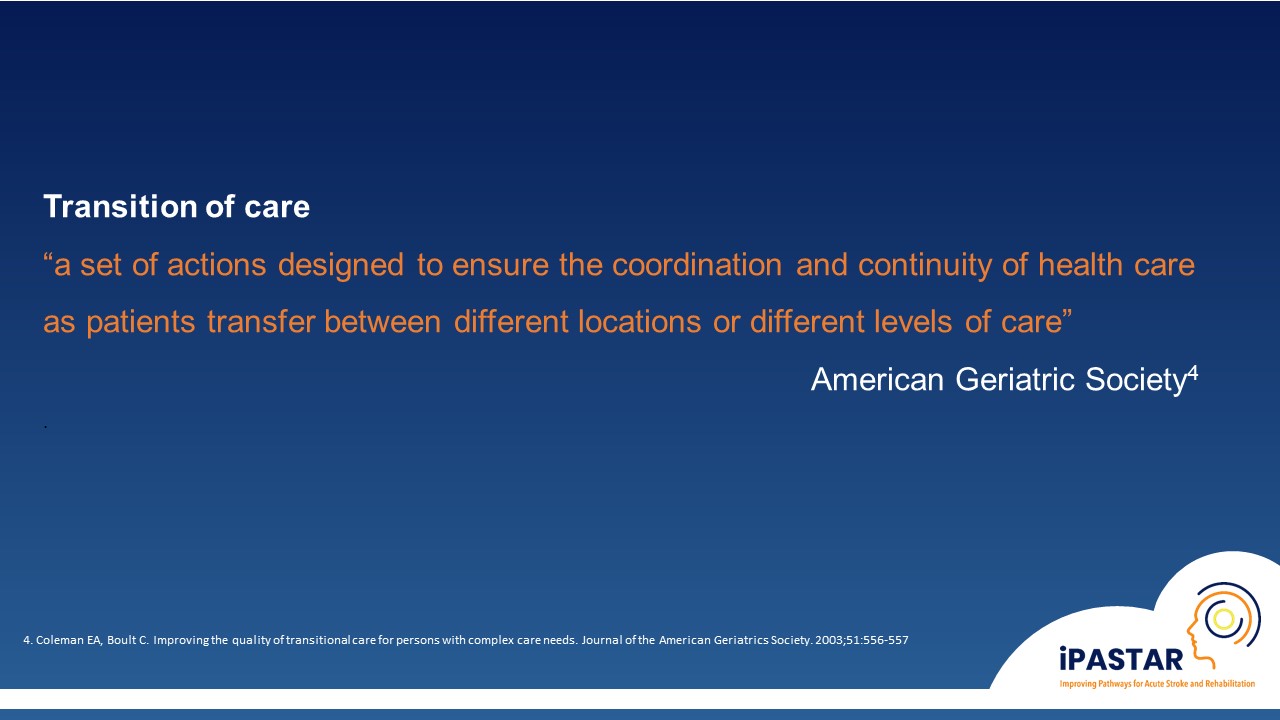

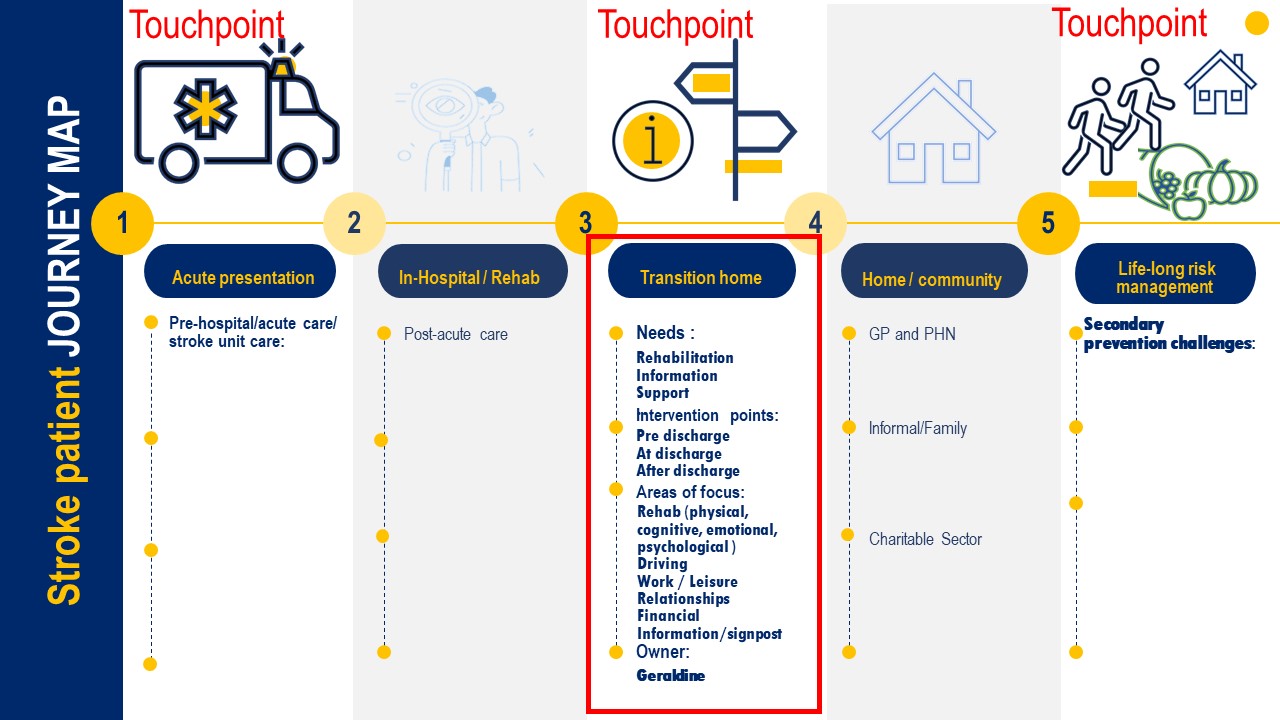

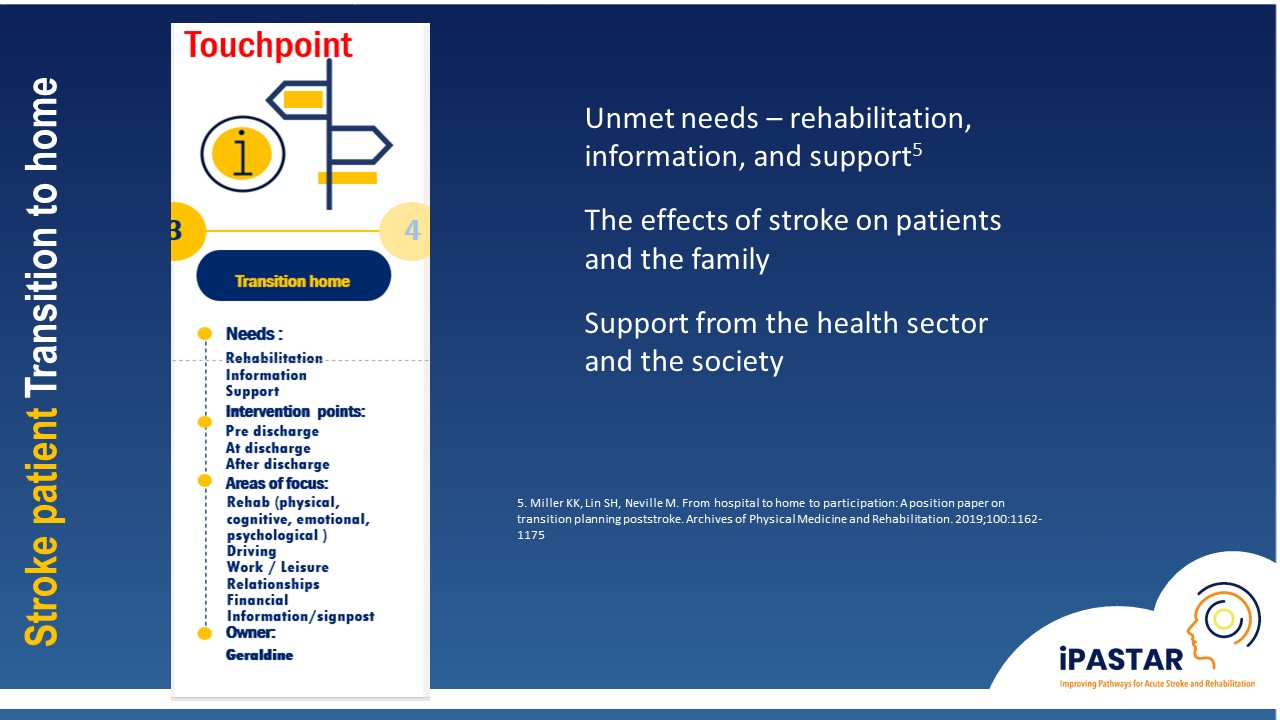

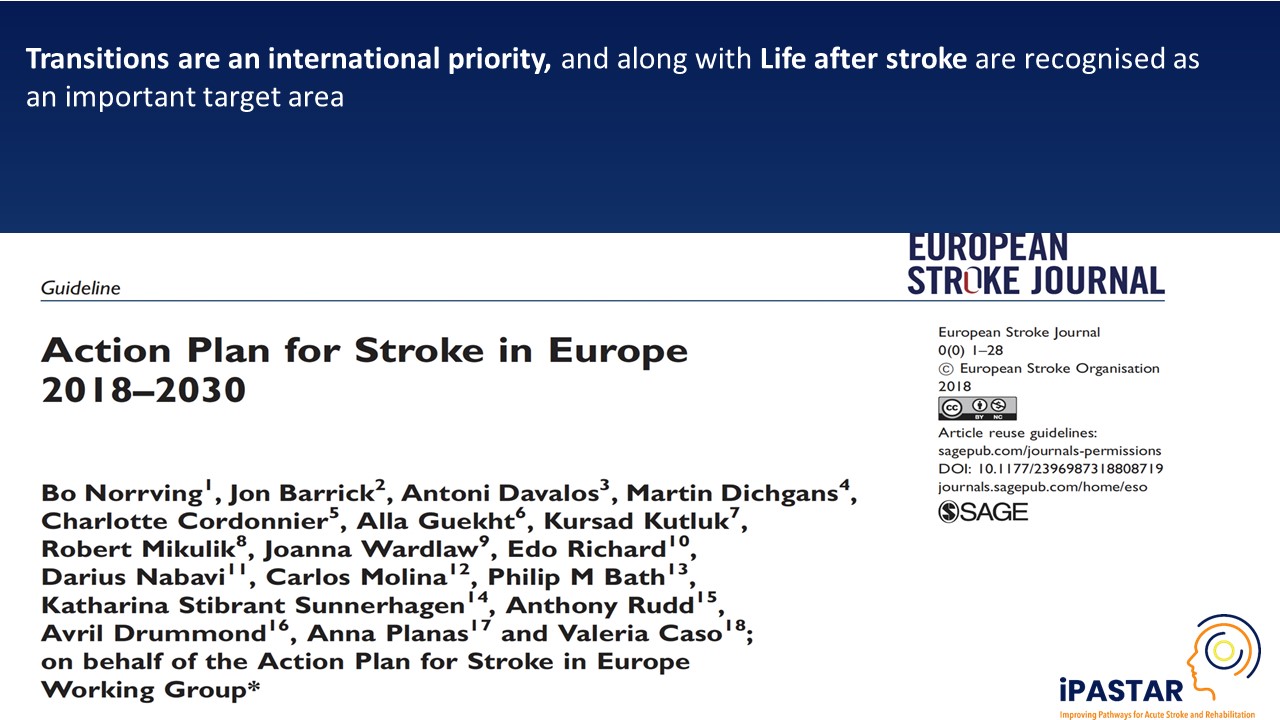

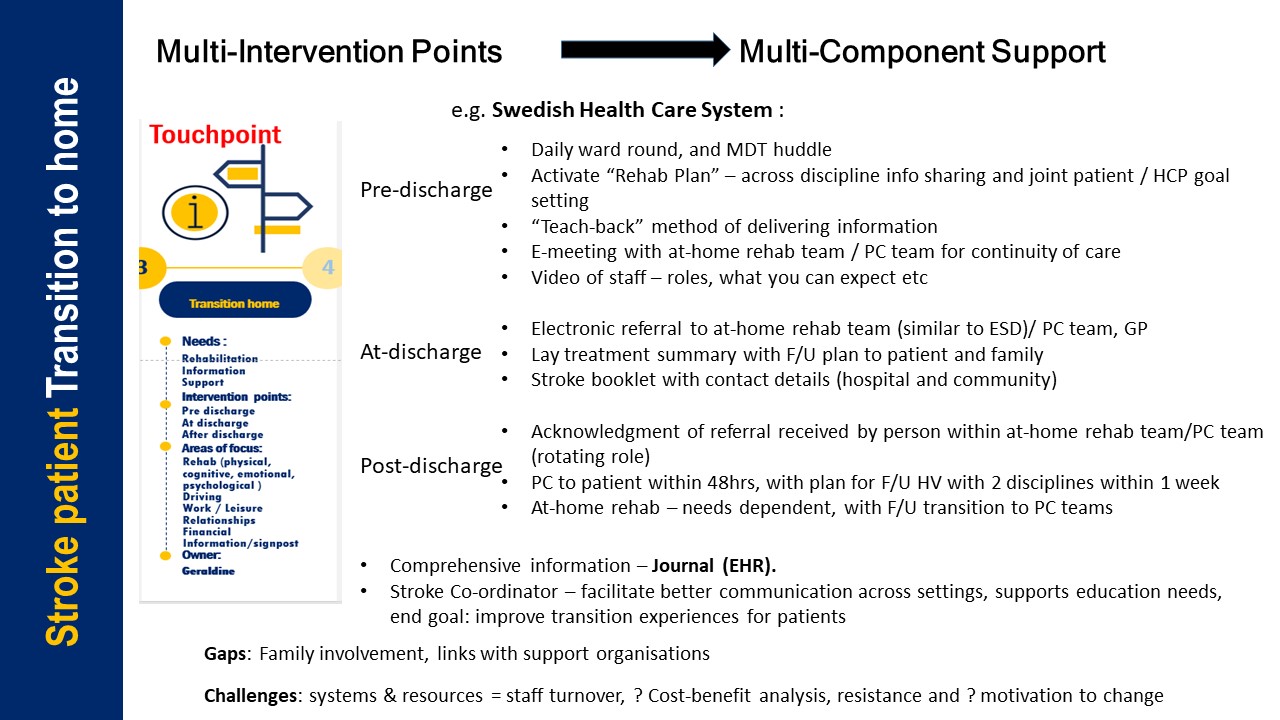

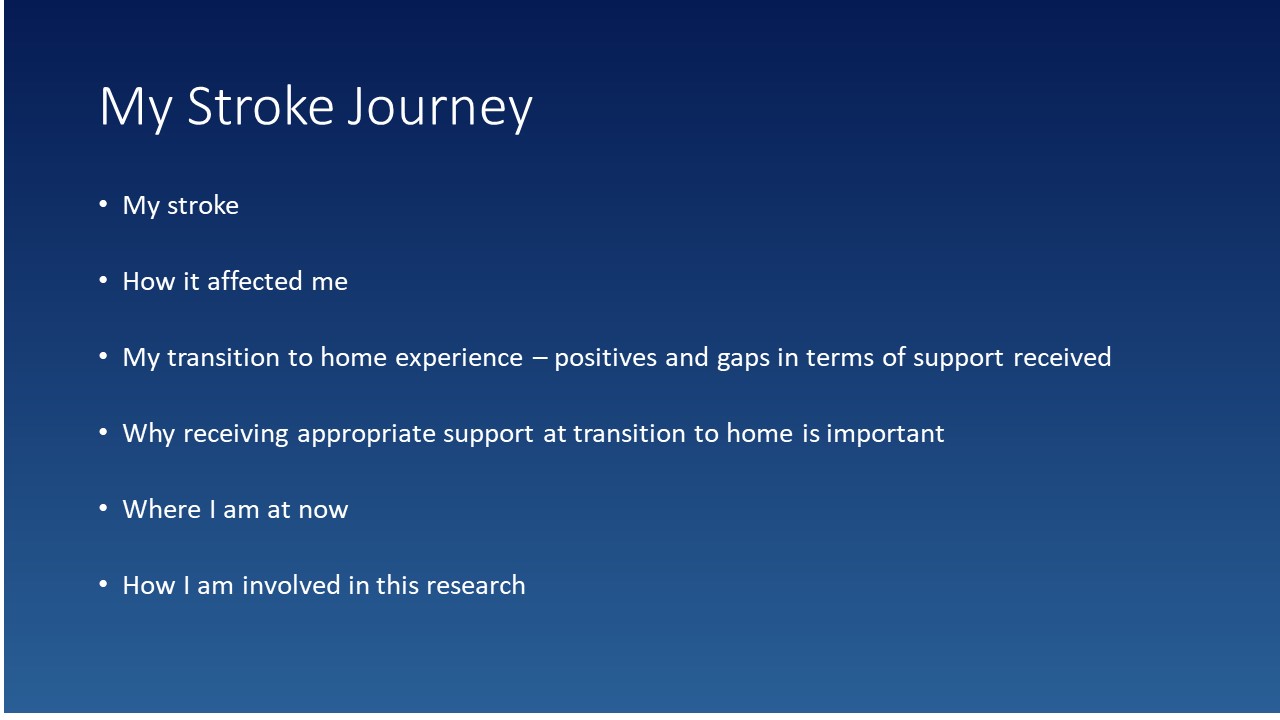

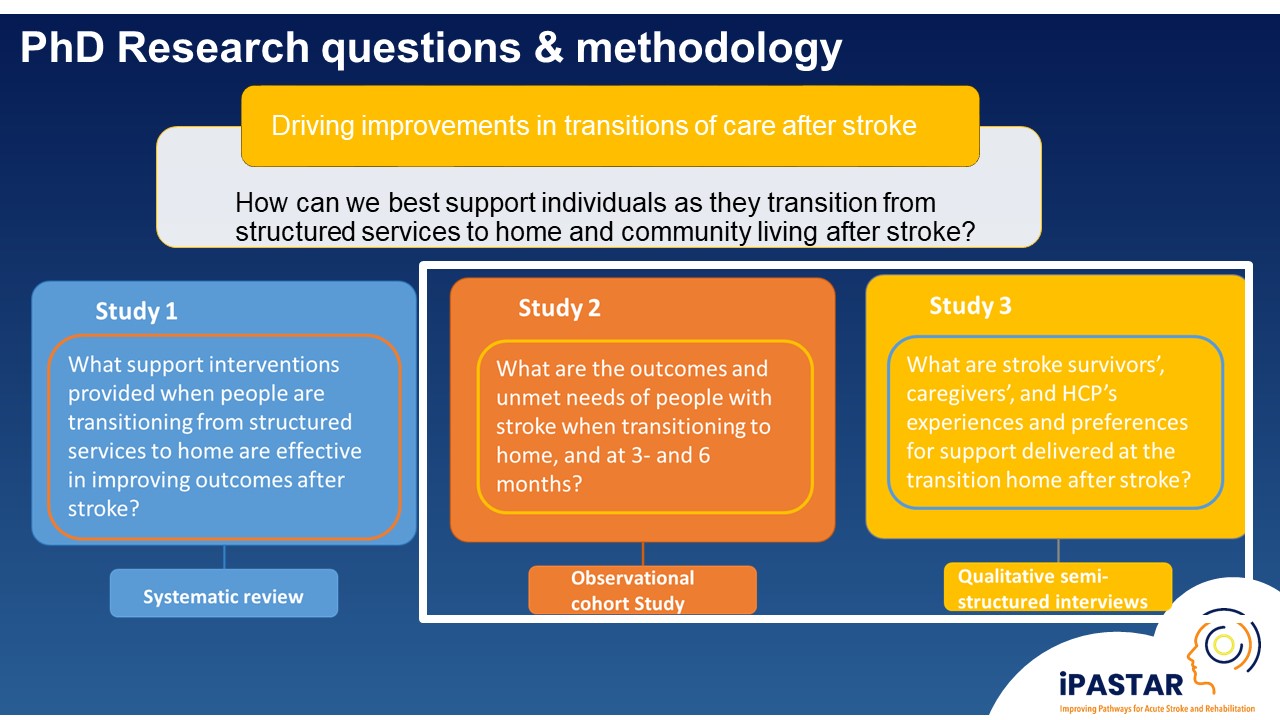

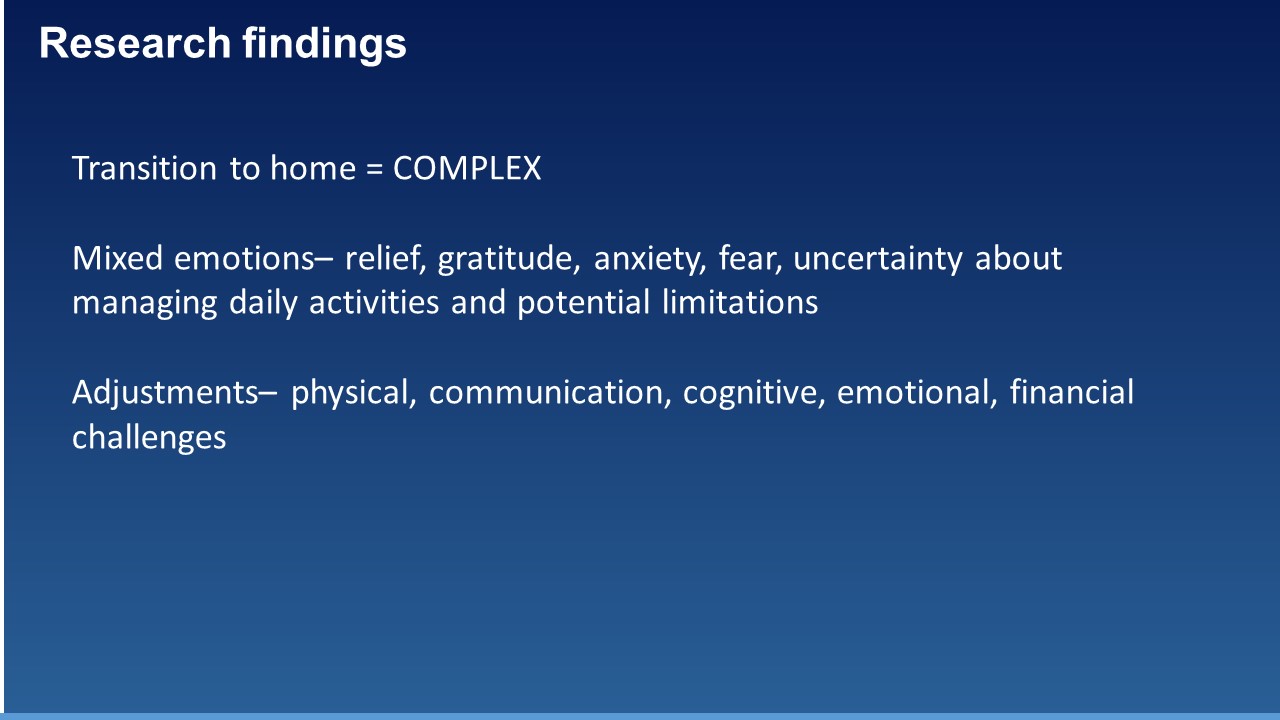

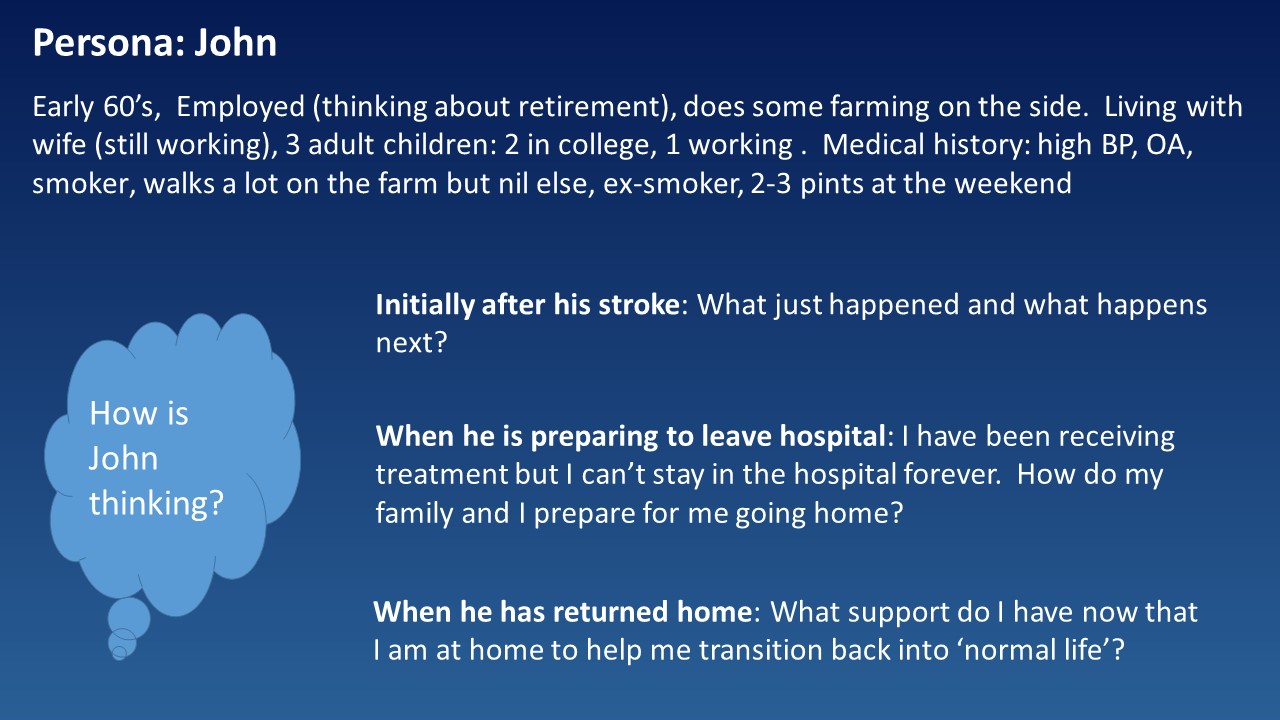

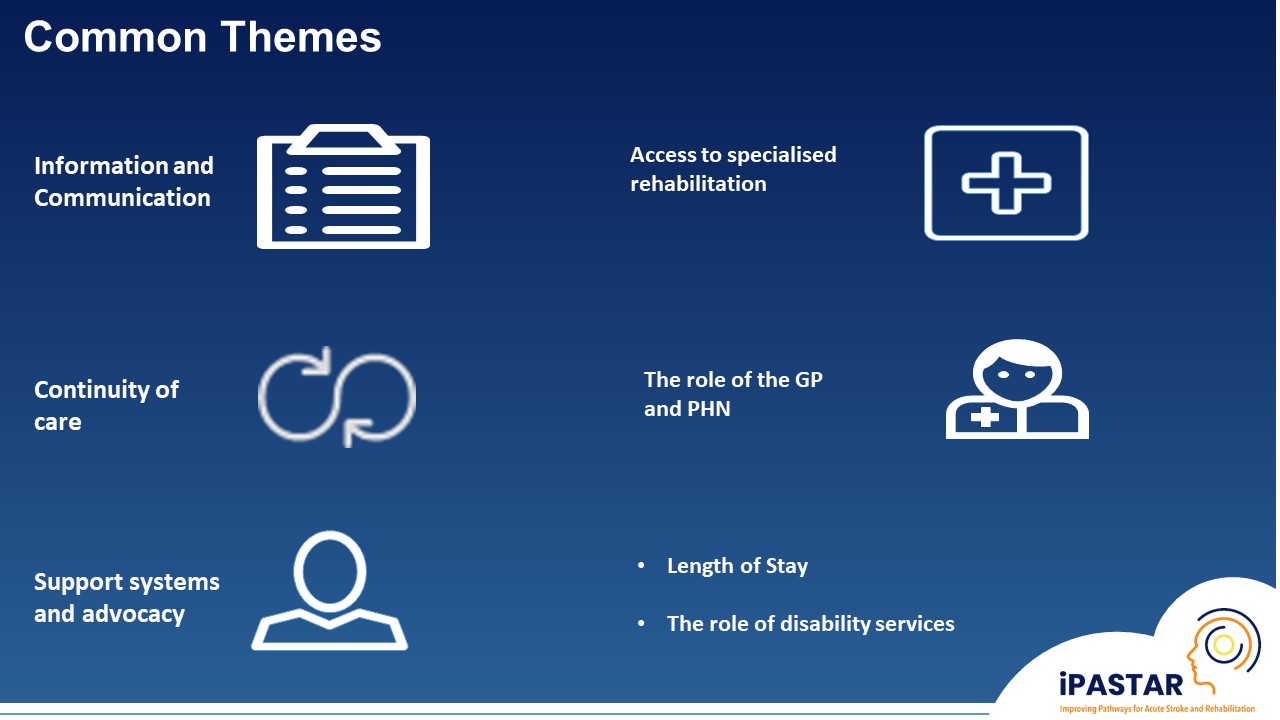

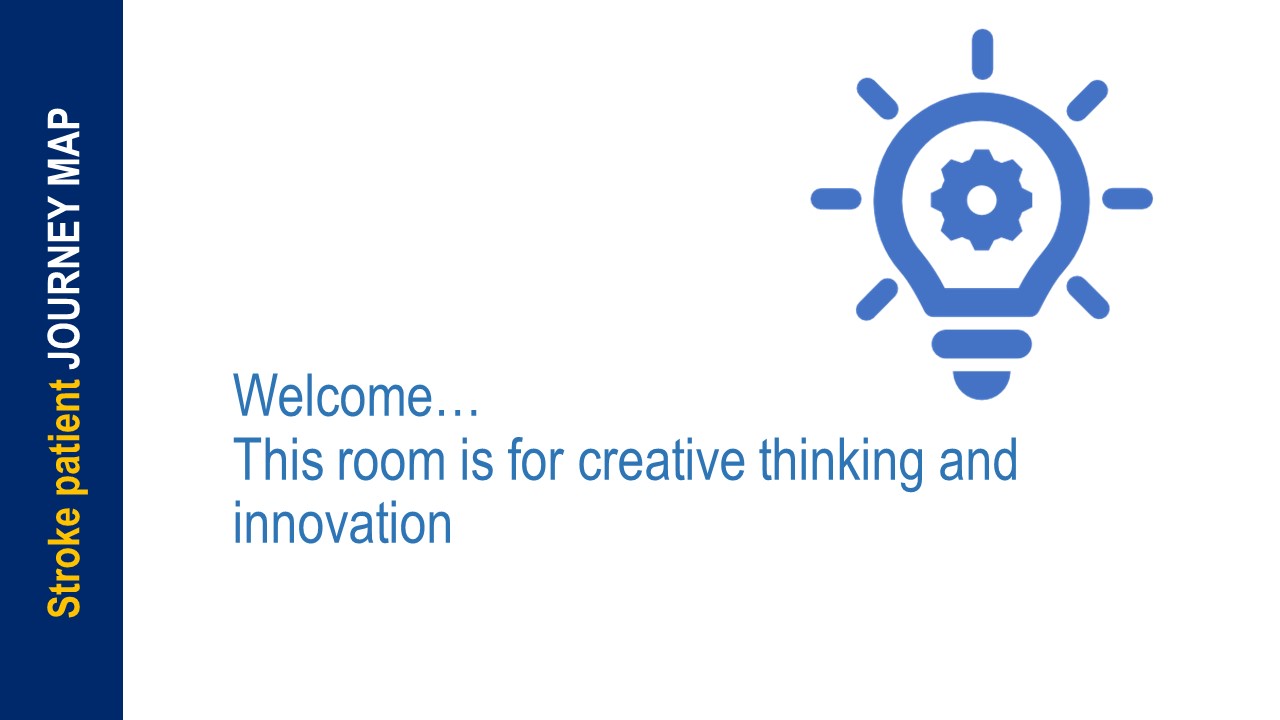

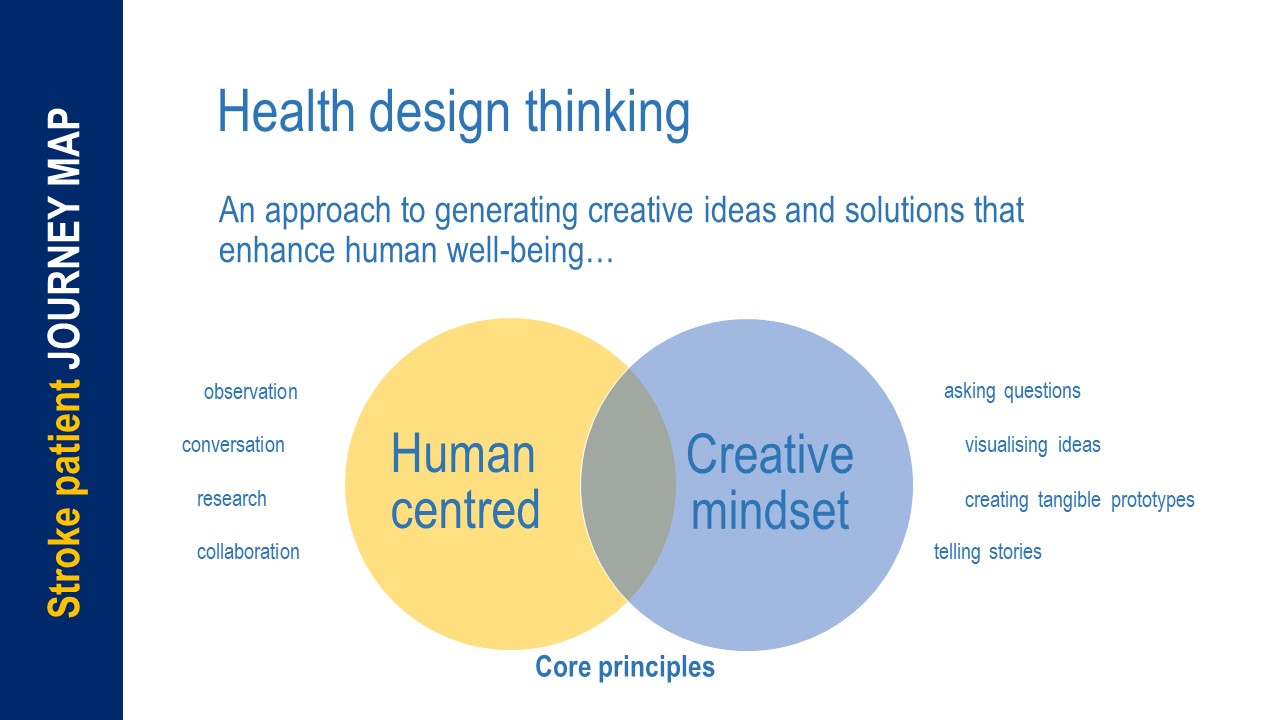

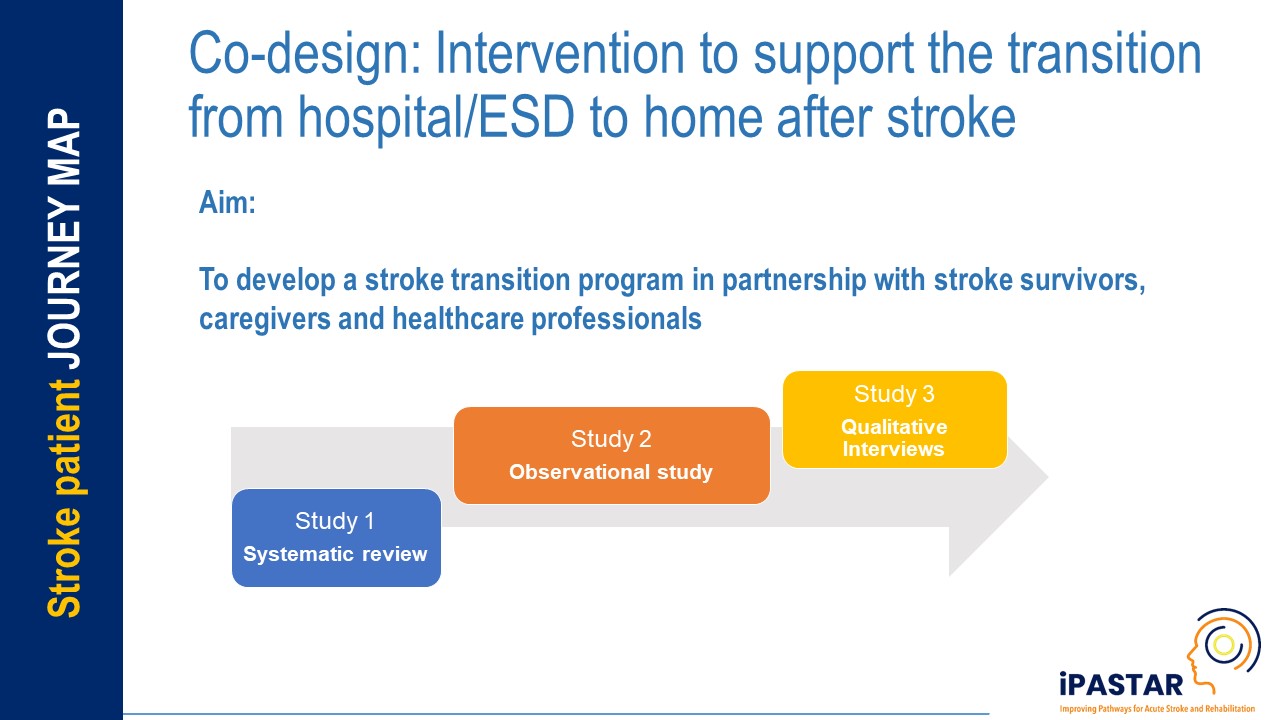

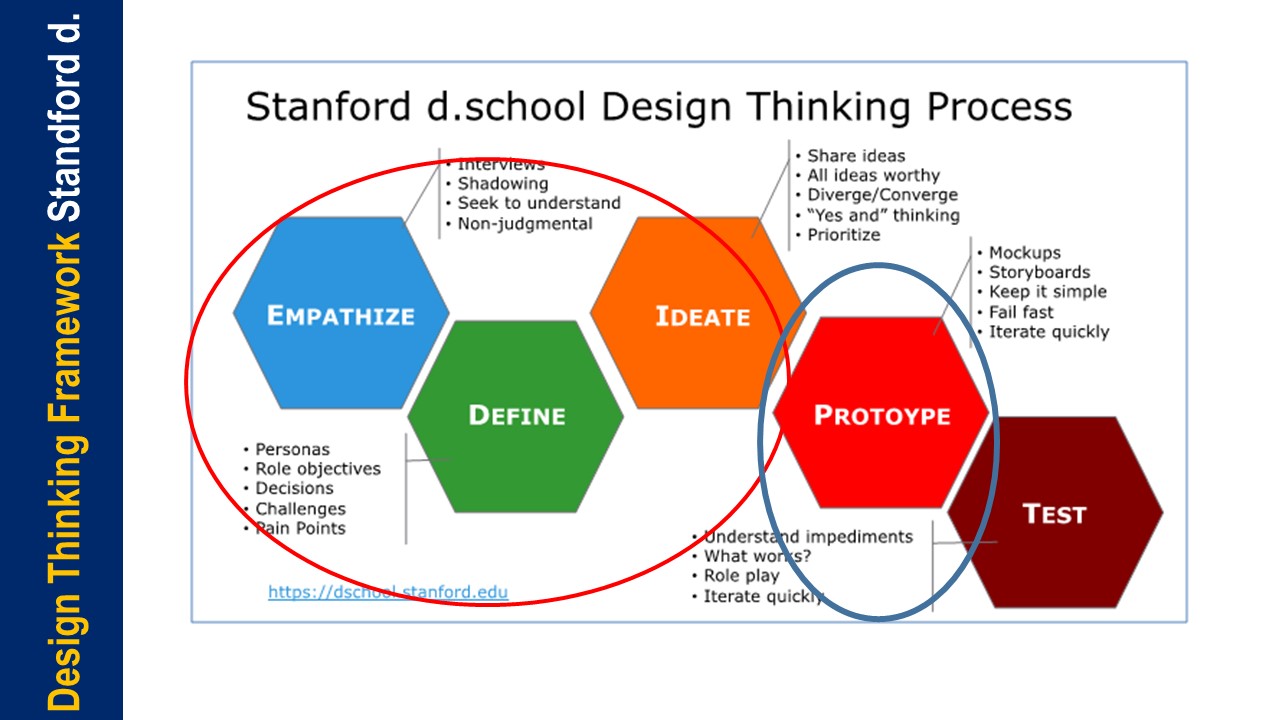

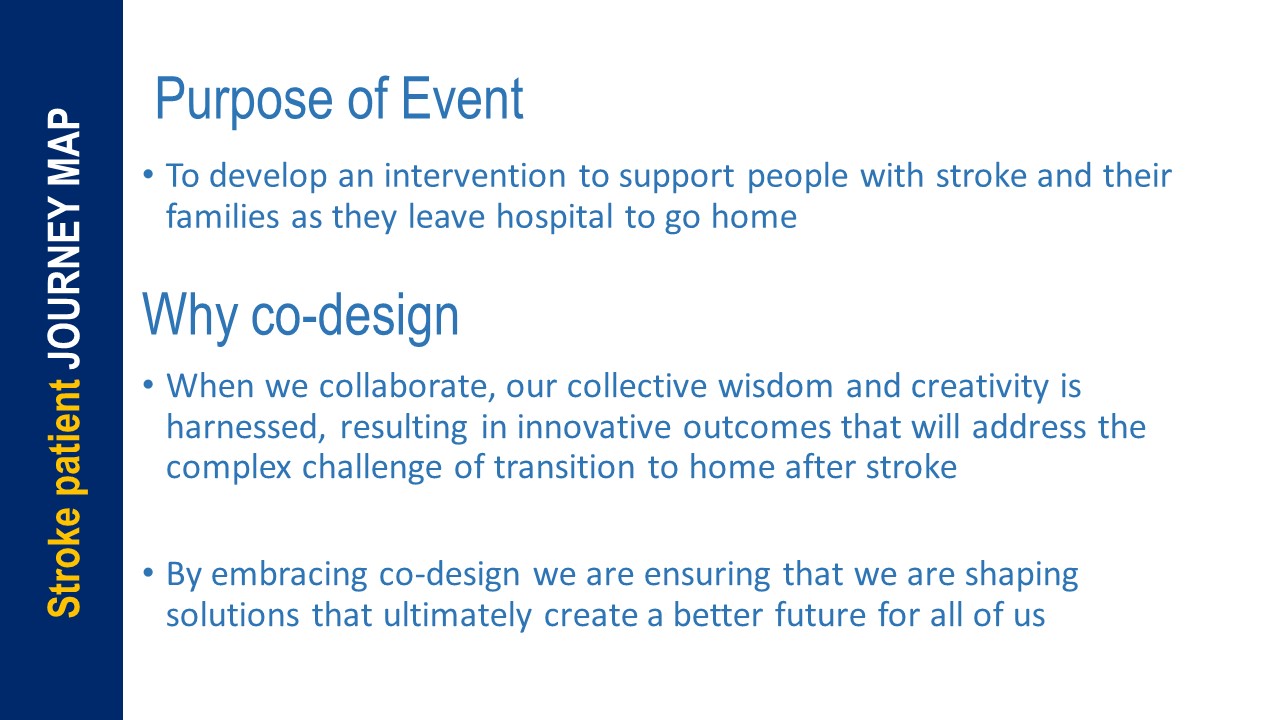

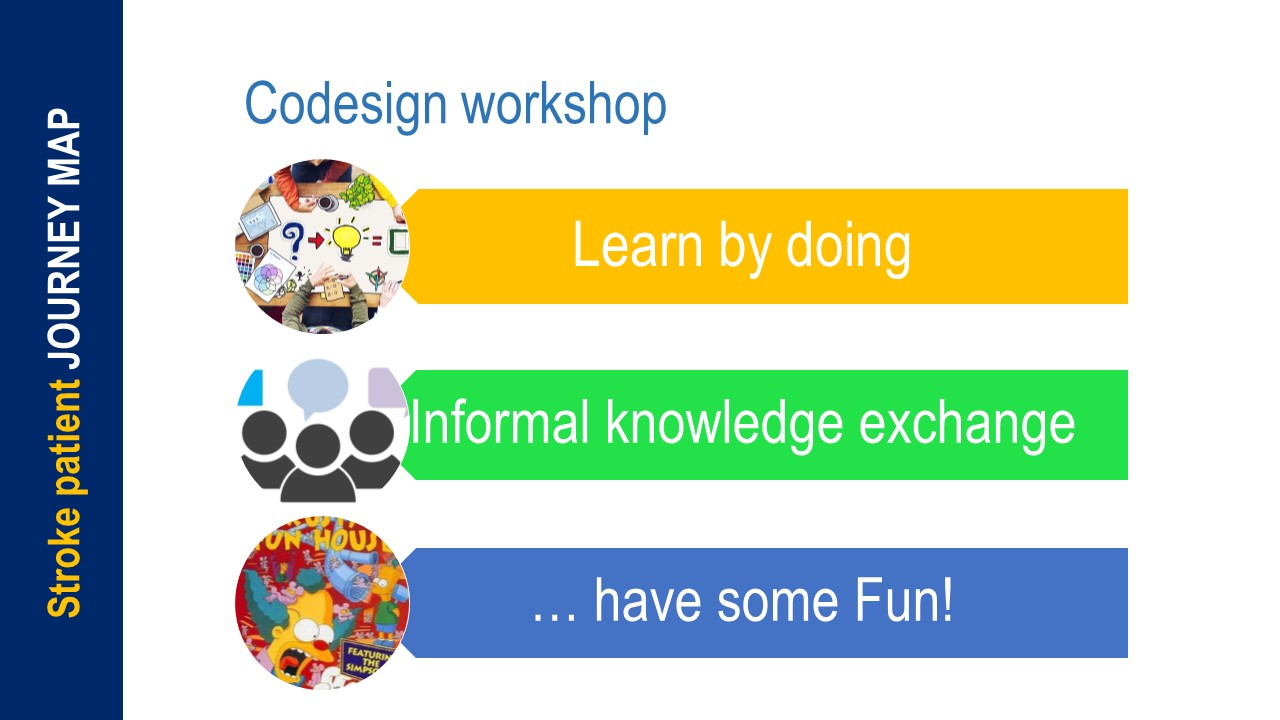

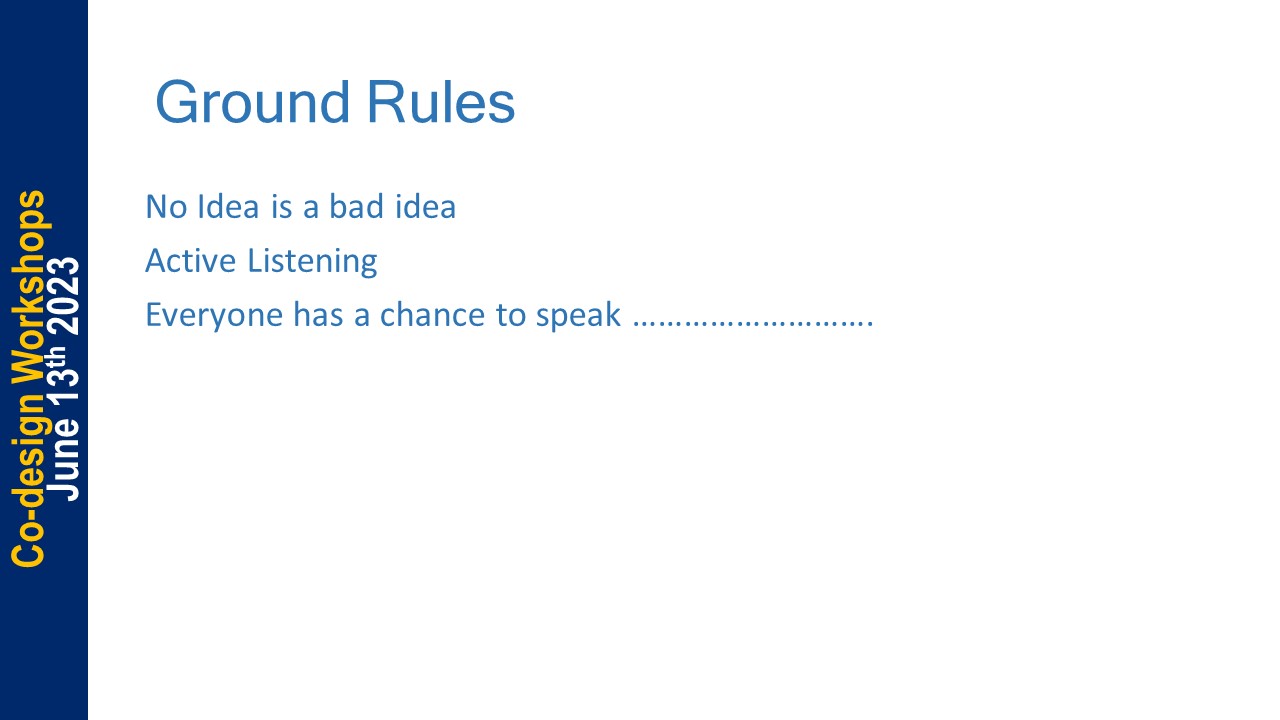

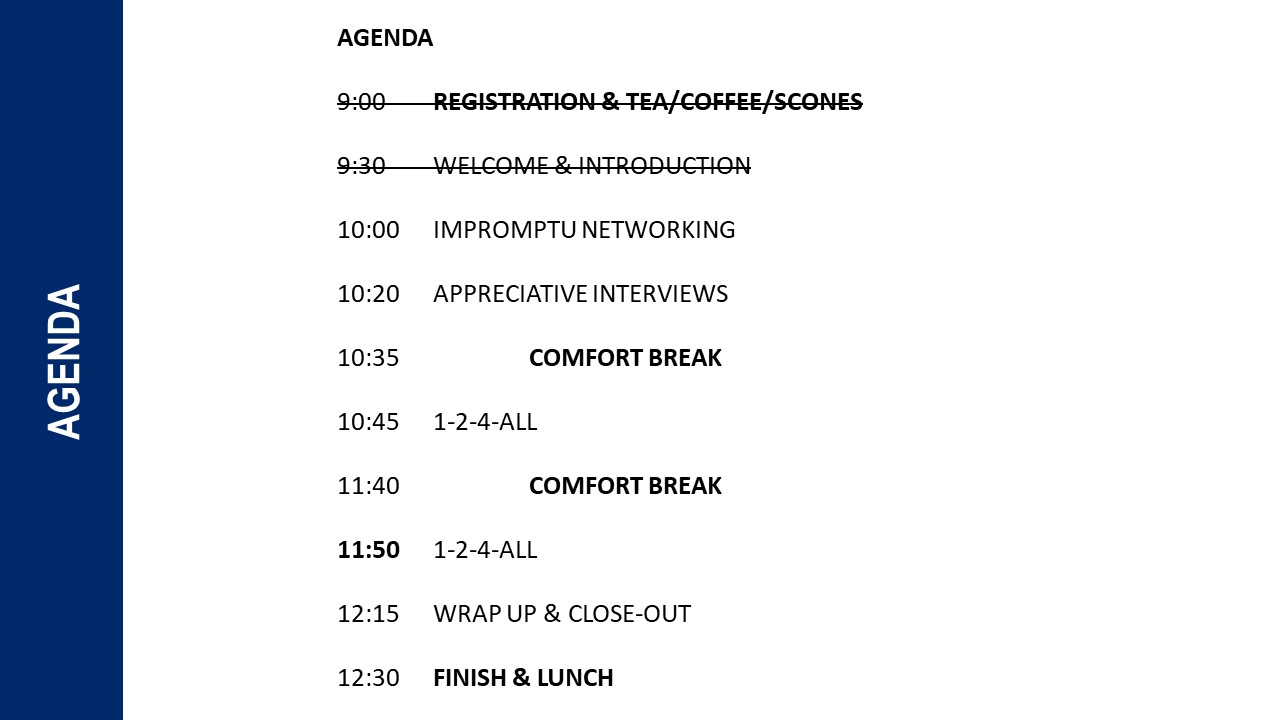

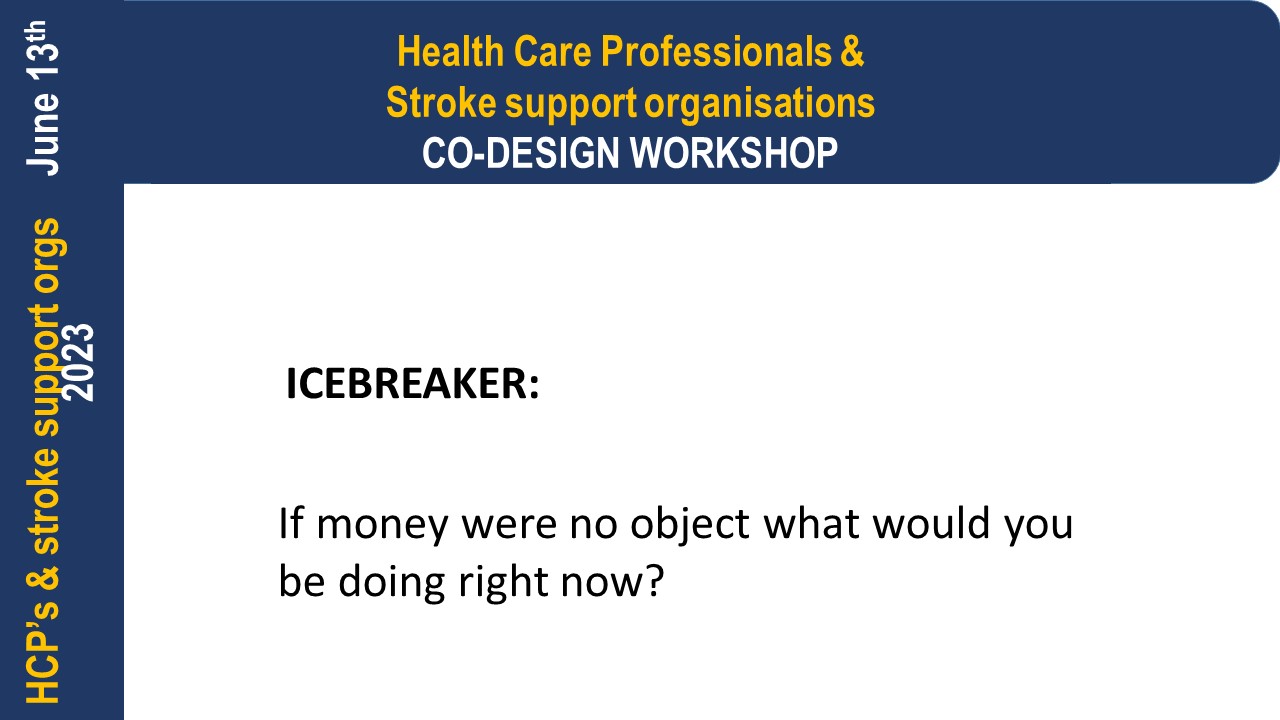

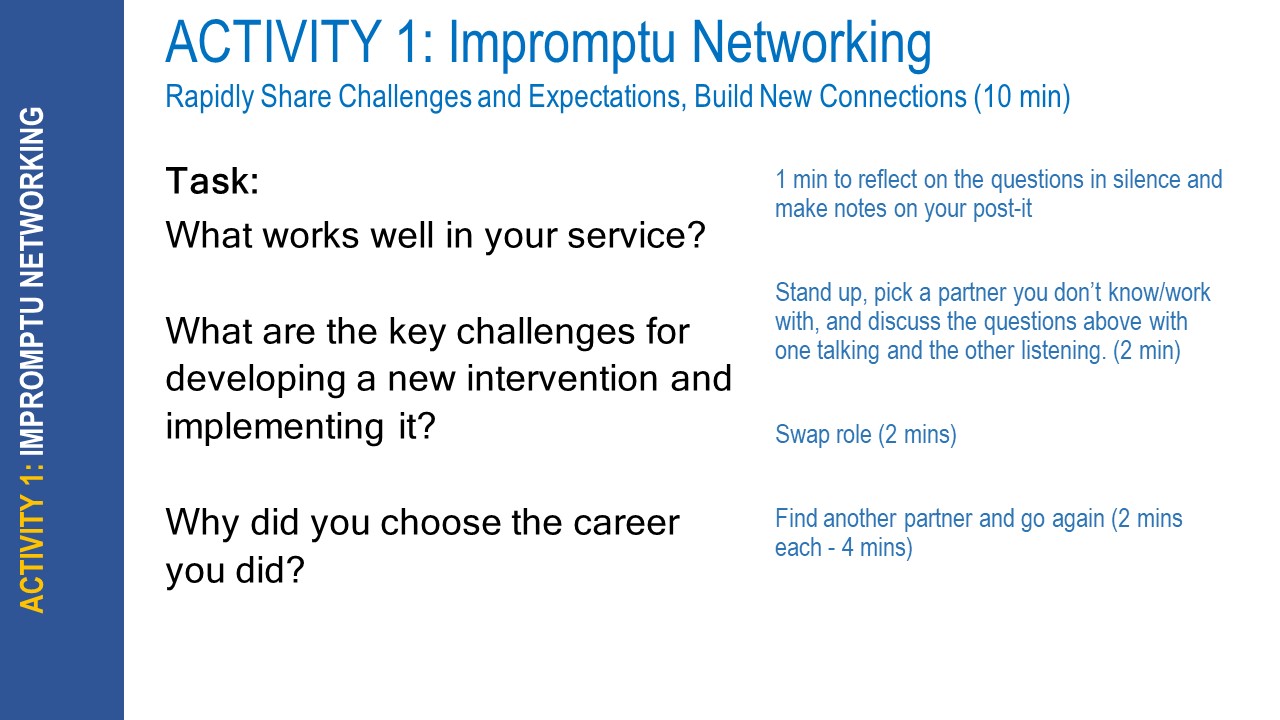

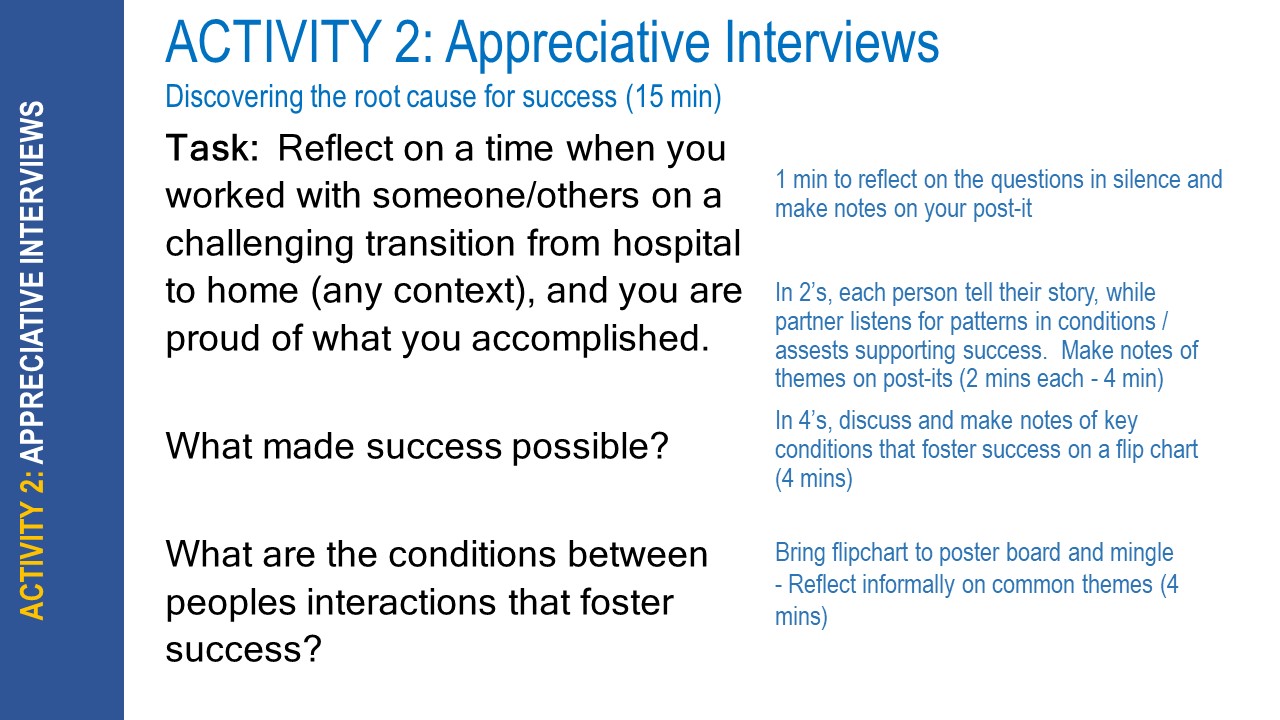

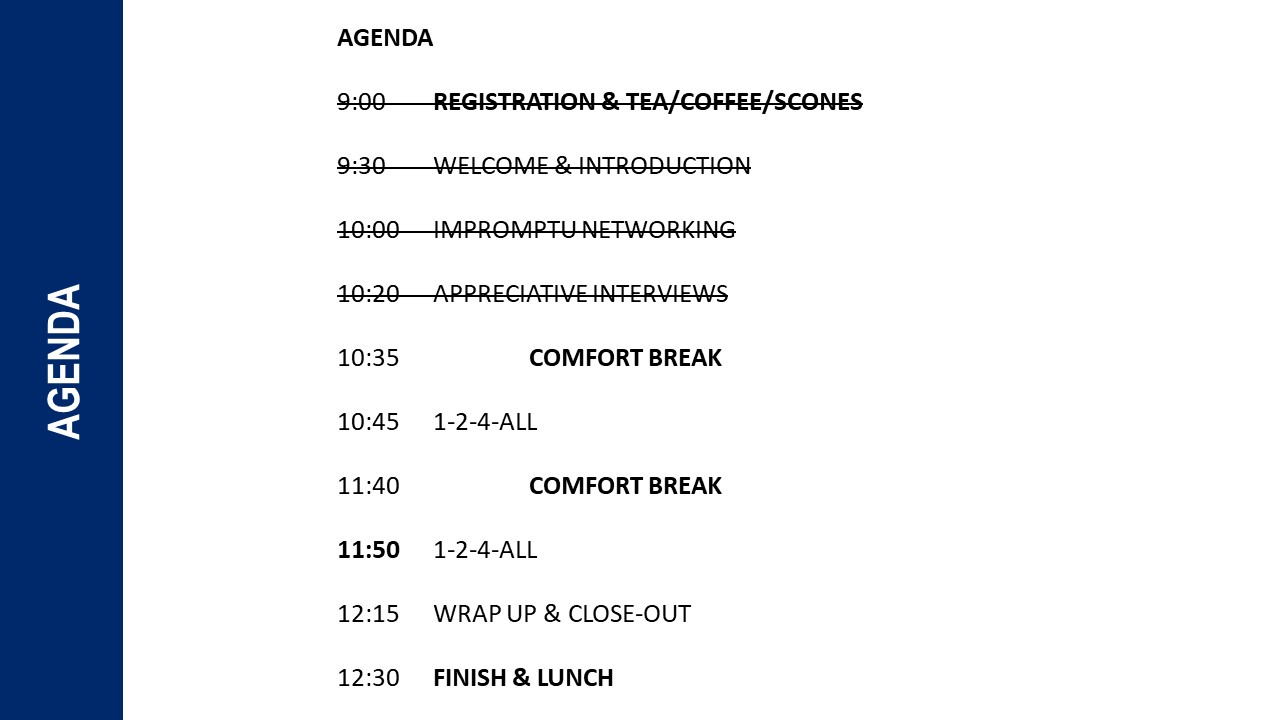

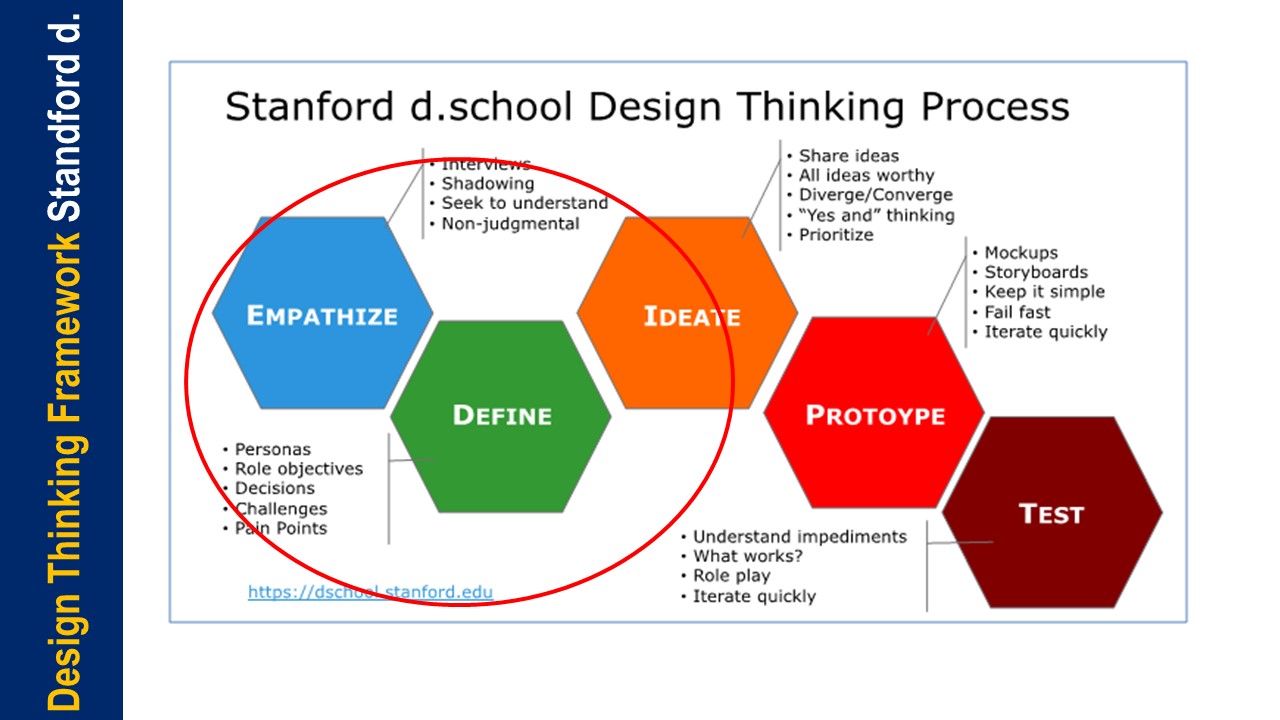

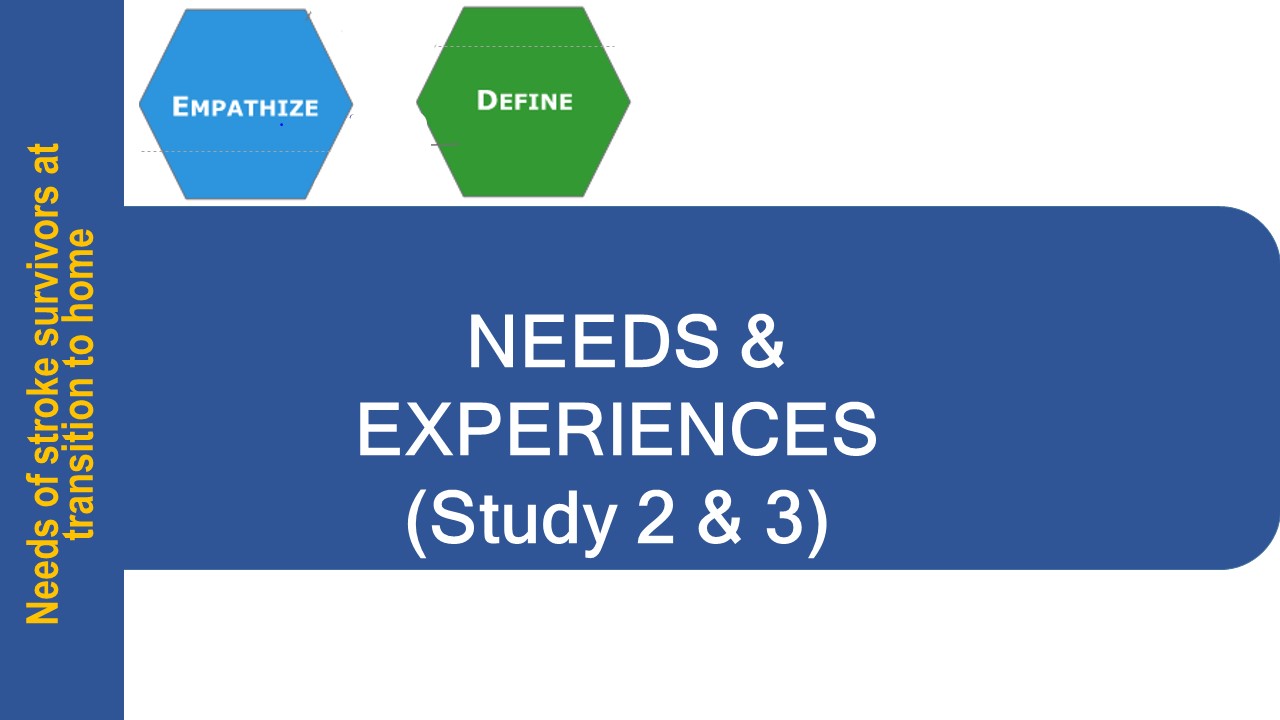

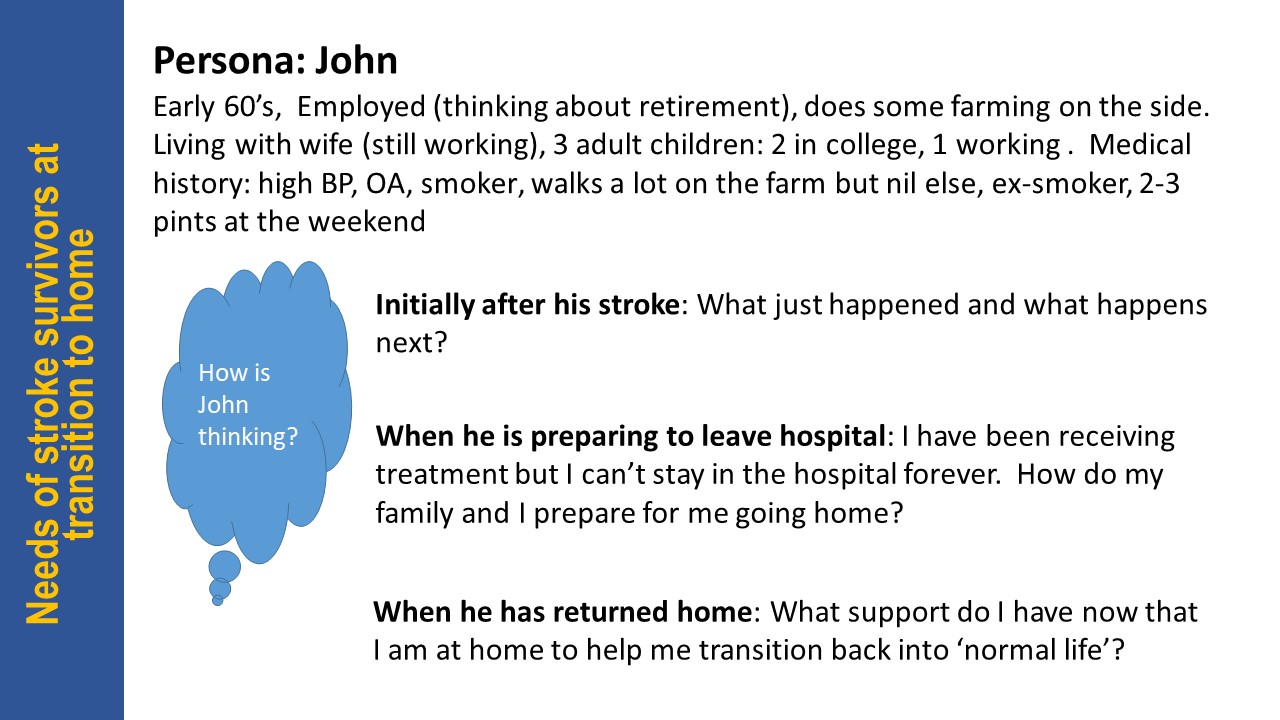

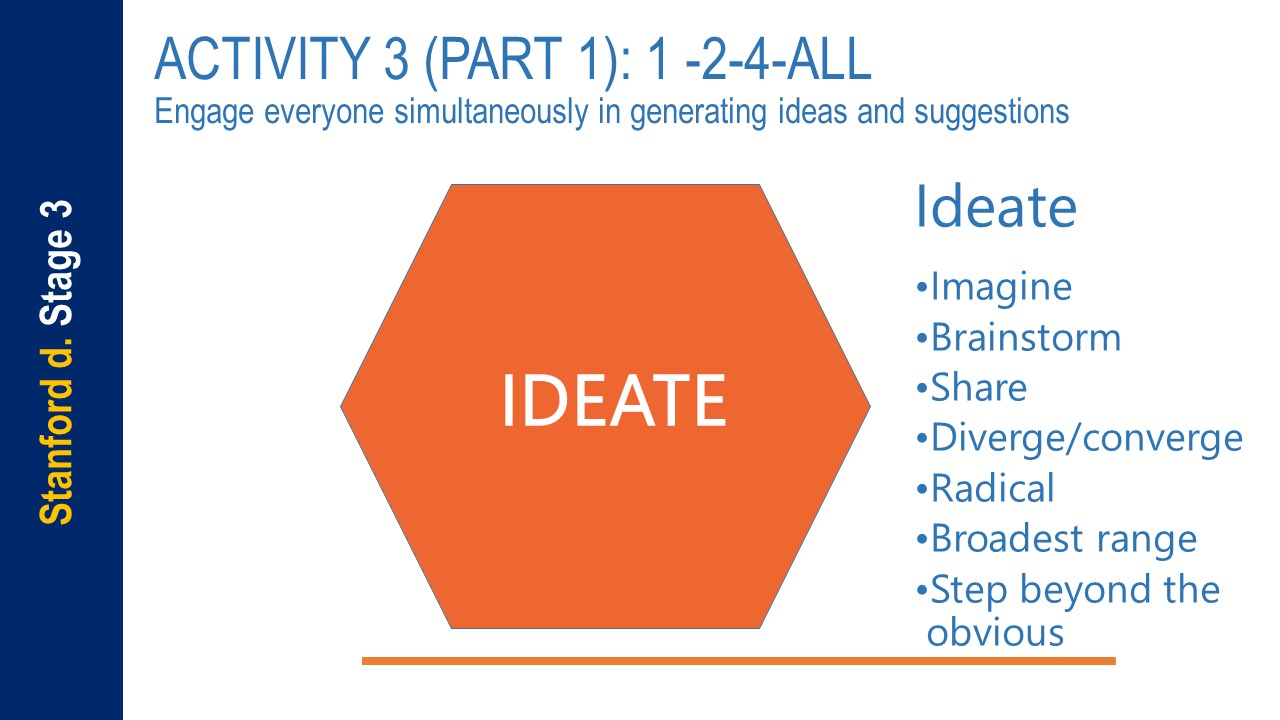

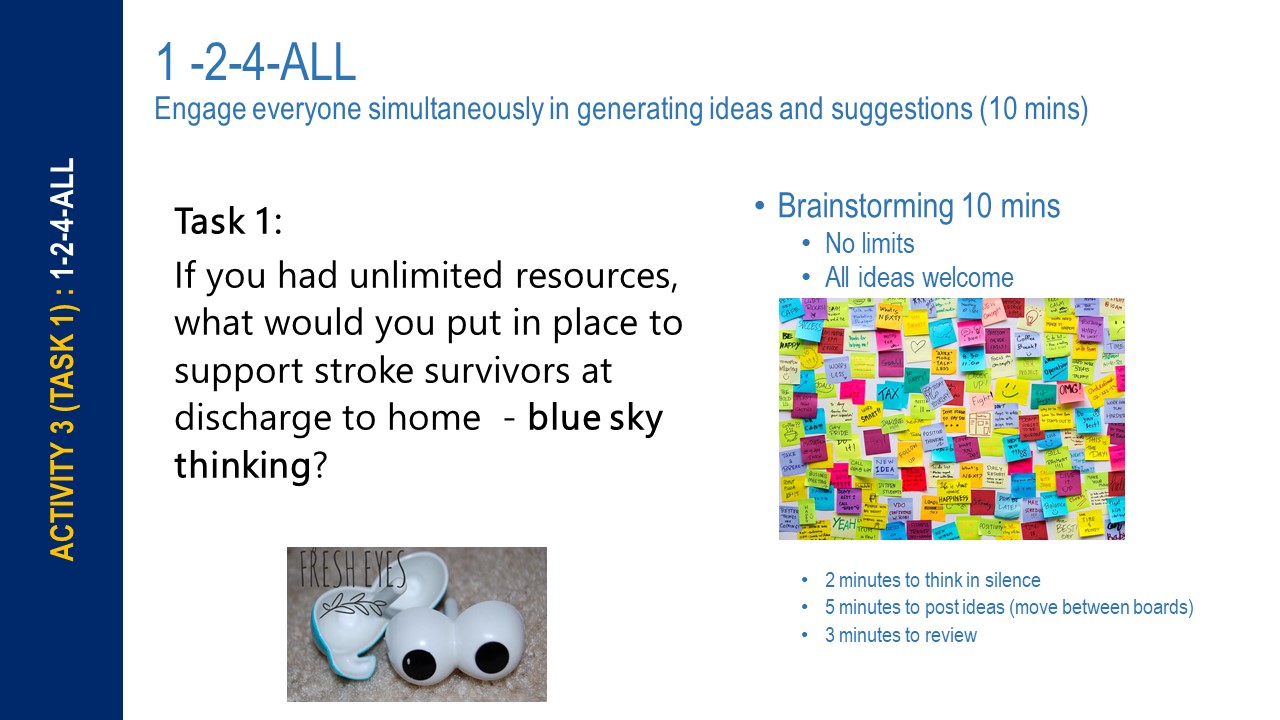

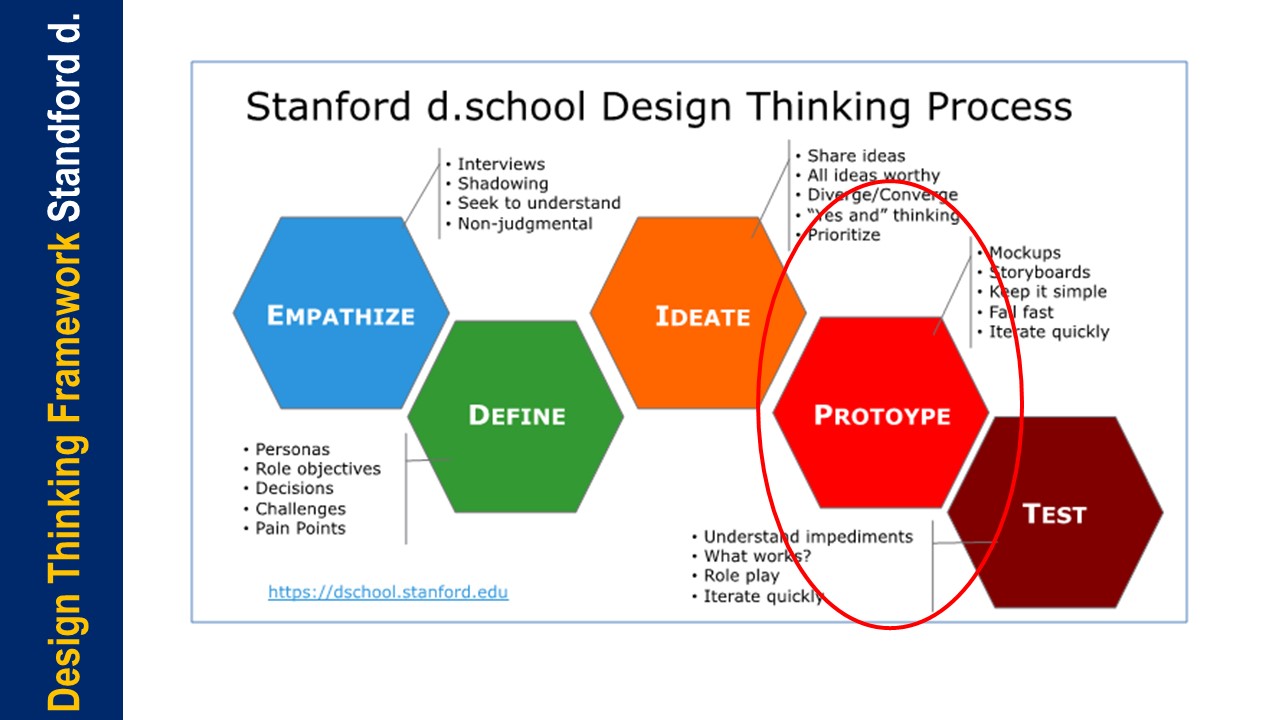

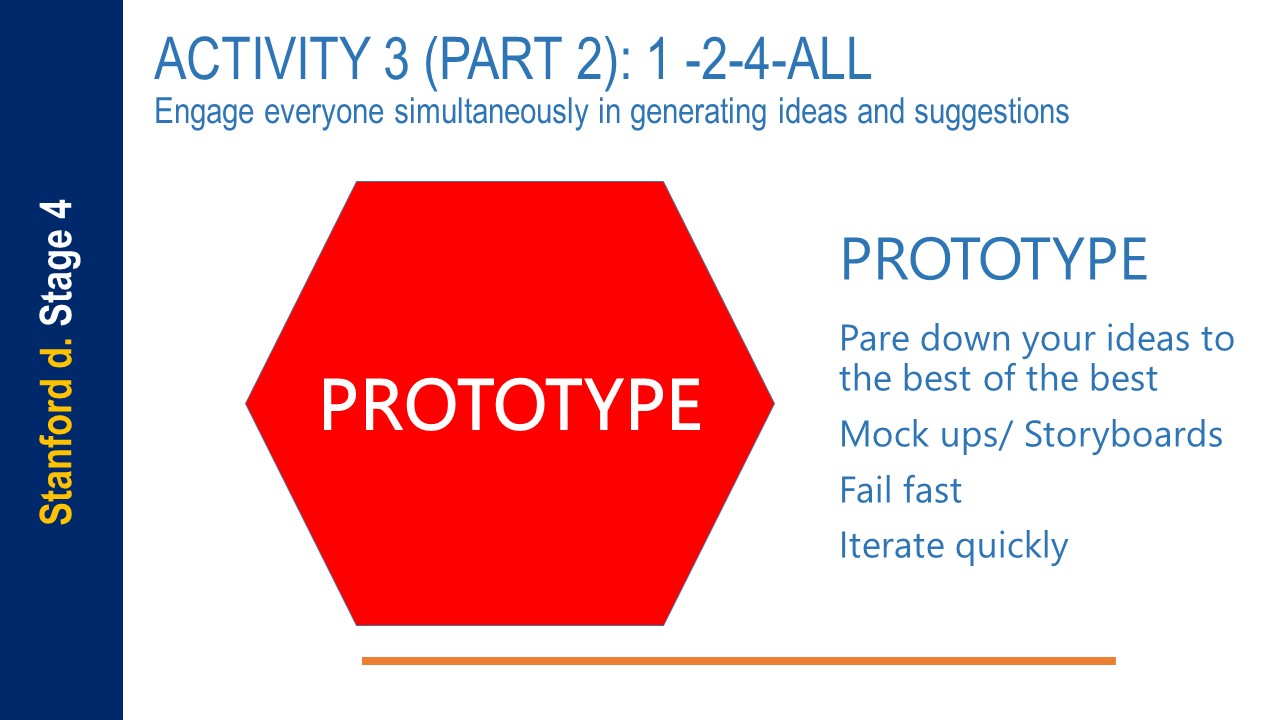

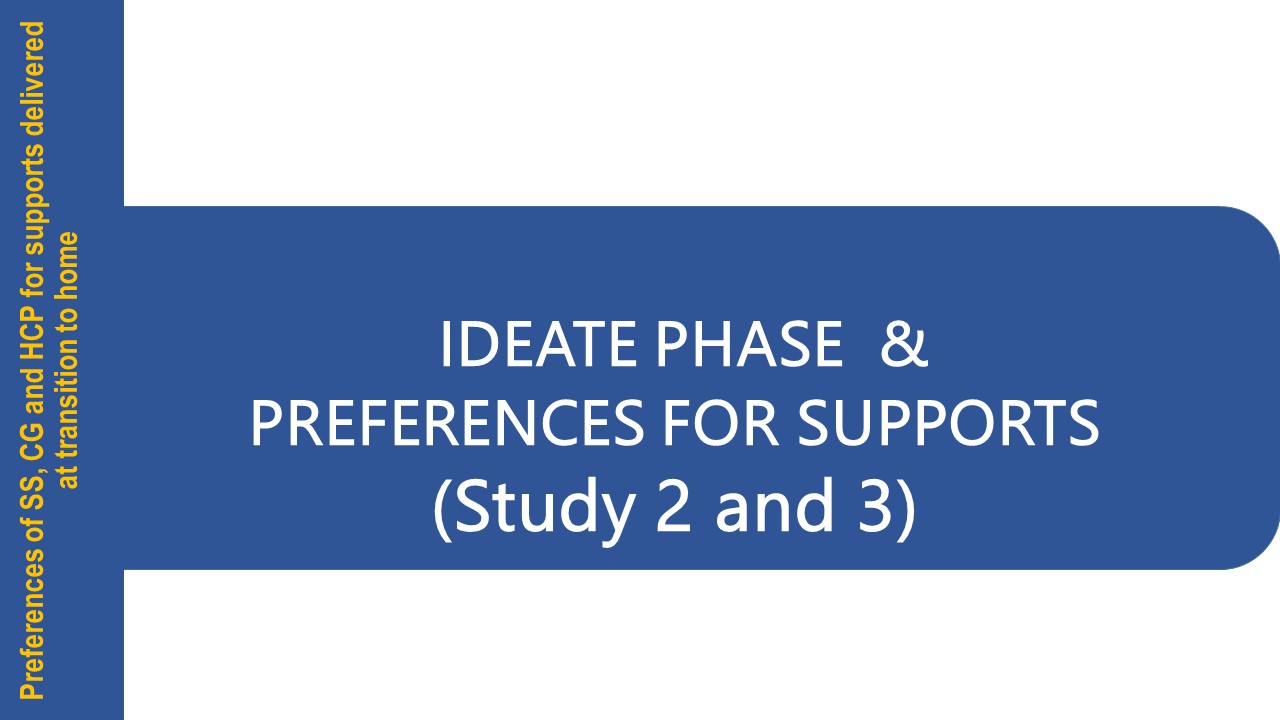

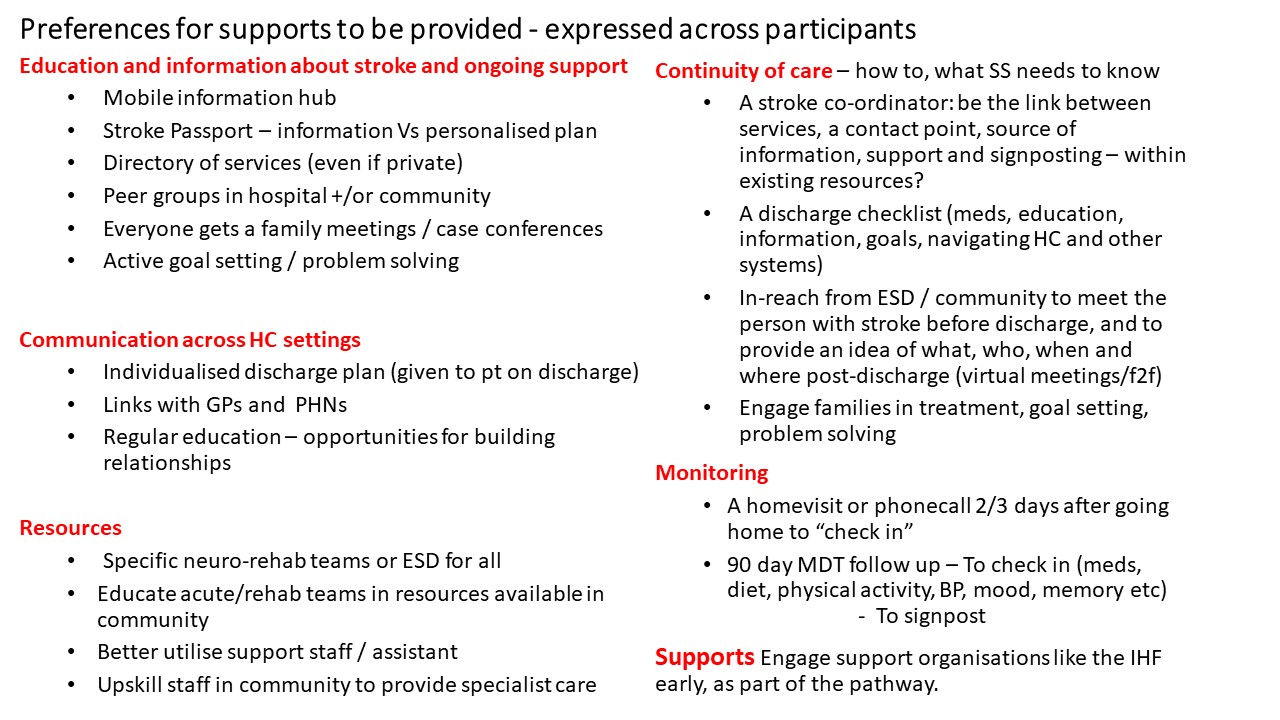

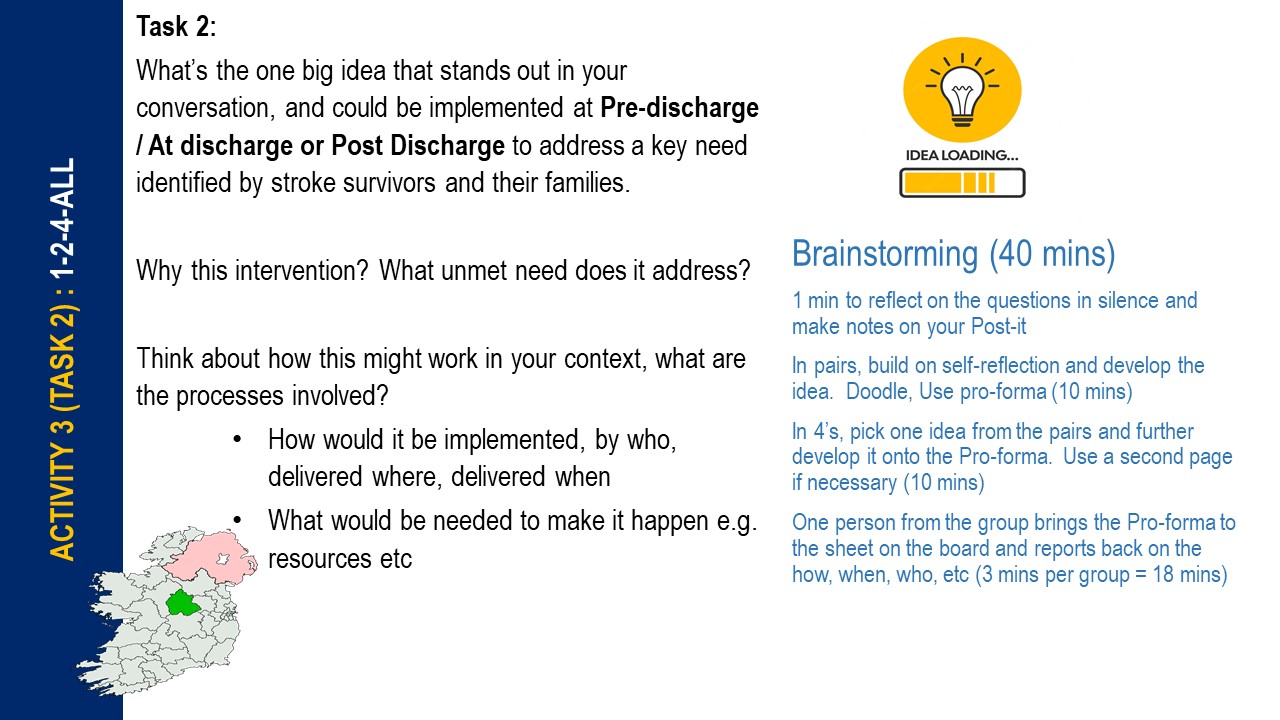

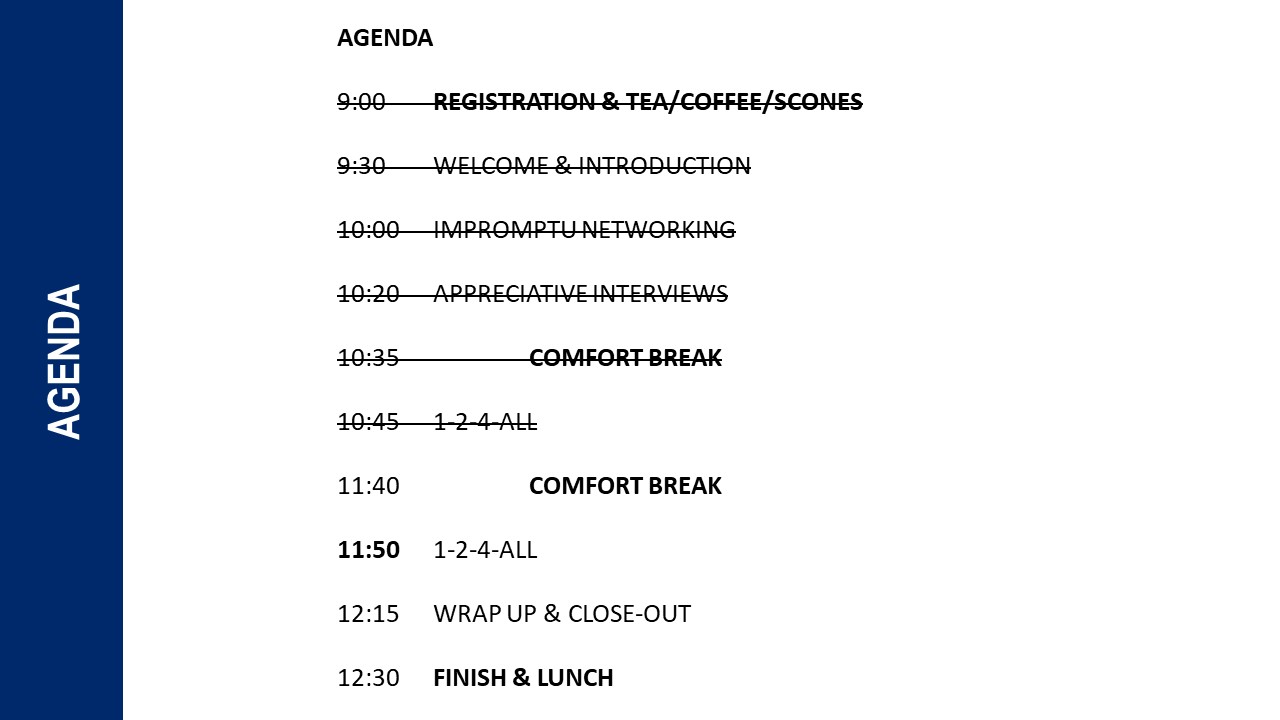

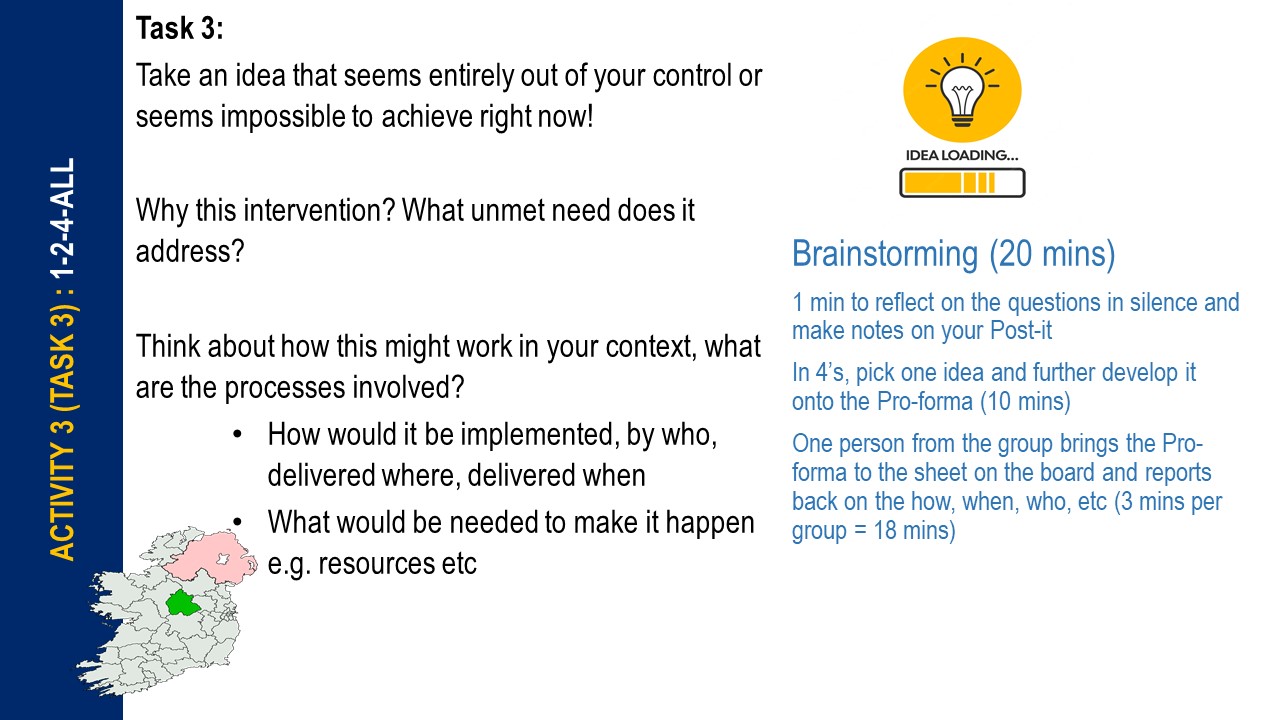

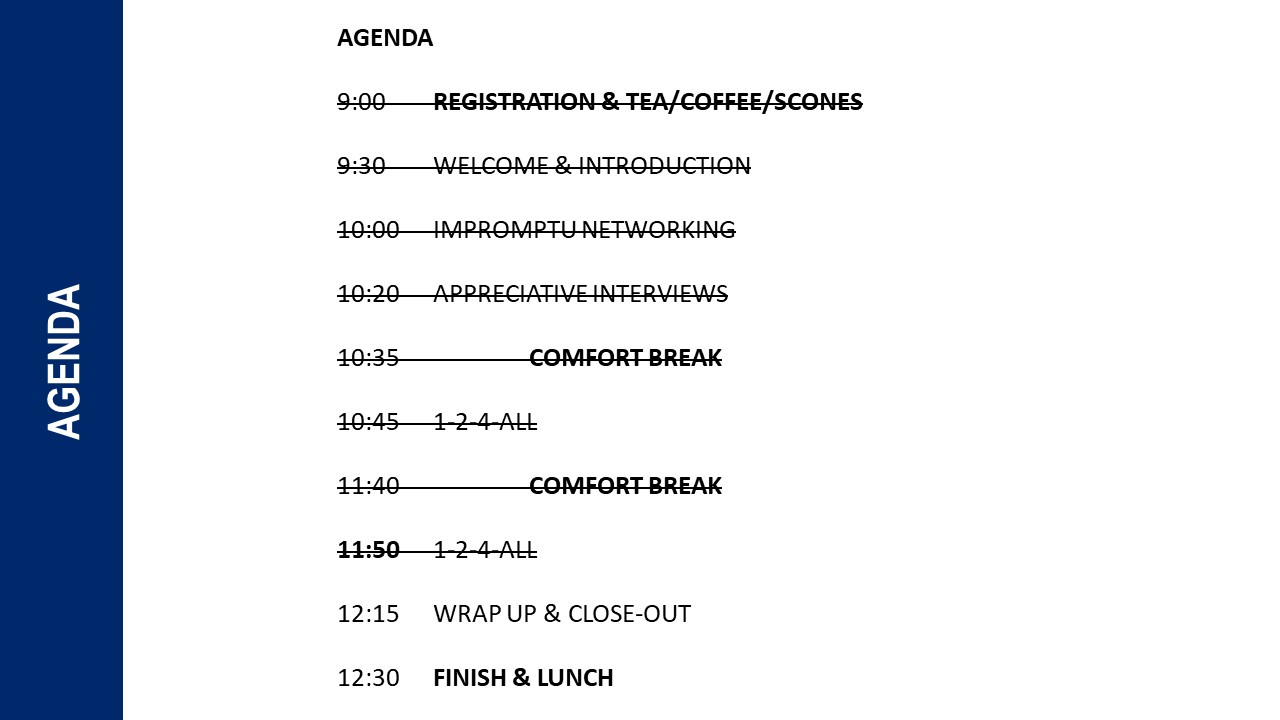
**

**
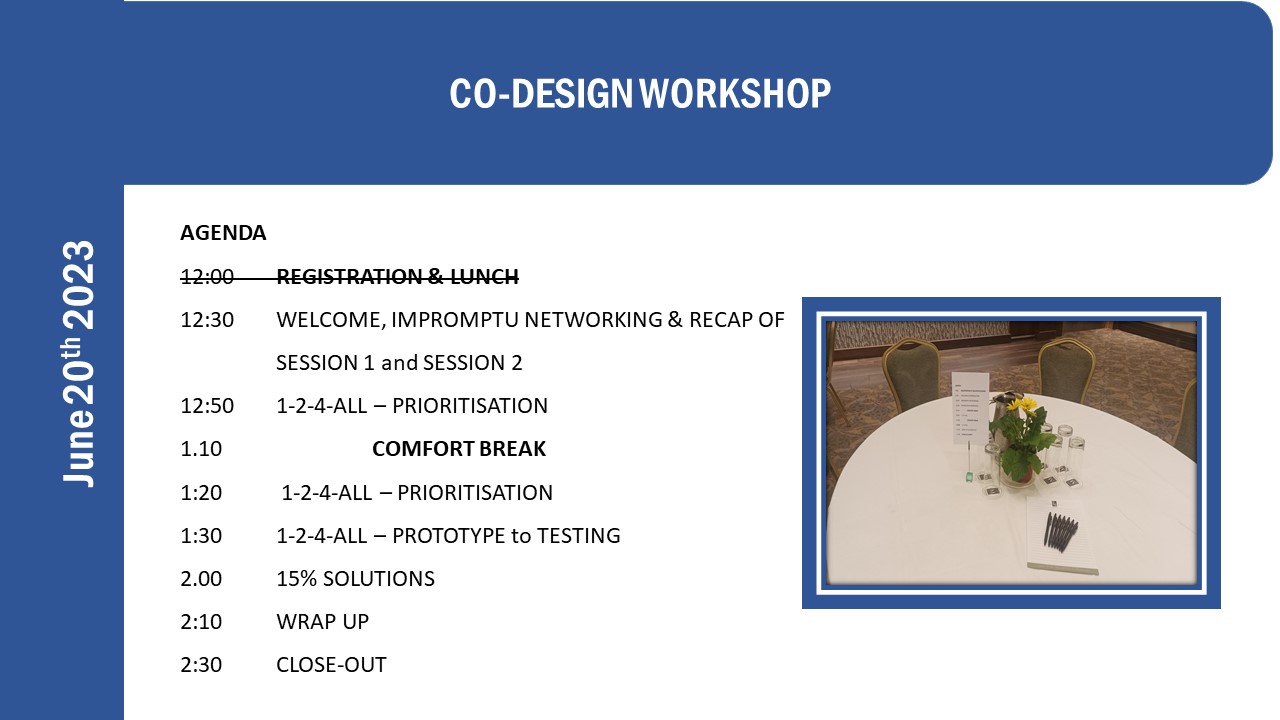
**

**
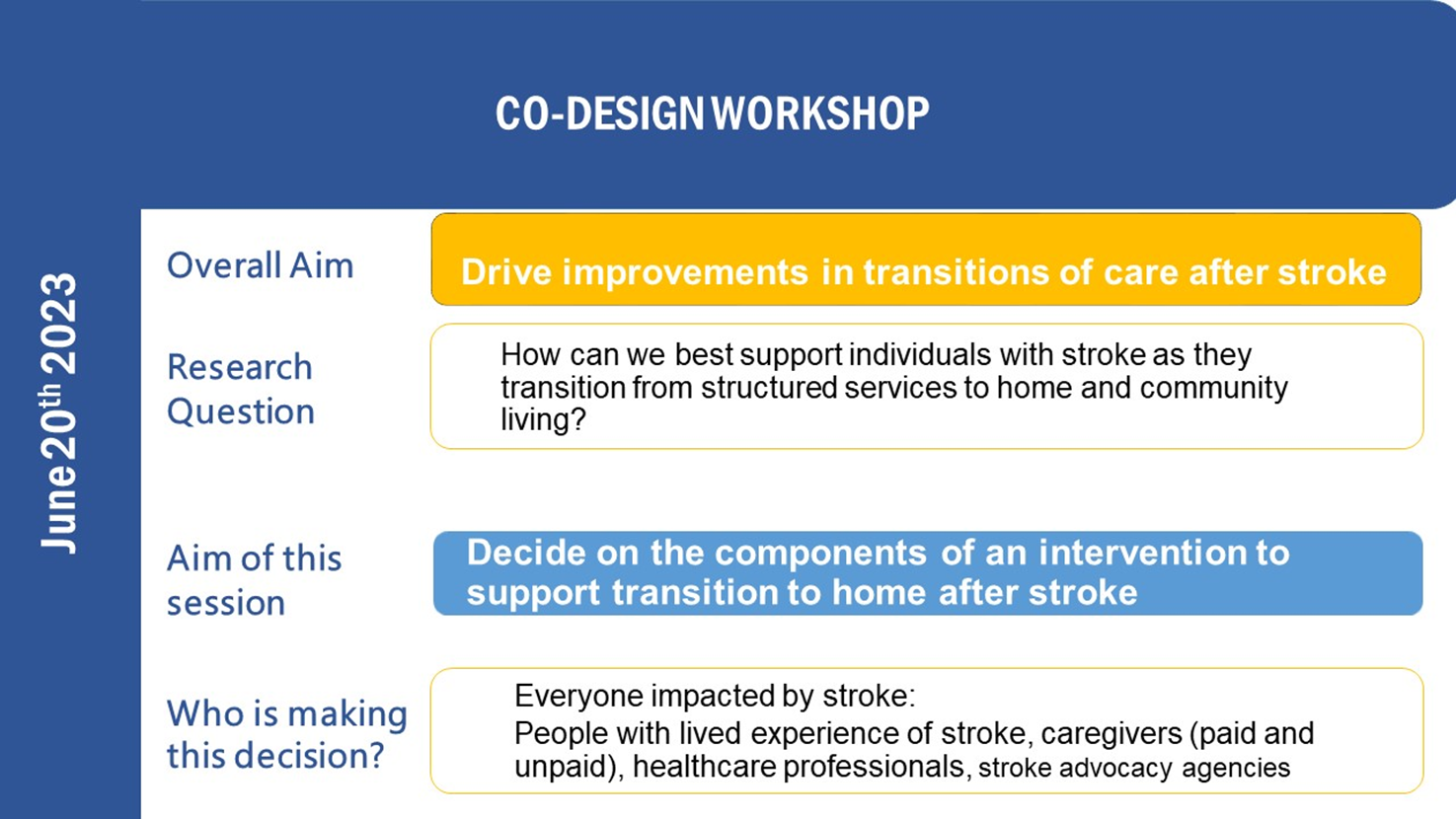
**

**
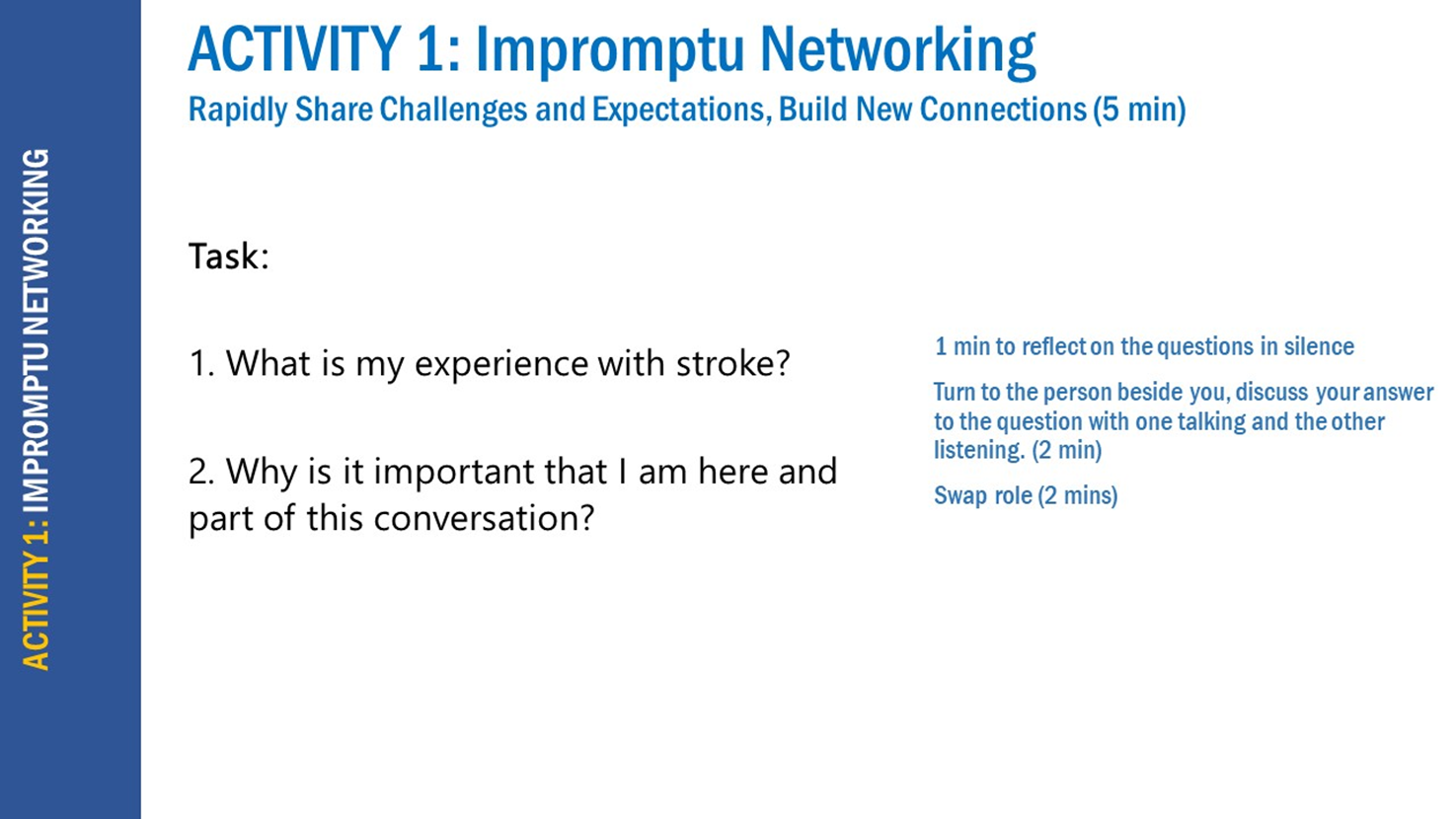
**

**
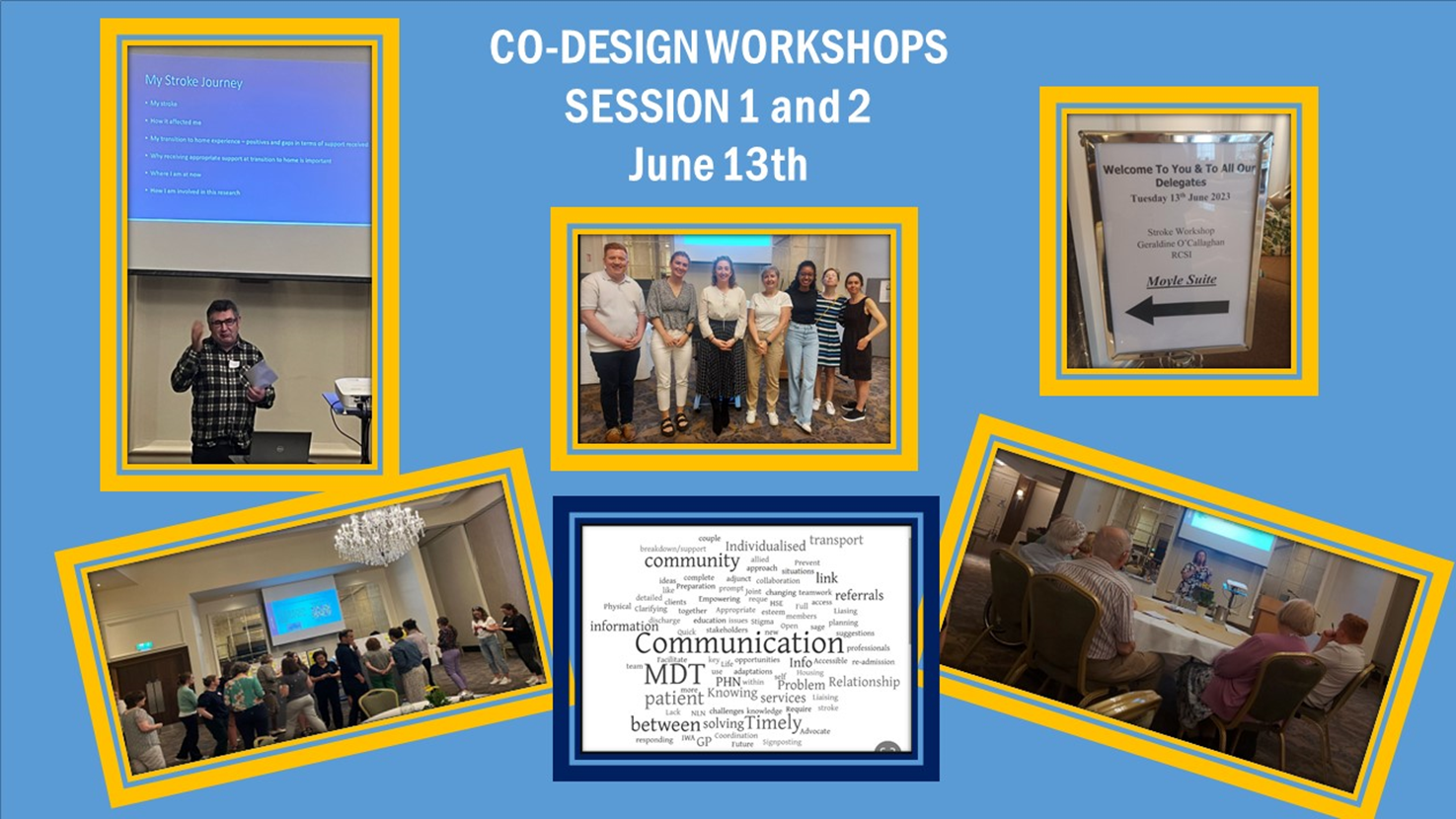
**

**
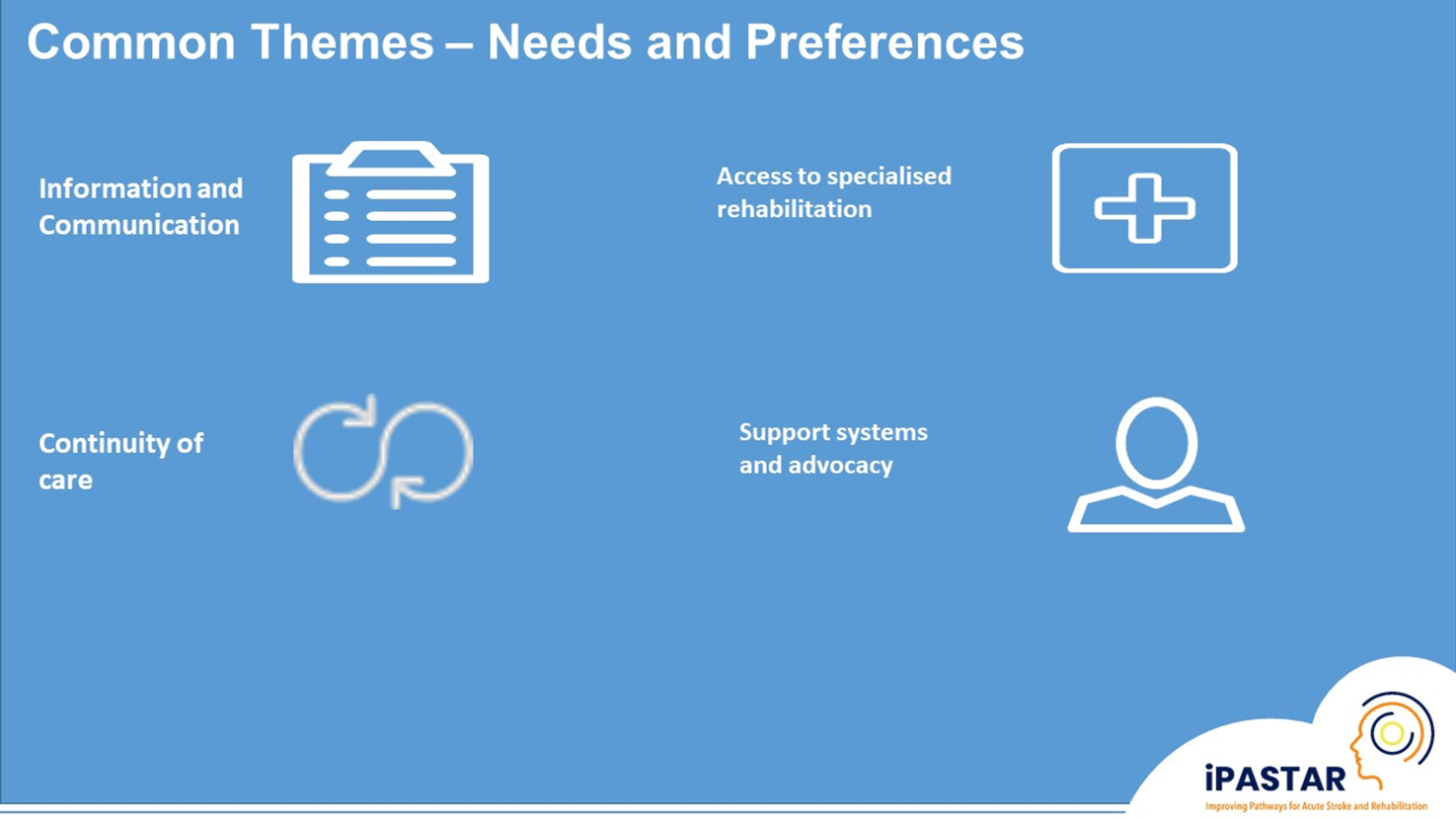
**

**
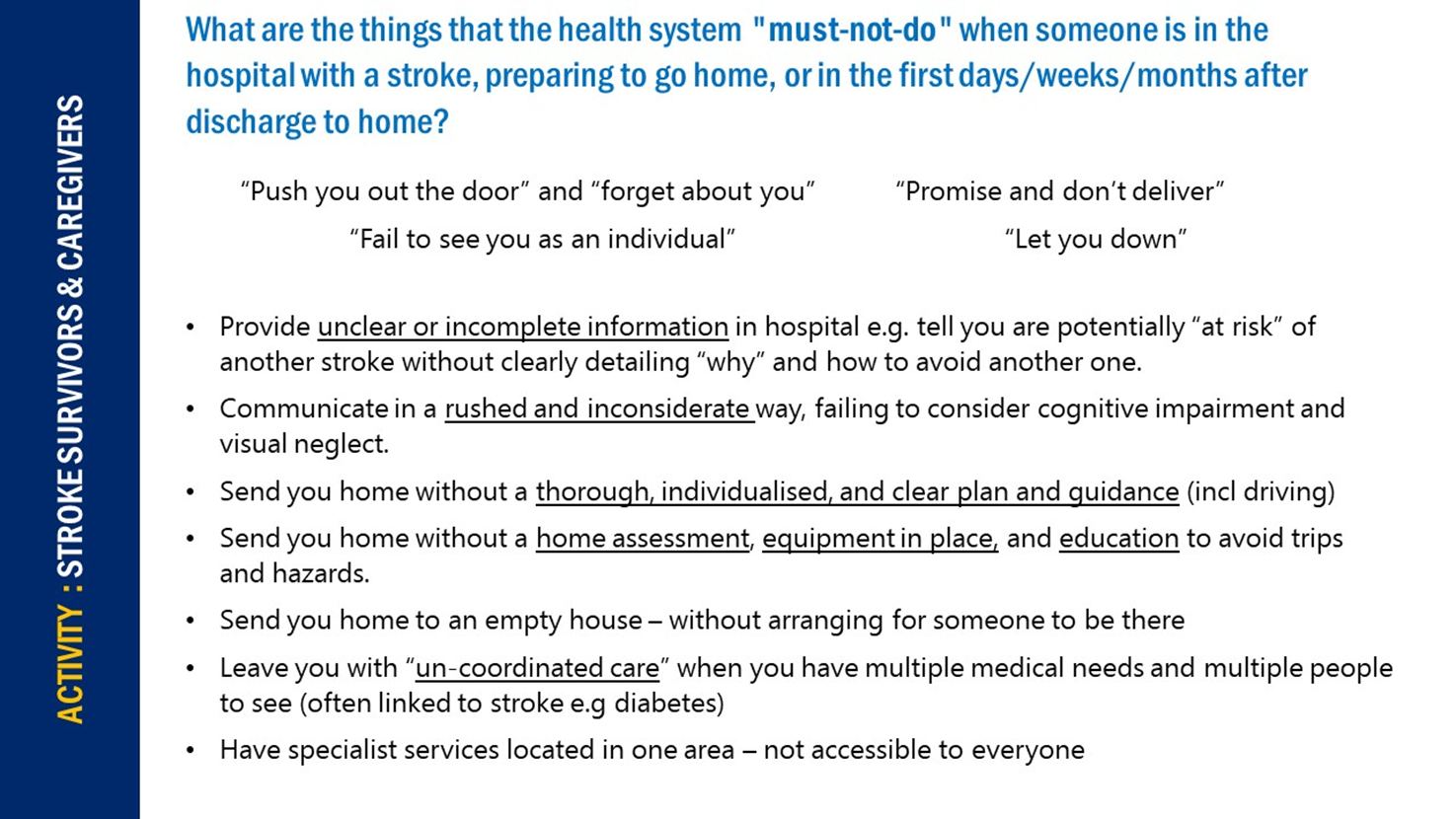
**

**
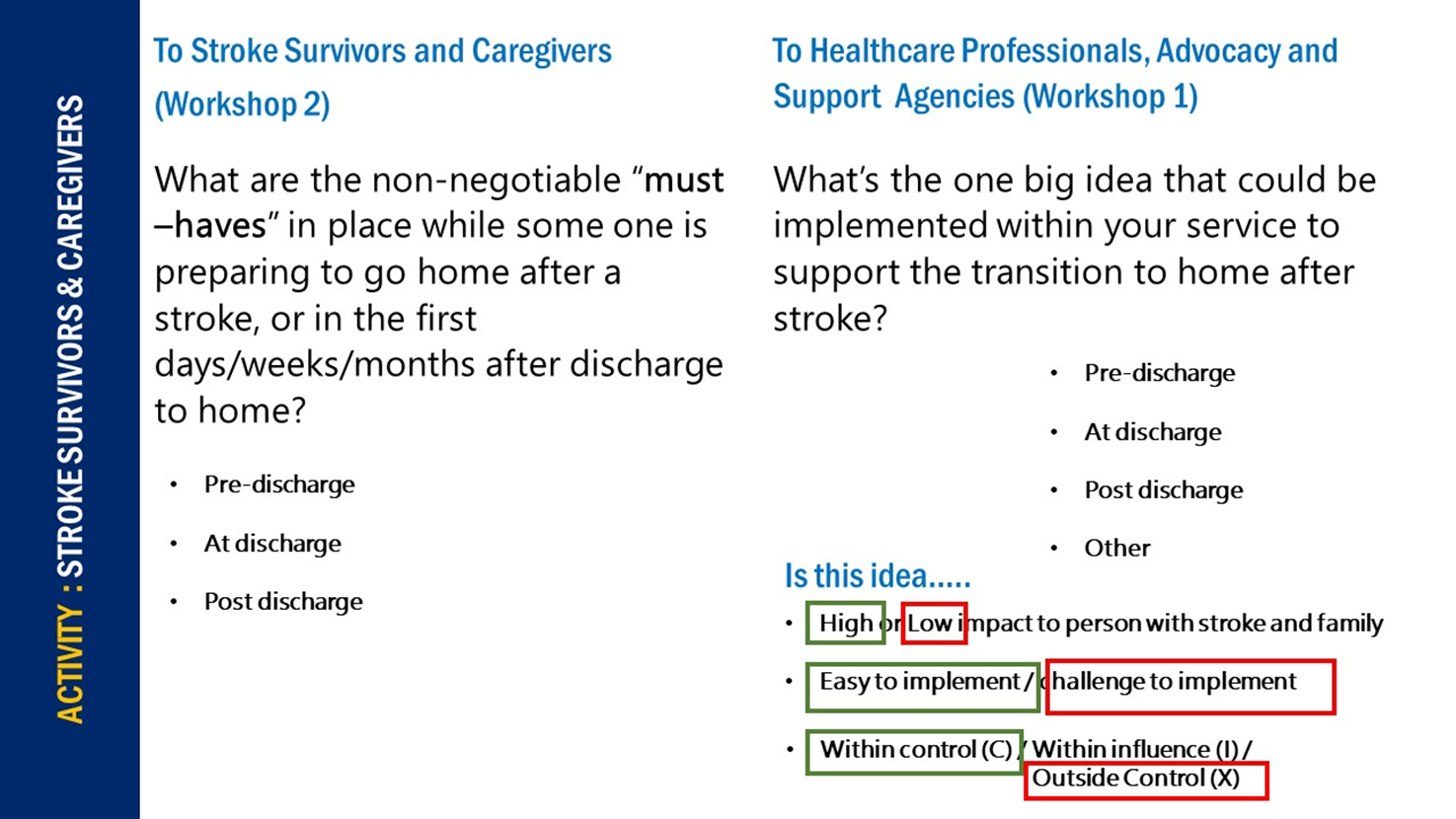
**

**
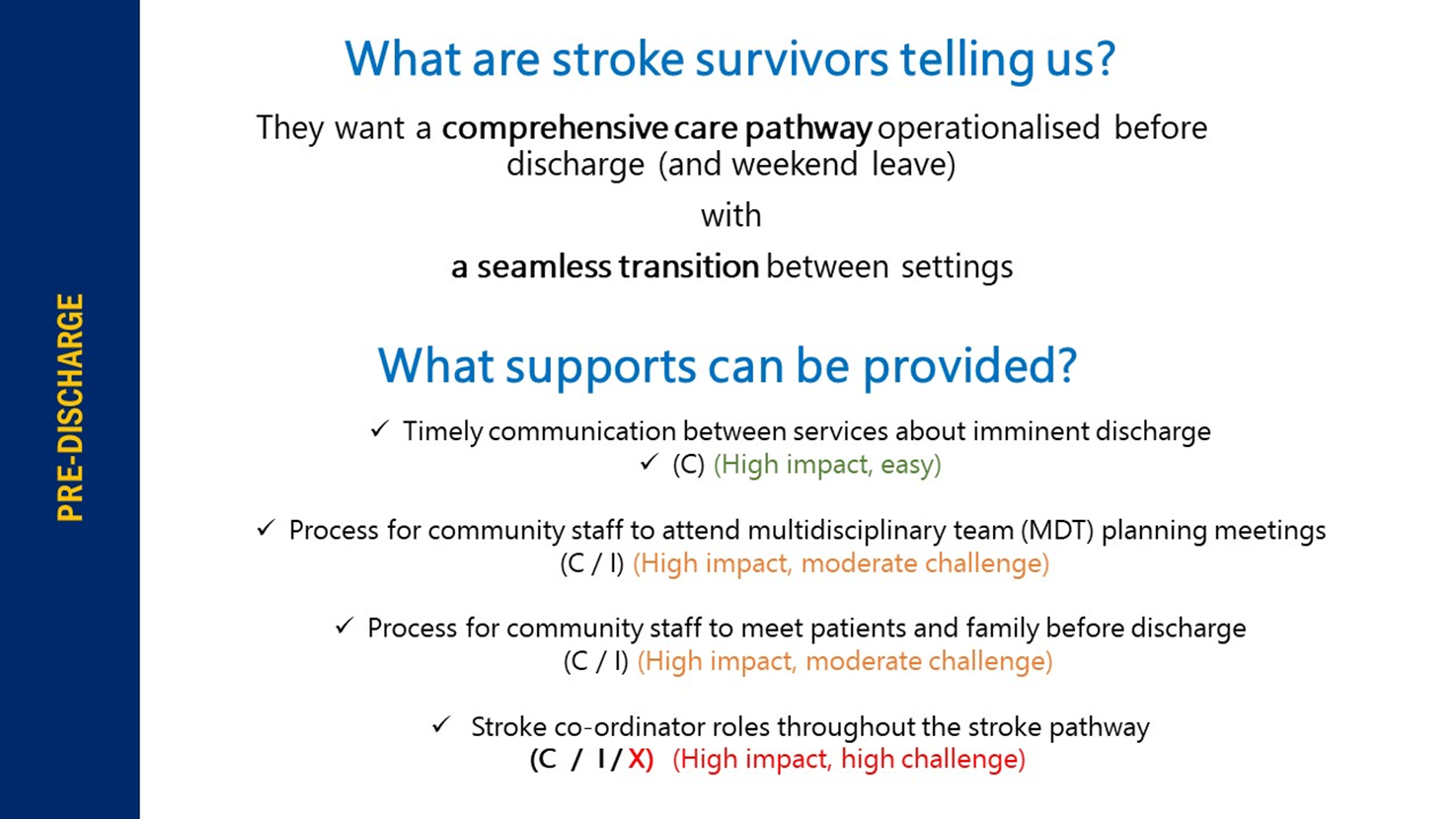
**

**
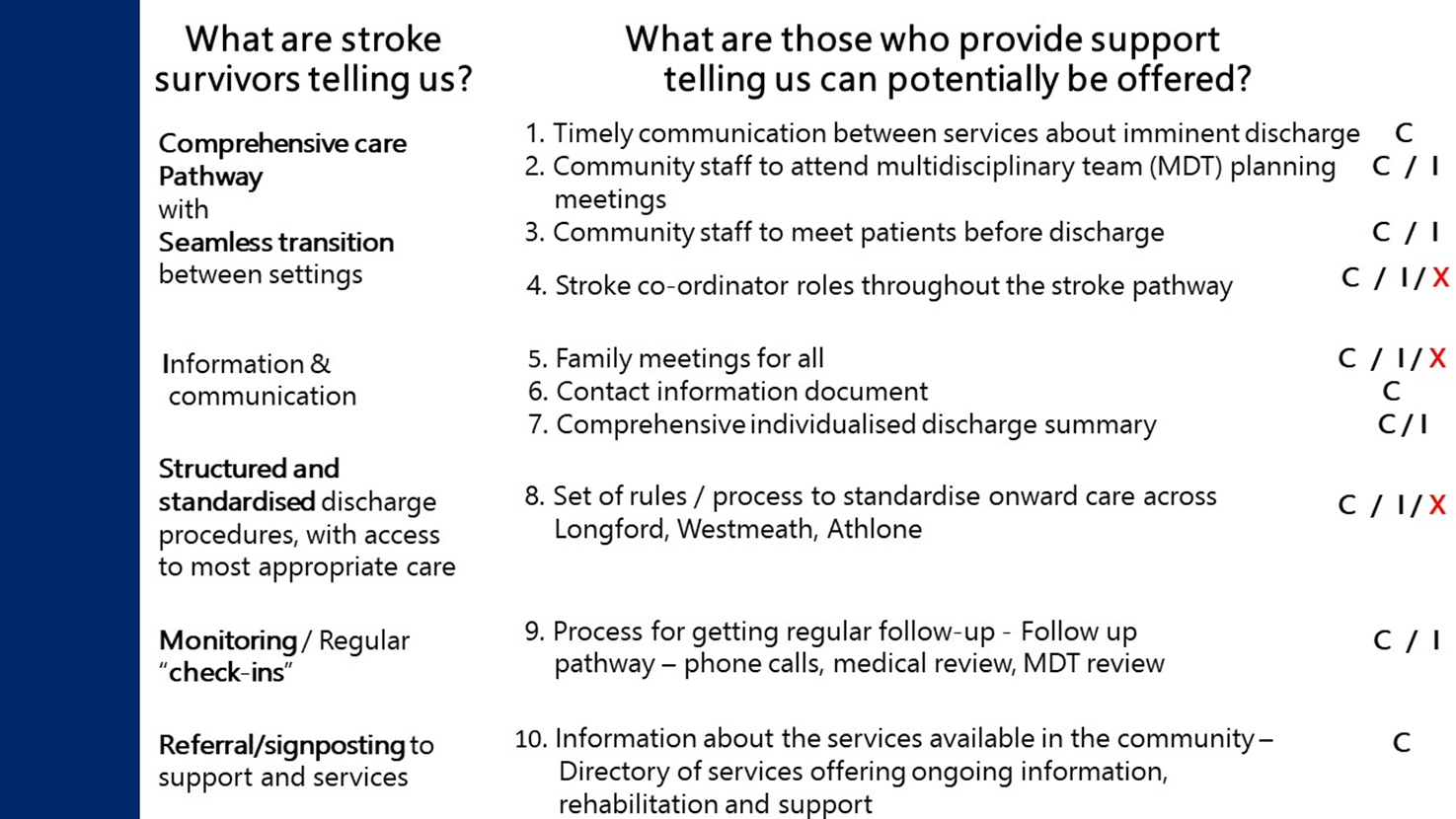
**

**
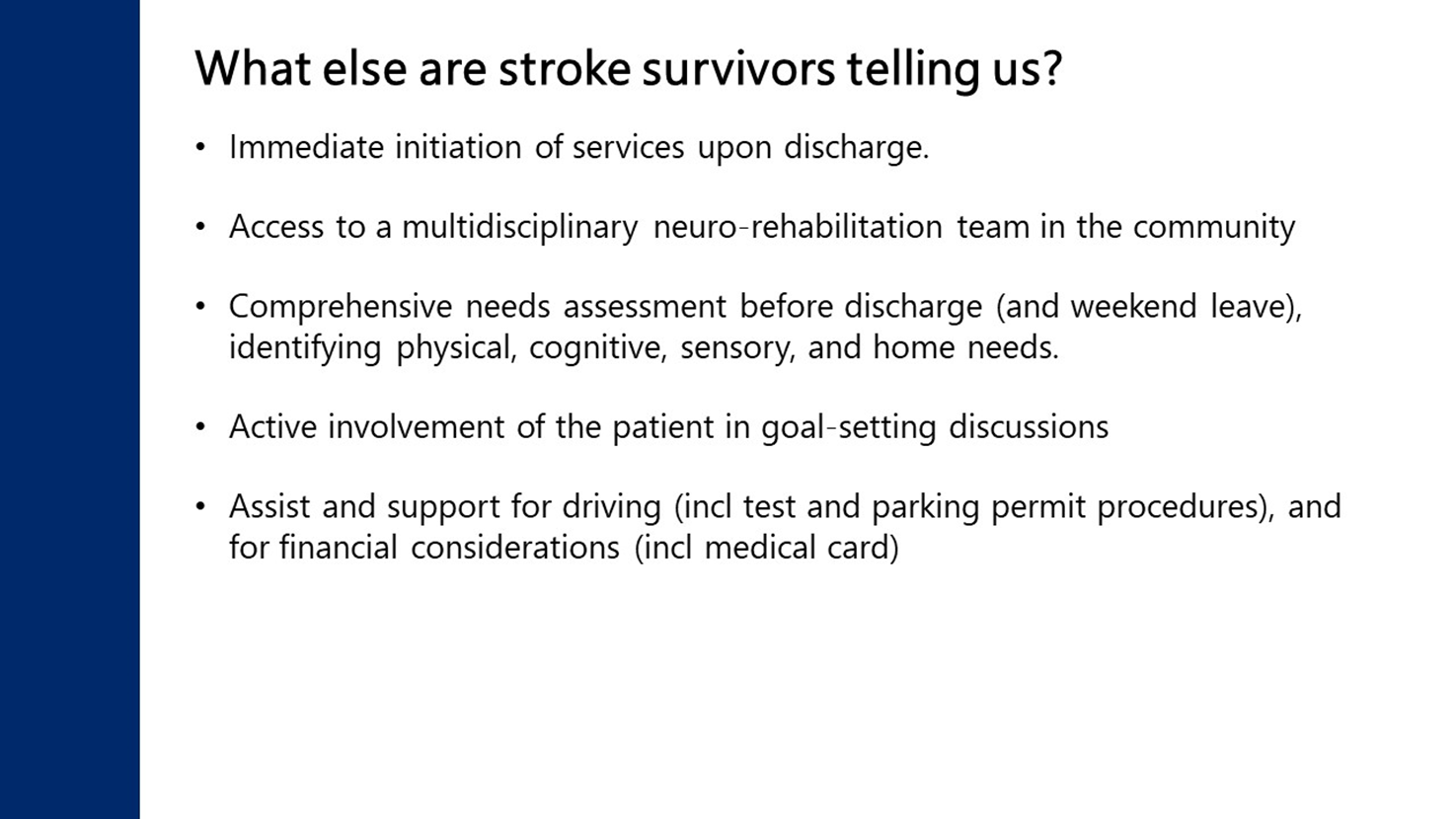
**

**
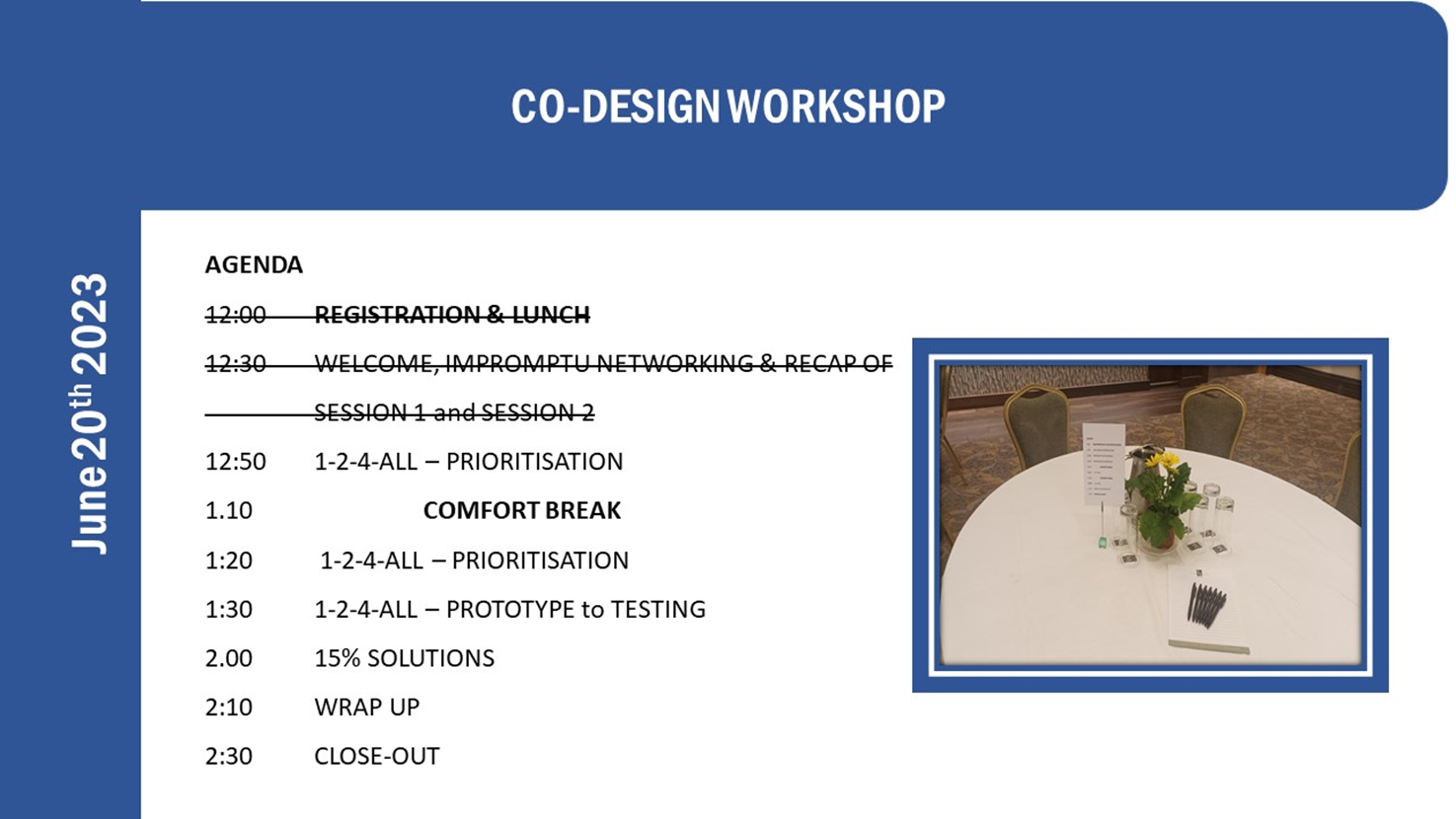
**

**
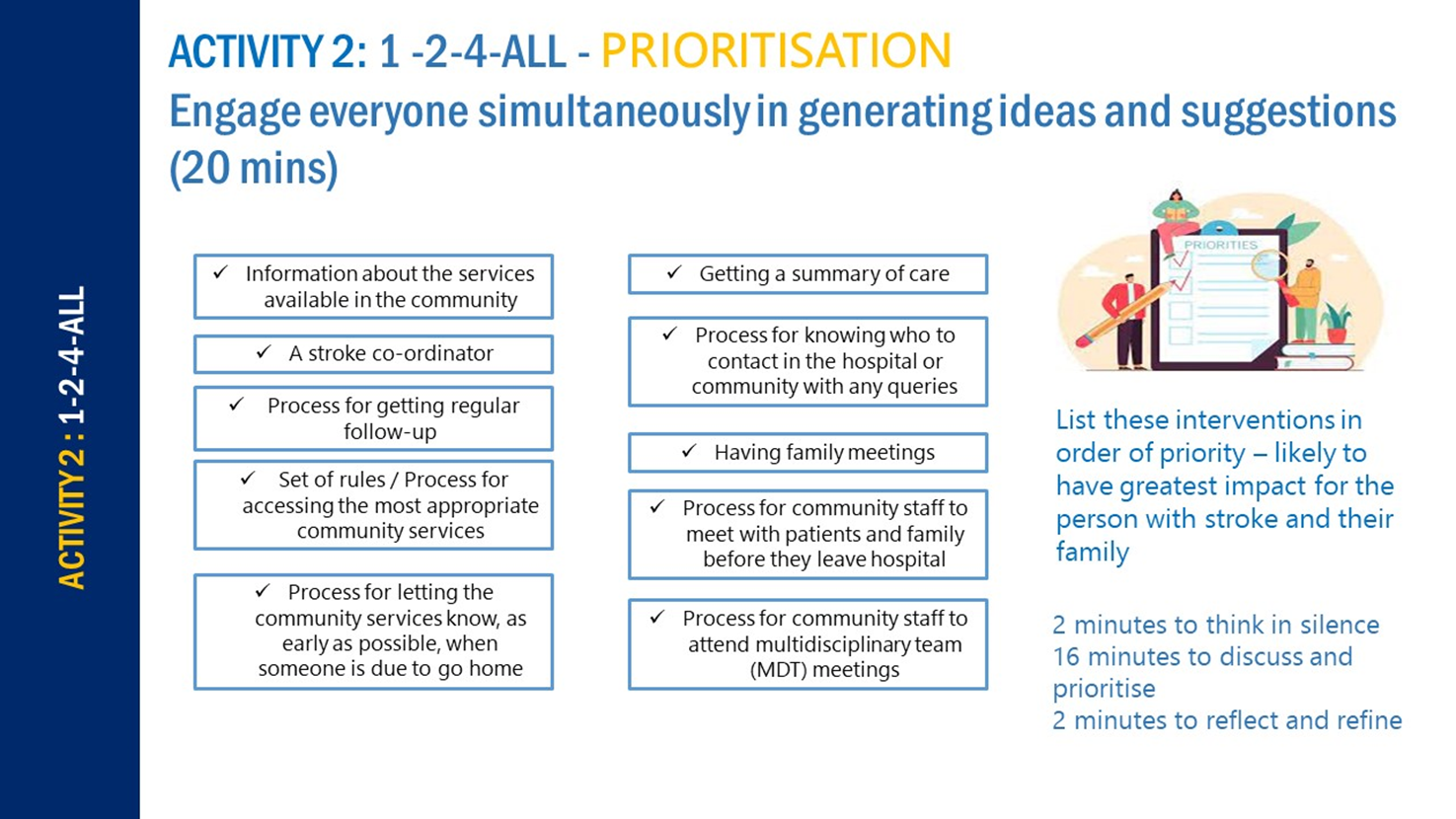
**

**
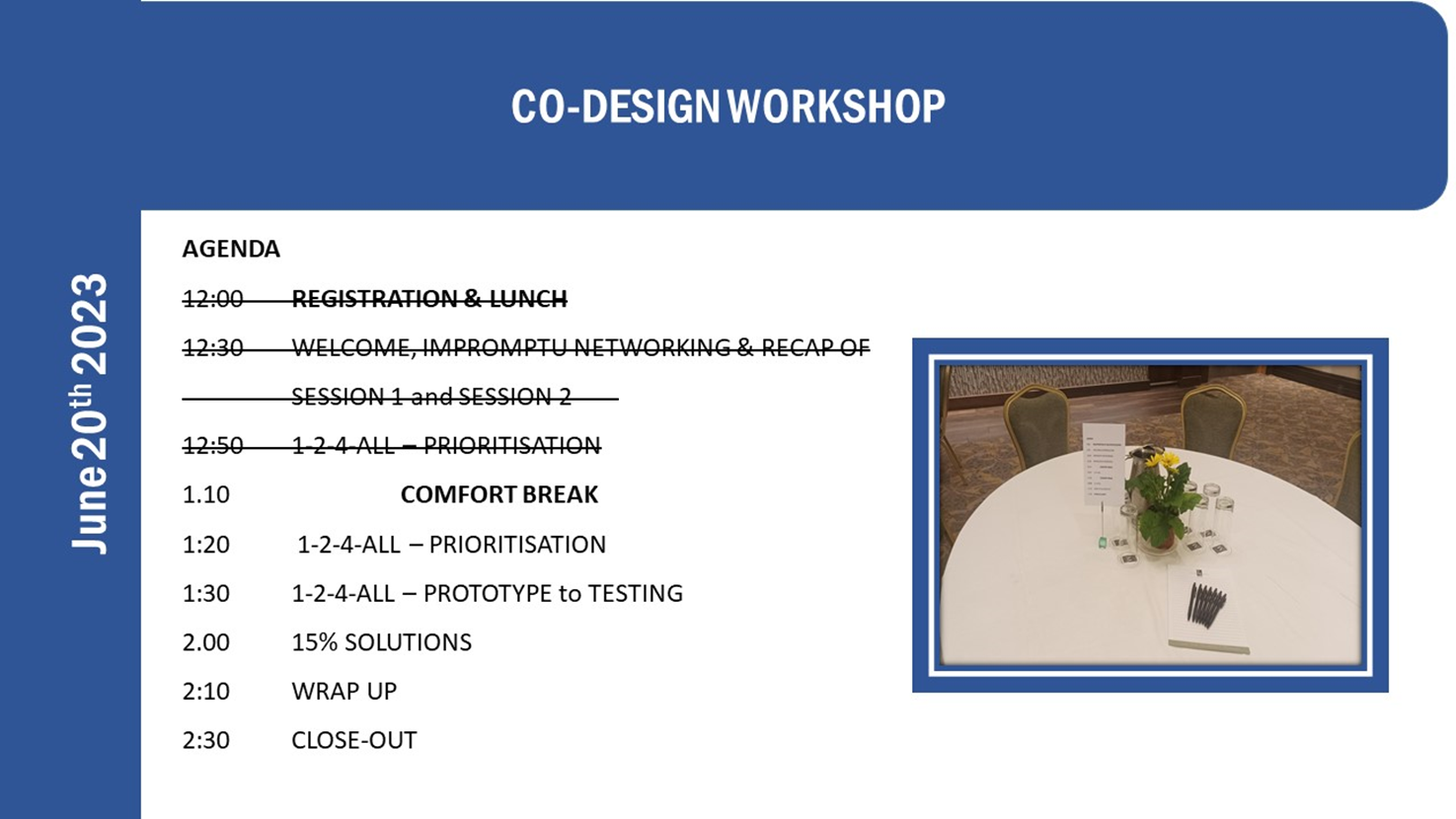
**

**
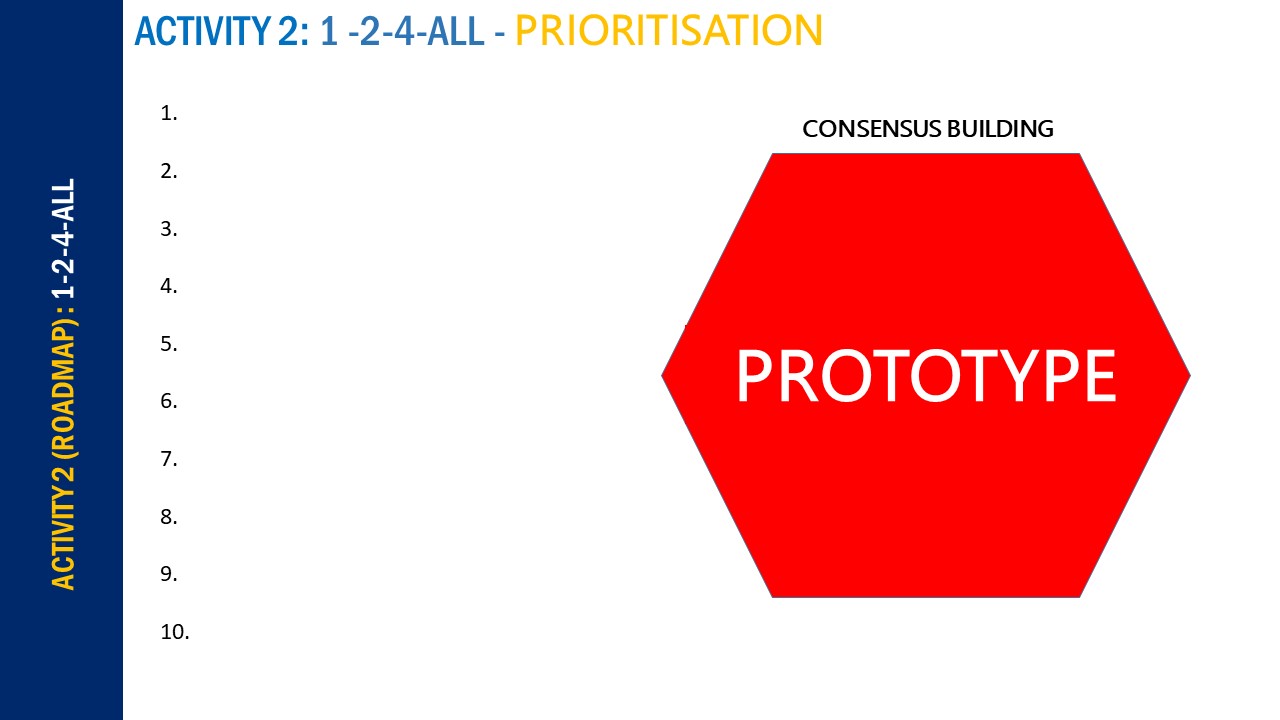
**

**
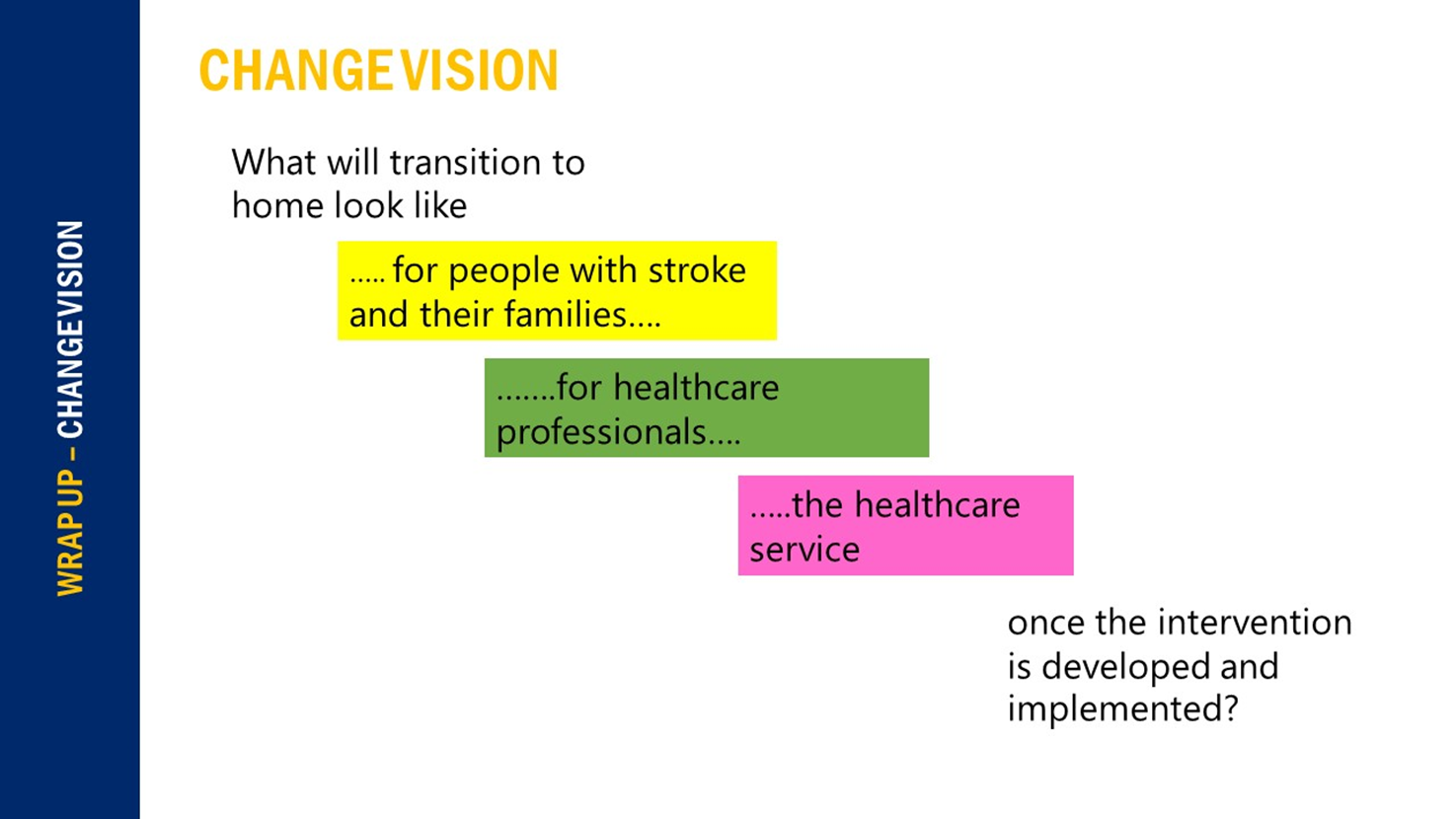
**

**
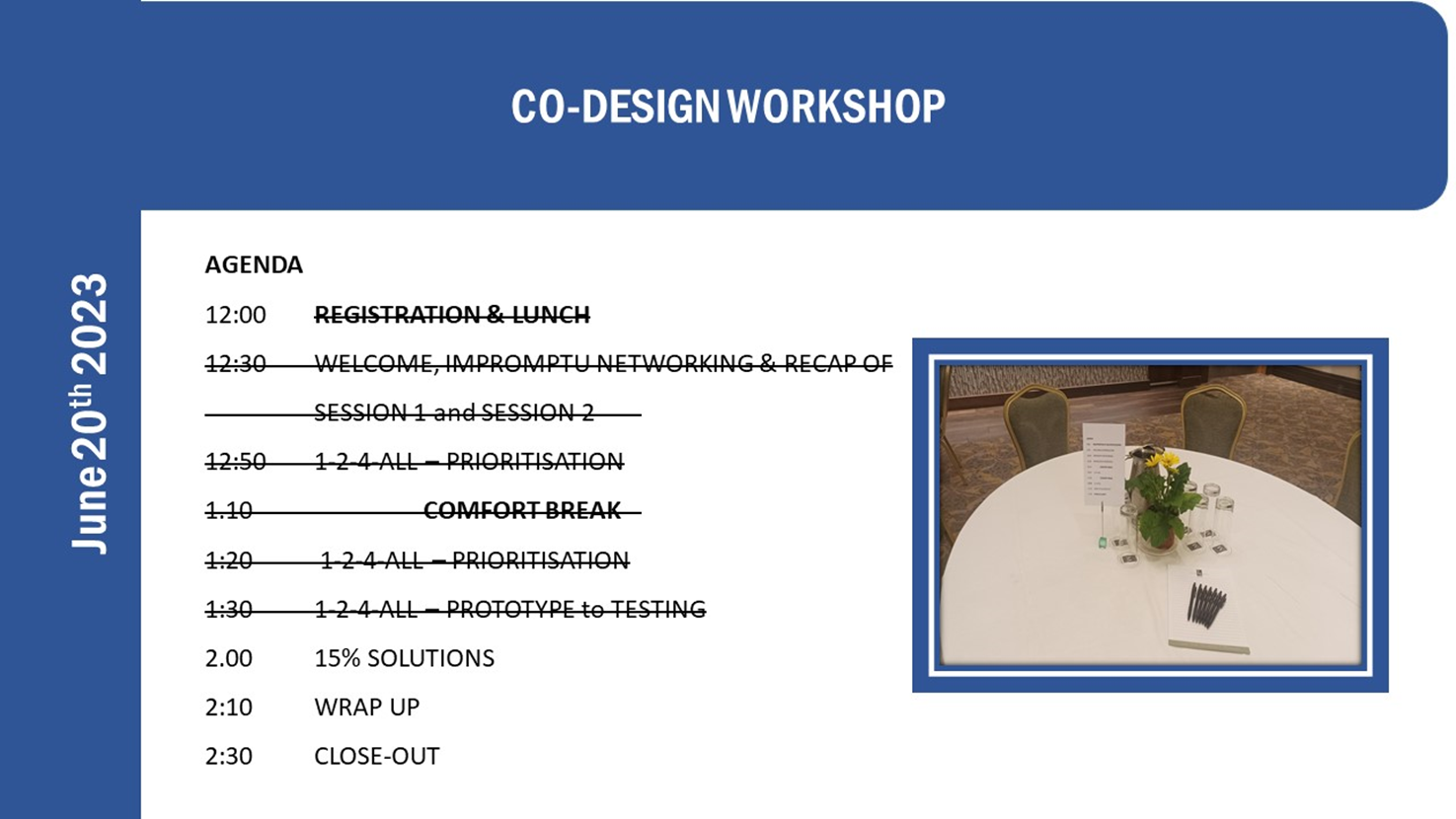
**

**
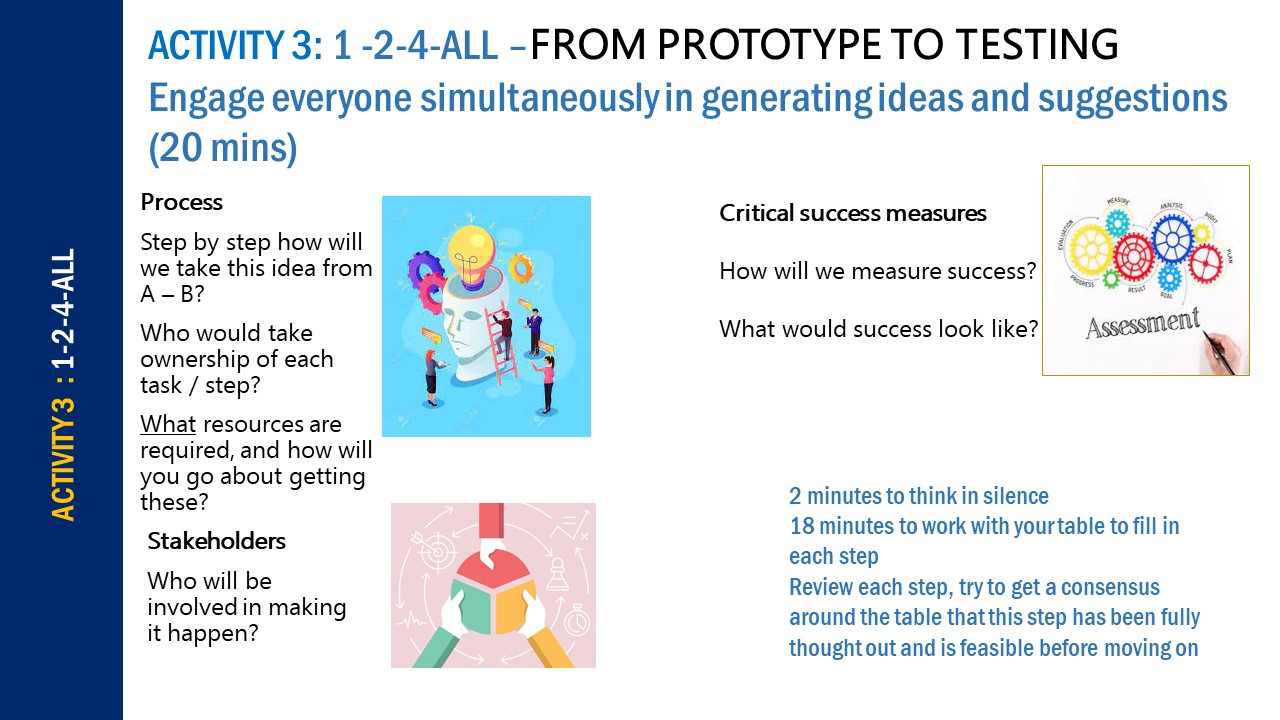
**

**
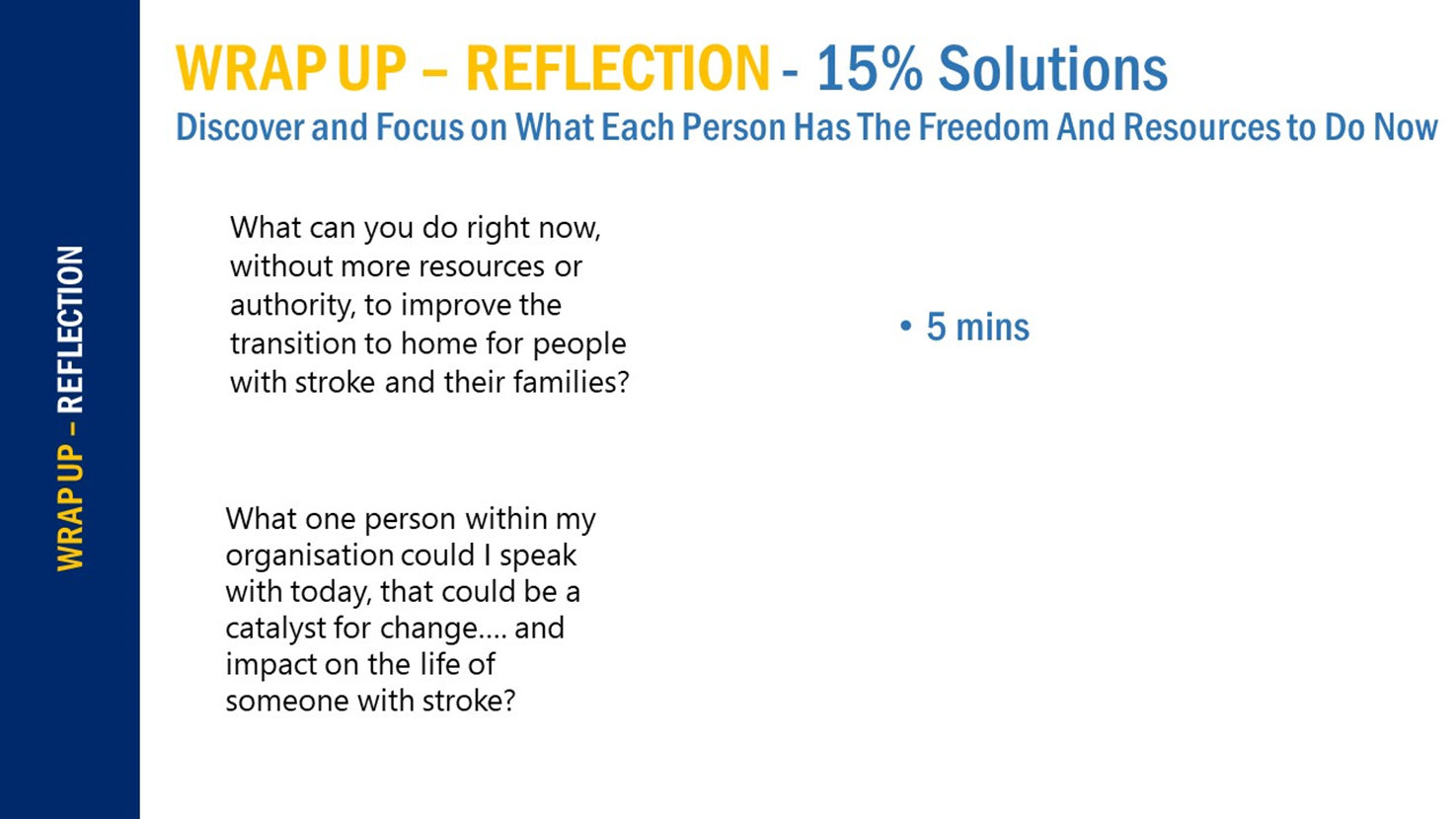
**

**Supplemental material III:** GRIPP2 short form

| Section and topic | Item | Reported on page No |
| --- | --- | --- |
| 1: Aim | Report the aim of PPI in the study | 7 |
| 2: Methods | Provide a clear description of the methods used for PPI in the study | 12 |
| 3: Study results | Outcomes—Report the results of PPI in the study, including both positive and negative outcomes | 16 |
| 4: Discussion and conclusions | Outcomes—Comment on the extent to which PPI influenced the study overall. Describe positive and negative effects | 20-21 |
| 5: Reflections/critical perspective | Comment critically on the study, reflecting on the things that went well and those that did not, so others can learn from this experience | Supplemental material |

PPIE=Patient and Public Involvement and Engagement

Discussion / Reflections.

By engaging with PPIE we helped improve the relevance, design and conduct of this research. Partnering with PPIE allowed us to identify future research priorities while further collaboration will inform a dissemination strategy. It is anticipated this level of engagement may lead to more patient-centred and better outcomes for people with stroke and their families as they transition back home and reintegrate into life after stroke.

**Supplemental material IV:** Aligning components into categories with narrative overview


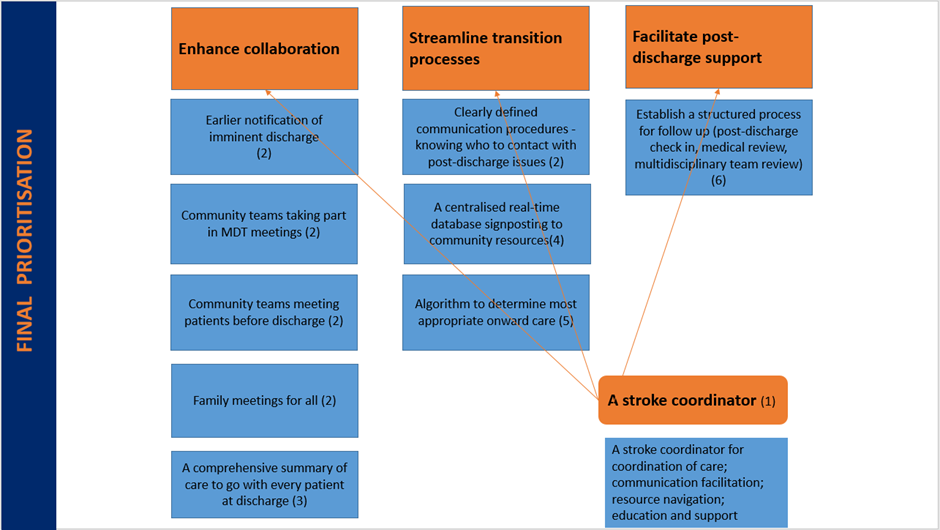


**Figure I** Aligning key components for an intervention designed to support transition to home after stroke into categories

**Enhance collaboration**: Co-design participants highlighted the need for improved communication and information sharing between healthcare providers, PWS, CGs, and support agencies to foster trust and continuity during transitions. They recommended pre-discharge family meetings, detailed discharge summaries, and extending community healthcare services into hospital settings to address this need. Strong cross-organisational leadership was suggested for effective implementation.

**Streamline transition processes**: Transition-to-home after stroke was described as complex due to a lack of available information, delays, and changing needs. Co-design participants recommended creating an algorithm that would inform and guide HCPs in decision making regarding referral to the most appropriate post-discharge rehabilitation and support services, a communication protocol to enable PWS address post-discharge issues, and a centralised database of community-based resources. These processes would reduce uncertainty and enhance collaboration by establishing communication channels for sharing important information.

**Facilitate post-discharge support**: Many PWS and CGs recalled feeling abandoned upon returning home, leading to missed opportunities for timely interventions and poorer health outcomes. Access to post-discharge check-in, regular medical follow-ups, and multidisciplinary reviews was recommended by co-design participants.

**A stroke co-ordinator as overarching support**: PWS and CGs spoke of being overwhelmed as they recalled navigating the complexity of the healthcare system and coordinating their own care post-discharge. The proposal of a stroke co-ordinator as a 'single point of contact' for patient-centred care coordination, fostering collaboration among care providers, and coordinating follow-up appointments, was a source of much discussion around scope, as there were distinct roles and responsibilities expected by the different stakeholder groups.
